# Supplementary material for: Cell membrane glycan contents are biochemical factors that constitute a kinetic barrier to viral particle uptake in a protein-nonspecific manner
Source: eLife. 2025 Oct 28;14:RP101175. doi: 10.7554/eLife.101175 (PMC12563552; doi:10.7554/eLife.101175)
Supplement: Supplementary file 2. — Length of amino acid sequences in ectodomains as well as ratio of glycosylation site per amino acid are listed for all molecules. [file elife-101175-supp2.pdf]

| UniProt Entry | Protein # | Gene name | Protein names | Total Etcodomain (AA) | # of membrane-spanning | Max Etcodomain (AA) | GlycoEP(N,O) Predicted Glycosylation /AA | Net N/O Glyc Predicted Glycosylation /AA | Mean (Glyco EP & Net N/O Glyc) |
|---|---|---|---|---|---|---|---|---|---|
| Q8NE79 | 0 | BVES | Blood vessel epica | 47 | 1 | 47 | 0.14893617 | 0.042553191 | 0.095744681 |
| P21453 | 1 | S1PR1 | Sphingosine 1-ph | 79 | 4 | 45 | 0.101265823 | 0.088607595 | 0.094936709 |
| Q9Y6R1 | 2 | SLC4A4 | Electrogenic sodiu | 137 | 5 | 82 | 0.102189781 | 0.02919708 | 0.065693431 |
| P35498 | 3 | SCN1A | Sodium channel p | 323 | 16 | 97 | 0.034055728 | 0.027863777 | 0.030959752 |
| P30872 | 4 | SSTR1 | Somatostatin rece | 93 | 4 | 55 | 0.150537634 | 0.139784946 | 0.14516129 |
| P32745 | 5 | SSTR3 | Somatostatin rece | 92 | 4 | 42 | 0.119565217 | 0.130434783 | 0.125 |
| O95259 | 6 | KCNH1 | Potassium voltage | 95 | 4 | 48 | 0.031578947 | 0.021052632 | 0.026315789 |
| Q7Z6M3 | 7 | MILR1 | Allergin-1 (Allergy | 207 | 1 | 207 | 0.082125604 | 0.028985507 | 0.055555556 |
| Q8NHS3 | 8 | MFSD8 | Major facilitator s | 110 | 6 | 53 | 0.027272727 | 0.018181818 | 0.022727273 |
| Q96RD7 | 9 | PANX1 | Pannexin-1 | 71 | 2 | 44 | 0.014084507 | 0.014084507 | 0.014084507 |
| O15547 | 10 | P2RX6 | P2X purinoceptor | 272 | 1 | 272 | 0.025735294 | 0.022058824 | 0.023897059 |
| O94991 | 11 | SLITRK5 | SLIT and NTRK-li | 623 | 1 | 623 | 0.036918138 | 0.051364366 | 0.044141252 |
| Q9UKZ4 | 12 | TENM1 | Teneurin-1 (Ten- | 2379 | 1 | 2379 | 0.023539302 | 0.016813787 | 0.020176545 |
| Q9Y275 | 13 | TNFSF13B | Tumor necrosis fa | 217 | 1 | 217 | 0.023041475 | 0.023041475 | 0.023041475 |
| P28827 | 14 | PTPRM | Receptor-type tyr | 721 | 1 | 721 | 0.05963939 | 0.030513176 | 0.045076283 |
| P15151 | 15 | PVR | Poliovirus recepto | 322 | 1 | 322 | 0.074534161 | 0.02484472 | 0.049689441 |
| Q9UHW9 | 16 | SLC12A6 | Solute carrier fam | 158 | 5 | 107 | 0.044303797 | 0.03164557 | 0.037974684 |
| Q13433 | 17 | SLC39A6 | Zinc transporter Z | 358 | 4 | 296 | 0.055865922 | 0.136871508 | 0.096368715 |
| P48764 | 18 | SLC9A3 | Sodium/hydrogen | 102 | 6 | 30 | 0.058823529 | 0.039215686 | 0.049019608 |
| Q9BZD2 | 19 | SLC29A3 | Equilibrative nucle | 71 | 5 | 30 | 0.028169014 | 0.070422535 | 0.049295775 |
| P48029 | 20 | SLC6A8 | Sodium- and chlo | 150 | 6 | 70 | 0.06 | 0.033333333 | 0.046666667 |
| P23975 | 21 | SLC6A2 | Sodium-depender | 133 | 5 | 66 | 0.045112782 | 0.052631579 | 0.04887218 |
| Q86VE9 | 22 | SERINC5 | Serine incorporato | 126 | 5 | 52 | 0.03968254 | 0.007936508 | 0.023809524 |
| Q6ZMC9 | 23 | SIGLEC15 | Sialic acid-binding | 243 | 1 | 243 | 0.041152263 | 0.004115226 | 0.022633745 |
| Q3KNW5 | 24 | SLC10A6 | Solute carrier fam | 53 | 5 | 28 | 0.094339623 | 0.113207547 | 0.103773585 |
| Q9Y3P8 | 25 | SIT1 | Signaling thresho | 15 | 1 | 15 | 0.066666667 | 0.066666667 | 0.066666667 |
| Q7RTT9 | 26 | SLC29A4 | Equilibrative nucle | 166 | 6 | 67 | 0.054216867 | 0.012048193 | 0.03313253 |
| Q99500 | 27 | S1PR3 | Sphingosine 1-ph | 82 | 4 | 39 | 0.024390244 | 0.06097561 | 0.042682927 |
| Q9NYZ4 | 28 | SIGLEC8 | Sialic acid-binding | 346 | 1 | 346 | 0.095375723 | 0.031791908 | 0.063583815 |
| O43556 | 29 | SGCE | Epsilon-sarcoglyca | 316 | 1 | 316 | 0.03164557 | 0.022151899 | 0.026898734 |
| Q9NY15 | 30 | STAB1 | Stabilin-1 (Fascic | 2452 | 1 | 2452 | 0.037520392 | 0.05954323 | 0.048531811 |
| Q9UIW2 | 31 | PLXNA1 | Plexin-A1 (Semap | 1217 | 1 | 1217 | 0.030402629 | 0.023007395 | 0.026705012 |
| Q9Y6M7 | 32 | SLC4A7 | Sodium bicarbona | 809 | 6 | 607 | 0.035846724 | 0.082818294 | 0.059332509 |
| P31431 | 33 | SDC4 | Syndecan-4 (SYN | 126 | 1 | 126 | 0.023809524 | 0.111111111 | 0.067460317 |
| Q9H156 | 34 | SLITRK2 | SLIT and NTRK-li | 599 | 1 | 599 | 0.035058431 | 0.05509182 | 0.045075125 |
| Q8WWQ8 | 35 | STAB2 | Stabilin-2 (FAS1 E | 2438 | 1 | 2438 | 0.034454471 | 0.066447908 | 0.050451189 |
| Q9UP52 | 36 | TFR2 | Transferrin recept | 696 | 1 | 696 | 0.028735632 | 0.025862069 | 0.027298851 |
| Q7RTX1 | 37 | TAS1R1 | Taste receptor typ | 595 | 4 | 546 | 0.028571429 | 0.011764706 | 0.020168067 |
| Q96JQ0 | 38 | DCHS1 | Protocadherin-16 | 2897 | 1 | 2897 | 0.05246807 | 0.006558509 | 0.02951329 |
| P08922 | 39 | ROS1 | Proto-oncogene t | 1831 | 1 | 1831 | 0.044238121 | 0.024030584 | 0.034134353 |
| Q9H015 | 40 | SLC22A4 | Solute carrier fam | 127 | 6 | 99 | 0.023622047 | 0.023622047 | 0.023622047 |
| Q13530 | 41 | SERINC3 | Serine incorporato | 174 | 5 | 95 | 0.040229885 | 0.028735632 | 0.034482759 |
| Q16586 | 42 | SGCA | Alpha-sarcoglycar | 266 | 1 | 266 | 0.060150376 | 0.033834586 | 0.046992481 |
| Q495A1 | 43 | TIGIT | T-cell immunorece | 119 | 1 | 119 | 0 | 0.016806723 | 0.008403361 |
| Q6UW88 | 44 | EPGN | Epigen (Epithelial | 87 | 1 | 87 | 0.057471264 | 0.08045977 | 0.068965517 |
| Q9UJ14 | 45 | GGT7 | Glutathione hydro | 534 | 1 | 534 | 0.026217228 | 0.026217228 | 0.026217228 |
| Q16445 | 46 | GABRA6 | Gamma-aminobut | 222 | 1 | 222 | 0.040540541 | 0.013513514 | 0.027027027 |
| P07359 | 47 | GP1BA | Platelet glycoprot | 514 | 1 | 514 | 0.134241245 | 0.145914397 | 0.140077821 |
| Q14956 | 48 | GPNMB | Transmembrane g | 475 | 1 | 475 | 0.056842105 | 0.048421053 | 0.052631579 |
| P38484 | 49 | IFNGR2 | Interferon gamma | 219 | 1 | 219 | 0.063926941 | 0.02739726 | 0.0456621 |
| P26006 | 50 | ITGA3 | Integrin alpha-3 ( | 958 | 1 | 958 | 0.030271399 | 0.017745303 | 0.024008351 |
| Q8IYV9 | 51 | IZUMO1 | Izumo sperm-egg | 270 | 1 | 270 | 0.037037037 | 0.025925926 | 0.031481481 |
| Q8N386 | 52 | LRRC25 | Leucine-rich repe | 144 | 1 | 144 | 0.034722222 | 0.027777778 | 0.03125 |
| Q8N7C0 | 53 | LRRC52 | Leucine-rich repe | 220 | 1 | 220 | 0.054545455 | 0.031818182 | 0.043181818 |
| Q01453 | 54 | PMP22 | Peripheral myelin | 45 | 2 | 32 | 0.066666667 | 0.022222222 | 0.044444444 |
| Q9NSD5 | 55 | SLC6A13 | Sodium- and chlo | 64 | 1 | 64 | 0.09375 | 0.078125 | 0.0859375 |
| Q6ZMH5 | 56 | SLC39A5 | Zinc transporter Z | 226 | 4 | 191 | 0.03539823 | 0.044247788 | 0.039823009 |
| Q9C0K1 | 57 | SLC39A8 | Metal cation symp | 128 | 4 | 109 | 0.0234375 | 0.0390625 | 0.03125 |
| O95754 | 58 | SEMA4F | Semaphorin-4F (S | 624 | 1 | 624 | 0.025641026 | 0.016025641 | 0.020833333 |
| Q12884 | 59 | FAP | Prolyl endopeptid | 734 | 1 | 734 | 0.020435967 | 0.01226158 | 0.016348774 |
| Q14524 | 60 | SCN5A | Sodium channel p | 307 | 16 | 84 | 0.104234528 | 0.042345277 | 0.073289902 |
| Q13291 | 61 | SLAMF1 | Signaling lymphoc | 216 | 1 | 216 | 0.069444444 | 0.037037037 | 0.053240741 |
| P09758 | 62 | TACSTD2 | Tumor-associatec | 247 | 1 | 247 | 0.032388664 | 0.036437247 | 0.034412955 |
| Q9UMX9 | 63 | SLC45A2 | Membrane-assoc | 52 | 5 | 26 | 0.038461538 | 0.019230769 | 0.028846154 |
| Q8WVN6 | 64 | SECTM1 | Secreted and tran | 116 | 1 | 116 | 0.034482759 | 0.00862069 | 0.021551724 |
| Q6IA17 | 65 | SIGIRR | Single Ig IL-1-rela | 117 | 1 | 117 | 0.085470085 | 0.034188034 | 0.05982906 |
| Q9NYB5 | 66 | SLCO1C1 | Solute carrier orga | 247 | 6 | 111 | 0.056680162 | 0.044534413 | 0.050607287 |
| Q9H3R2 | 67 | MUC13 | Mucin-13 (MUC-1 | 402 | 1 | 402 | 0.156716418 | 0.186567164 | 0.171641791 |
| O14931 | 68 | NCR3 | Natural cytotoxicit | 116 | 1 | 116 | 0.025862069 | 0.00862069 | 0.017241379 |
| Q9H255 | 69 | OR51E2 | Olfactory receptor | 95 | 4 | 35 | 0.042105263 | 0.010526316 | 0.026315789 |
| Q5T2D2 | 70 | TREML2 | Trem-like transcri | 249 | 1 | 249 | 0.144578313 | 0.180722892 | 0.162650602 |
| Q7Z410 | 71 | TMPRSS9 | Transmembrane p | 1008 | 1 | 1008 | 0.054563492 | 0.055555556 | 0.055059524 |
| Q9UIG8 | 72 | SLCO3A1 | Solute carrier orga | 228 | 6 | 100 | 0.065789474 | 0.043859649 | 0.054824561 |
| P30874 | 73 | SSTR2 | Somatostatin rece | 90 | 4 | 42 | 0.133333333 | 0.122222222 | 0.127777778 |
| Q9NR97 | 74 | TLR8 | Toll-like receptor | 800 | 1 | 800 | 0 | 0.03875 | 0.019375 |
| P23510 | 75 | TNFSF4 | Tumor necrosis fa | 132 | 1 | 132 | 0.03030303 | 0.03030303 | 0.03030303 |
| Q06643 | 76 | LTB | Lymphotoxin-beta | 195 | 1 | 195 | 0.035897436 | 0.020512821 | 0.028205128 |
| P18433 | 77 | PTPRA | Receptor-type tyr | 131 | 1 | 131 | 0.305343511 | 0.114503817 | 0.209923664 |
| Q96L08 | 78 | SUSD3 | Sushi domain-con | 102 | 1 | 102 | 0.049019608 | 0.058823529 | 0.053921569 |
| Q9P246 | 79 | STIM2 | Stromal interactio | 203 | 1 | 203 | 0.044334975 | 0.054187192 | 0.049261084 |
| Q9Y210 | 80 | TRPC6 | Short transient re | 124 | 3 | 49 | 0.024193548 | 0.024193548 | 0.024193548 |
| Q9NRS4 | 81 | TMPRSS4 | Transmembrane p | 383 | 1 | 383 | 0.01305483 | 0.033942559 | 0.023498695 |
| Q96QD8 | 82 | SLC38A2 | Sodium-coupled r | 96 | 6 | 53 | 0.0625 | 0.052083333 | 0.057291667 |
| P78324 | 83 | SIRPA | Tyrosine-protein p | 342 | 1 | 342 | 0.073099415 | 0.043859649 | 0.058479532 |
| Q9HCX4 | 84 | TRPC7 | Short transient re | 108 | 3 | 50 | 0.055555556 | 0.018518519 | 0.037037037 |
| Q9H3S1 | 85 | SEMA4A | Semaphorin-4A (S | 650 | 1 | 650 | 0.030769231 | 0.032307692 | 0.031538462 |
| Q15858 | 86 | SCN9A | Sodium channel p | 304 | 16 | 78 | 0.019736842 | 0.016447368 | 0.018092105 |
| Q6ZSJ9 | 87 | SHISA6 | Protein shisa-6 | 148 | 1 | 148 | 0.060810811 | 0.054054054 | 0.057432432 |
| O43613 | 88 | HCRTR1 | Orexin receptor ty | 102 | 4 | 45 | 0.039215686 | 0.049019608 | 0.044117647 |
| Q9H2H9 | 89 | SLC38A1 | Sodium-coupled r | 100 | 6 | 37 | 0.08 | 0.1 | 0.09 |
| P48066 | 90 | SLC6A11 | Sodium- and chlo | 73 | 1 | 73 | 0.082191781 | 0.04109589 | 0.061643836 |
| Q6P5W5 | 91 | SLC39A4 | Zinc transporter Z | 339 | 4 | 304 | 0.017699115 | 0.053097345 | 0.03539823 |
| Q9GZV3 | 92 | SLC5A7 | High affinity cholir | 110 | 7 | 24 | 0.045454545 | 0.027272727 | 0.036363636 |
| Q86YL7 | 93 | PDPN | Podoplanin (Aggr | 108 | 1 | 108 | 0.185185185 | 0.259259259 | 0.222222222 |
| Q9BX97 | 94 | PLVAP | Plasmalemma ves | 393 | 1 | 393 | 0.017811705 | 0.050890585 | 0.034351145 |
| Q04912 | 95 | MST1R | Macrophage-stim | 932 | 1 | 932 | 0.035407725 | 0.022532189 | 0.028969957 |
| Q8WXD0 | 96 | RXFP2 | Relaxin receptor 2 | 475 | 4 | 415 | 0.021052632 | 0.025263158 | 0.023157895 |
| Q9NY46 | 97 | SCN3A | Sodium channel p | 321 | 16 | 98 | 0.046728972 | 0.028037383 | 0.037383178 |
| Q9Y286 | 98 | SIGLEC7 | Sialic acid-binding | 334 | 1 | 334 | 0.077844311 | 0.038922156 | 0.058383234 |
| Q9H813 | 99 | PACC1 | Proton-activated | 215 | 1 | 215 | 0.027906977 | 0.027906977 | 0.027906977 |
| O14522 | 100 | PTPRT | Receptor-type tyr | 721 | 1 | 721 | 0.099861304 | 0.031900139 | 0.065880721 |
| P37173 | 101 | TGFBR2 | TGF-beta recepto | 143 | 1 | 143 | 0 | 0.027972028 | 0.013986014 |
| Q86UF1 | 102 | TSPAN33 | Tetraspanin-33 (T | 135 | 2 | 117 | 0.022222222 | 0.044444444 | 0.033333333 |
| Q8IVM8 | 103 | SLC22A9 | Solute carrier fam | 133 | 6 | 106 | 0.037593985 | 0.067669173 | 0.052631579 |
| Q9BZV2 | 104 | SLC19A3 | Thiamine transpo | 64 | 6 | 24 | 0.046875 | 0.03125 | 0.0390625 |
| Q15043 | 105 | SLC39A14 | Metal cation symp | 157 | 4 | 126 | 0.031847134 | 0.057324841 | 0.044585987 |
| P53794 | 106 | SLC5A3 | Sodium/myo-inos | 222 | 7 | 97 | 0.027027027 | 0.027027027 | 0.027027027 |
| Q9BYH1 | 107 | SEZ6L | Seizure 6-like pro | 929 | 1 | 929 | 0.060279871 | 0.092572659 | 0.076426265 |
| Q86V40 | 108 | TRABD2A | Metalloprotease T | 457 | 1 | 457 | 0.039387309 | 0.067833698 | 0.053610503 |
| P43489 | 109 | TNFRSF4 | Tumor necrosis fa | 185 | 1 | 185 | 0.059459459 | 0.140540541 | 0.1 |
| O60635 | 110 | TSPAN1 | Tetraspanin-1 (Ts | 120 | 2 | 101 | 0.05 | 0.066666667 | 0.058333333 |

| | | | | | | | | | |
|---|---|---|---|---|---|---|---|---|---|
| Q6EMK4 | 111 | VASN | Vasorin (Protein s | 551 | 1 | 551 | 0.070780399 | 0.072595281 | 0.07168784 |
| P78423 | 112 | CX3CL1 | Fractalkine (C-X3 | 316 | 1 | 316 | 0.117088608 | 0.170886076 | 0.143987342 |
| Q9Y4D7 | 113 | PLXND1 | Plexin-D1 | 1224 | 1 | 1224 | 0.04248366 | 0.030228758 | 0.036356209 |
| O60895 | 114 | RAMP2 | Receptor activity- | 102 | 1 | 102 | 0.068627451 | 0.039215686 | 0.053921569 |
| Q8TD07 | 115 | RAET1E | Retinoic acid early | 194 | 1 | 194 | 0.025773196 | 0.020618557 | 0.023195876 |
| Q13586 | 116 | STIM1 | Stromal interactio | 190 | 1 | 190 | 0.068421053 | 0.015789474 | 0.042105263 |
| Q9NYW4 | 117 | TAS2R5 | Taste receptor typ | 49 | 3 | 26 | 0.06122449 | 0.020408163 | 0.040816327 |
| Q969I6 | 118 | SLC38A4 | Sodium-coupled r | 247 | 6 | 103 | 0.020242915 | 0.056680162 | 0.038461538 |
| Q9UQD0 | 119 | SCN8A | Sodium channel p | 307 | 16 | 81 | 0.029315961 | 0.026058632 | 0.027687296 |
| P19634 | 120 | SLC9A1 | Sodium/hydrogen | 144 | 7 | 71 | 0.152777778 | 0.083333333 | 0.118055556 |
| Q9UBG0 | 121 | MRC2 | C-type mannose r | 1383 | 1 | 1383 | 0 | 0.028199566 | 0.014099783 |
| Q13224 | 122 | GRIN2B | Glutamate recepto | 700 | 2 | 530 | 0.031428571 | 0.01 | 0.020714286 |
| A6NDV4 | 123 | TMEM8B | Transmembrane p | 255 | 4 | 232 | 0.039215686 | 0.039215686 | 0.039215686 |
| Q8TE23 | 124 | TAS1R2 | Taste receptor typ | 583 | 4 | 546 | 0.032590051 | 0.022298456 | 0.027444254 |
| Q9H313 | 125 | TTYH1 | Protein tweety ho | 274 | 3 | 128 | 0.018248175 | 0.02189781 | 0.020072993 |
| P53801 | 126 | PTTG1IP | Pituitary tumor-tra | 63 | 1 | 63 | 0.095238095 | 0.047619048 | 0.071428571 |
| Q14242 | 127 | SELPLG | P-selectin glycopr | 302 | 1 | 302 | 0 | 0.254966887 | 0.127483444 |
| Q9Y5Y9 | 128 | SCN10A | Sodium channel p | 261 | 16 | 68 | 0.049808429 | 0.038314176 | 0.044061303 |
| O15389 | 129 | SIGLEC5 | Sialic acid-binding | 424 | 1 | 424 | 0.056603774 | 0.025943396 | 0.041273585 |
| O43699 | 130 | SIGLEC6 | Sialic acid-binding | 320 | 1 | 320 | 0.078125 | 0.03125 | 0.0546875 |
| P31391 | 131 | SSTR4 | Somatostatin rece | 92 | 4 | 45 | 0.065217391 | 0.086956522 | 0.076086957 |
| Q14162 | 132 | SCARF1 | Scavenger recepto | 401 | 1 | 401 | 0.032418953 | 0.067331671 | 0.049875312 |
| P23468 | 133 | PTPRD | Receptor-type tyro | 1244 | 1 | 1244 | 0.053858521 | 0.016881029 | 0.035369775 |
| Q9Y5Y6 | 134 | ST14 | Suppressor of tum | 778 | 1 | 778 | 0.028277635 | 0.023136247 | 0.025706941 |
| P41732 | 135 | TSPAN7 | Tetraspanin-7 (Ts | 115 | 2 | 100 | 0.139130435 | 0.052173913 | 0.095652174 |
| Q9H7M9 | 136 | VSIR | V-type immunoglo | 161 | 1 | 161 | 0 | 0.062111801 | 0.031055901 |
| Q9BX79 | 137 | STRA6 | Receptor for retino | 165 | 5 | 50 | 0.060606061 | 0.036363636 | 0.048484848 |
| Q96GP6 | 138 | SCARF2 | Scavenger recepto | 397 | 1 | 397 | 0.035264484 | 0.055415617 | 0.04534005 |
| Q9H2S6 | 139 | TNMD | Tenomodulin (Tel | 266 | 1 | 266 | 0.015037594 | 0.022556391 | 0.018796992 |
| Q13641 | 140 | TPBG | Trophoblast glyco | 323 | 1 | 323 | 0.055727554 | 0.049535604 | 0.052631579 |
| P17948 | 141 | FLT1 | Vascular endothe | 731 | 1 | 731 | 0.050615595 | 0.017783858 | 0.034199726 |
| P22897 | 142 | MRC1 | Macrophage man | 1370 | 1 | 1370 | 0.031386861 | 0.02189781 | 0.026642336 |
| Q7Z443 | 143 | PKD1L3 | Polycystic kidney | 957 | 7 | 673 | 0.045977011 | 0.033437827 | 0.039707419 |
| P34925 | 144 | RYK | Tyrosine-protein k | 201 | 1 | 201 | 0.054726368 | 0.024875622 | 0.039800995 |
| Q9BXP2 | 145 | SLC12A9 | Solute carrier fam | 362 | 6 | 252 | 0.038674033 | 0.038674033 | 0.038674033 |
| Q9H2J7 | 146 | SLC6A15 | Sodium-depender | 157 | 2 | 95 | 0.038216561 | 0.031847134 | 0.035031847 |
| Q9H1V8 | 147 | SLC6A17 | Sodium-depender | 223 | 6 | 104 | 0.022421525 | 0.00896861 | 0.015695067 |
| Q9H3T3 | 148 | SEMA6B | Semaphorin-6B (S | 577 | 1 | 577 | 0.027729636 | 0.029462738 | 0.028596187 |
| Q9Y6L6 | 149 | SLCO1B1 | Solute carrier orga | 246 | 6 | 111 | 0.06097561 | 0.044715447 | 0.052845528 |
| Q96PQ0 | 150 | SORCS2 | VPS10 domain-co | 1027 | 1 | 1027 | 0.030185005 | 0.025316456 | 0.02775073 |
| Q9UIB8 | 151 | CD84 | SLAM family mem | 203 | 1 | 203 | 0.147783251 | 0.019704433 | 0.083743842 |
| Q16585 | 152 | SGCB | Beta-sarcoglycan | 231 | 1 | 231 | 0 | 0.051948052 | 0.025974026 |
| Q99835 | 153 | SMO | Smoothened hom | 308 | 4 | 205 | 0.055194805 | 0.019480519 | 0.037337662 |
| P35346 | 154 | SSTR5 | Somatostatin rece | 75 | 4 | 37 | 0.12 | 0.146666667 | 0.133333333 |
| Q9P2J2 | 155 | IGSF9 | Protein turtle hom | 713 | 1 | 713 | 0.051893408 | 0.019635344 | 0.035764376 |
| P09619 | 156 | PDGFRB | Platelet-derived g | 499 | 1 | 499 | 0.04008016 | 0.026052104 | 0.033066132 |
| Q16827 | 157 | PTPRO | Receptor-type tyro | 792 | 1 | 792 | 0.074494949 | 0.036616162 | 0.055555556 |
| Q0VAQ4 | 158 | SMAGP | Small cell adhesic | 35 | 1 | 35 | 0.285714286 | 0.257142857 | 0.271428571 |
| P30518 | 159 | AVPR2 | Vasopressin V2 re | 84 | 4 | 37 | 0.071428571 | 0.119047619 | 0.095238095 |
| O75051 | 160 | PLXNA2 | Plexin-A2 (Semap | 1202 | 1 | 1202 | 0.038269551 | 0.026622296 | 0.032445923 |
| Q96G91 | 161 | P2RY11 | P2Y purinoceptor | 137 | 4 | 41 | 0.02919708 | 0.02919708 | 0.02919708 |
| Q9Y6N7 | 162 | ROBO1 | Roundabout homo | 871 | 1 | 871 | 0.043628014 | 0.013777268 | 0.028702641 |
| Q685J3 | 163 | MUC17 | Mucin-17 (MUC-1 | 4367 | 1 | 4367 | 0.423631784 | 0.440577055 | 0.43210442 |
| Q8WXI7 | 164 | MUC16 | Mucin-16 (MUC-1 | 14450 | 1 | 14450 | 0.076608997 | 0.10615917 | 0.091384083 |
| P25103 | 165 | TACR1 | Substance-P rece | 85 | 4 | 30 | 0.094117647 | 0.105882353 | 0.1 |
| P26715 | 166 | KLRC1 | NKG2-A/NKG2-B | 139 | 1 | 139 | 0.035971223 | 0.021582734 | 0.028776978 |
| P41146 | 167 | OPRL1 | Nociceptin recepto | 97 | 4 | 47 | 0.030927835 | 0.06185567 | 0.046391753 |
| Q9HD43 | 168 | PTPRH | Receptor-type tyro | 726 | 1 | 726 | 0.137741047 | 0.085399449 | 0.111570248 |
| Q9NQ40 | 169 | SLC52A3 | Solute carrier fam | 140 | 6 | 61 | 0.064285714 | 0.064285714 | 0.064285714 |
| Q07699 | 170 | SCN1B | Sodium channel s | 141 | 1 | 141 | 0.028368794 | 0.028368794 | 0.028368794 |
| Q13591 | 171 | SEMA5A | Semaphorin-5A (S | 945 | 1 | 945 | 0.043386243 | 0.068783069 | 0.056084656 |
| Q96RL6 | 172 | SIGLEC11 | Sialic acid-binding | 533 | 1 | 533 | 0.052532833 | 0.033771107 | 0.04315197 |
| Q96BD0 | 173 | SLCO4A1 | Solute carrier orga | 216 | 6 | 108 | 0.041666667 | 0.023148148 | 0.032407407 |
| Q92959 | 174 | SLCO2A1 | Solute carrier orga | 232 | 6 | 102 | 0.051724138 | 0.068965517 | 0.060344828 |
| P78380 | 175 | OLR1 | Oxidized low-dens | 215 | 1 | 215 | 0.023255814 | 0.03255814 | 0.027906977 |
| P23471 | 176 | PTPRZ1 | Receptor-type tyro | 1611 | 1 | 1611 | 0.075729361 | 0.196151459 | 0.13594041 |
| Q9NY72 | 177 | SCN3B | Sodium channel s | 136 | 1 | 136 | 0.044117647 | 0.029411765 | 0.036764706 |
| Q9NPR2 | 178 | SEMA4B | Semaphorin-4B (S | 673 | 1 | 673 | 0.037147103 | 0.046062407 | 0.041604755 |
| Q9UI33 | 179 | SCN11A | Sodium channel p | 493 | 15 | 105 | 0.016227181 | 0.012170385 | 0.014198783 |
| Q96LC7 | 180 | SIGLEC10 | Sialic acid-binding | 533 | 1 | 533 | 0.046904315 | 0.024390244 | 0.03564728 |
| Q9UKN1 | 181 | MUC12 | Mucin-12 (MUC-1 | 5363 | 1 | 5363 | 0.320343092 | 0.345329107 | 0.332836099 |
| Q16549 | 182 | PCSK7 | Proprotein conver | 525 | 1 | 525 | 0.032380952 | 0.020952381 | 0.026666667 |
| Q9Y5Y4 | 183 | PTGDR2 | Prostaglandin D2 | 107 | 4 | 41 | 0.046728972 | 0.065420561 | 0.056074766 |
| O00337 | 184 | SLC28A1 | Sodium/nucleosic | 216 | 7 | 56 | 0.018518519 | 0.009259259 | 0.013888889 |
| P18827 | 185 | SDC1 | Syndecan-1 (SYN | 231 | 1 | 231 | 0.155844156 | 0.229437229 | 0.192640693 |
| Q96PQ1 | 186 | SIGLEC12 | Sialic acid-binding | 462 | 1 | 462 | 0.073593074 | 0.025974026 | 0.04978355 |
| P51172 | 187 | SCNN1D | Amiloride-sensitiv | 422 | 1 | 422 | 0.035545024 | 0.030805687 | 0.033175355 |
| O94956 | 188 | SLCO2B1 | Solute carrier orga | 233 | 6 | 103 | 0.0472103 | 0.0472103 | 0.0472103 |
| Q9P1W8 | 189 | SIRPG | Signal-regulatory | 331 | 1 | 331 | 0.057401813 | 0.033232628 | 0.045317221 |
| P11168 | 190 | SLC2A2 | Solute carrier fam | 112 | 6 | 66 | 0.071428571 | 0.035714286 | 0.053571429 |
| Q8NCM2 | 191 | KCNH5 | Potassium voltage | 66 | 4 | 51 | 0.075757576 | 0.03030303 | 0.053030303 |
| Q9BZA8 | 192 | PCDH11Y | Protocadherin-11 | 815 | 1 | 815 | 0.045398773 | 0.017177914 | 0.031288344 |
| P25116 | 193 | F2R | Proteinase-activa | 121 | 4 | 60 | 0.05785124 | 0.049586777 | 0.053719008 |
| Q9HCK4 | 194 | ROBO2 | Roundabout homo | 837 | 1 | 837 | 0.066905615 | 0.013142174 | 0.040023895 |
| O15245 | 195 | SLC22A1 | Solute carrier fam | 139 | 6 | 106 | 0.043165468 | 0.035971223 | 0.039568345 |
| Q01959 | 196 | SLC6A3 | Sodium-depender | 76 | 1 | 76 | 0.131578947 | 0.065789474 | 0.098684211 |
| Q9C0C4 | 197 | SEMA4C | Semaphorin-4C | 642 | 1 | 642 | 0.026479751 | 0.028037383 | 0.027258567 |
| Q9BRK3 | 198 | MXRA8 | Matrix remodeling | 321 | 1 | 321 | 0.034267913 | 0.021806854 | 0.028037383 |
| Q04721 | 199 | NOTCH2 | Neurogenic locus | 1651 | 1 | 1651 | 0.025439128 | 0.044215627 | 0.034827377 |
| Q8TCU5 | 200 | GRIN3A | Glutamate recepto | 810 | 2 | 650 | 0.039506173 | 0.027160494 | 0.033333333 |
| Q6W5P4 | 201 | NPSR1 | Neuropeptide S re | 111 | 4 | 51 | 0.072072072 | 0.117117117 | 0.094594595 |
| Q68DV7 | 202 | RNF43 | E3 ubiquitin-prote | 173 | 1 | 173 | 0.011560694 | 0.011560694 | 0.011560694 |
| Q7Z2H8 | 203 | SLC36A1 | Proton-coupled ar | 101 | 6 | 31 | 0.04950495 | 0.03960396 | 0.044554455 |
| Q08357 | 204 | SLC20A2 | Sodium-depender | 75 | 7 | 26 | 0.013333333 | 0.013333333 | 0.013333333 |
| Q9NSA0 | 205 | SLC22A11 | Solute carrier fam | 138 | 6 | 110 | 0.086956522 | 0.079710145 | 0.083333333 |
| Q8WTV0 | 206 | SCARB1 | Scavenger recepto | 410 | 1 | 410 | 0.03902439 | 0.024390244 | 0.031707317 |
| Q99250 | 207 | SCN2A | Sodium channel p | 324 | 16 | 98 | 0.046296296 | 0.030864198 | 0.038580247 |
| P31641 | 208 | SLC6A6 | Sodium- and chlo | 74 | 1 | 74 | 0.054054054 | 0.108108108 | 0.081081081 |
| Q9NTN9 | 209 | SEMA4G | Semaphorin-4G | 657 | 1 | 657 | 0.0456621 | 0.04261796 | 0.04414003 |
| Q8NFY4 | 210 | SEMA6D | Semaphorin-6D | 641 | 1 | 641 | 0.03900156 | 0.028081123 | 0.033541342 |
| Q8TDX9 | 211 | PKD1L1 | Polycystic kidney | 2072 | 6 | 1747 | 0.047779923 | 0.053088803 | 0.050434363 |
| O95136 | 212 | S1PR2 | Sphingosine 1-ph | 76 | 4 | 33 | 0.052631579 | 0.078947368 | 0.065789474 |
| Q9UGH3 | 213 | SLC23A2 | Solute carrier fam | 86 | 7 | 35 | 0.069767442 | 0.023255814 | 0.046511628 |
| Q695T7 | 214 | SLC6A19 | Sodium-depender | 206 | 6 | 87 | 0.058252427 | 0.024271845 | 0.041262136 |
| Q8WUX1 | 215 | SLC38A5 | Sodium-coupled r | 106 | 6 | 43 | 0.037735849 | 0.018867925 | 0.028301887 |
| P47900 | 216 | P2RY1 | P2Y purinoceptor | 108 | 4 | 50 | 0.157407407 | 0.064814815 | 0.111111111 |
| Q13635 | 217 | PTCH1 | Protein patched h | 604 | 6 | 314 | 0.01986755 | 0.013245033 | 0.016556291 |
| Q9Y226 | 218 | SLC22A13 | Solute carrier fam | 119 | 5 | 96 | 0.033613445 | 0.025210084 | 0.029411765 |
| P46059 | 219 | SLC15A1 | Solute carrier fam | 294 | 6 | 201 | 0.047619048 | 0.017006803 | 0.032312925 |
| Q9NP91 | 220 | SLC6A20 | Sodium- and chlo | 235 | 6 | 91 | 0.029787234 | 0.012765957 | 0.021276596 |
| P43007 | 221 | SLC1A4 | Neutral amino aci | 76 | 1 | 76 | 0.065789474 | 0.157894737 | 0.111842105 |
| P30531 | 222 | SLC6A1 | Sodium- and chlo | 66 | 1 | 66 | 0.090909091 | 0.060606061 | 0.075757576 |
| O76082 | 223 | SLC22A5 | Solute carrier fam | 122 | 6 | 100 | 0.024590164 | 0.057377049 | 0.040983607 |
| Q13018 | 224 | PLA2R1 | Secretory phosph | 1376 | 1 | 1376 | 0.020348837 | 0.018895349 | 0.019622093 |

| | | | | | | | | |
|---|---|---|---|---|---|---|---|---|
| Q99808 | 225 | SLC29A1 | Equilibrative nucle | 93 | 6 | 52 | 0.032258065 | 0.043010753 | 0.037634409 |
| P40879 | 226 | SLC26A3 | Chloride anion exc | 114 | 5 | 38 | 0.052631579 | 0.035087719 | 0.043859649 |
| Q96RN1 | 227 | SLC26A8 | Testis anion trans | 116 | 6 | 34 | 0.043103448 | 0.00862069 | 0.025862069 |
| Q9Y3Q4 | 228 | HCN4 | Potassium/sodiur | 42 | 4 | 22 | 0.047619048 | 0 | 0.023809524 |
| P13747 | 229 | HLA-E | HLA class I histoc | 283 | 1 | 283 | 0.028268551 | 0.010600707 | 0.019434629 |
| Q93033 | 230 | CD101 | Immunoglobulin s | 933 | 1 | 933 | 0.0471597 | 0.022508039 | 0.034833869 |
| P10721 | 231 | KIT | Mast/stem cell gr | 498 | 1 | 498 | 0 | 0.026104418 | 0.013052209 |
| O00254 | 232 | F2RL2 | Proteinase-activa | 115 | 4 | 55 | 0.060869565 | 0.060869565 | 0.060869565 |
| Q99571 | 233 | P2RX4 | P2X purinoceptor | 283 | 1 | 283 | 0.067137809 | 0.056537102 | 0.061837456 |
| O14495 | 234 | PLPP3 | Phospholipid phos | 92 | 3 | 49 | 0.065217391 | 0 | 0.032608696 |
| Q8TDB8 | 235 | SLC2A14 | Solute carrier fam | 85 | 6 | 37 | 0.035294118 | 0.011764706 | 0.023529412 |
| P16234 | 236 | PDGFRA | Platelet-derived g | 504 | 1 | 504 | 0.045634921 | 0.01984127 | 0.032738095 |
| Q969P0 | 237 | IGSF8 | Immunoglobulin s | 551 | 1 | 551 | 0.019963702 | 0.019963702 | 0.019963702 |
| P20701 | 238 | ITGAL | Integrin alpha-L ( | 1064 | 1 | 1064 | 0.028195489 | 0.015977444 | 0.022086466 |
| P05107 | 239 | ITGB2 | Integrin beta-2 (C | 677 | 1 | 677 | 0.022156573 | 0.038404727 | 0.03028065 |
| P26010 | 240 | ITGB7 | Integrin beta-7 (G | 703 | 1 | 703 | 0.031294452 | 0.055476529 | 0.043385491 |
| P03999 | 241 | OPN1SW | Short-wave-sensi | 77 | 4 | 32 | 0.012987013 | 0.038961039 | 0.025974026 |
| Q6J4K2 | 242 | SLC8B1 | Mitochondrial sod | 134 | 7 | 68 | 0.044776119 | 0.052238806 | 0.048507463 |
| P26718 | 243 | KLRK1 | NKG2-D type II in | 143 | 1 | 143 | 0.027972028 | 0.006993007 | 0.017482517 |
| Q8IUK5 | 244 | PLXDC1 | Plexin domain-cor | 407 | 1 | 407 | 0.061425061 | 0.076167076 | 0.068796069 |
| P41143 | 245 | OPRD1 | Delta-type opioid | 90 | 4 | 46 | 0.077777778 | 0.066666667 | 0.072222222 |
| P78562 | 246 | PHEX | Phosphate-regula | 599 | 1 | 599 | 0.026711185 | 0.005008347 | 0.015859766 |
| Q9P0L9 | 247 | PKD2L1 | Polycystic kidney | 278 | 4 | 231 | 0.017985612 | 0 | 0.008992806 |
| O00592 | 248 | PODXL | Podocalyxin (GCT | 438 | 1 | 438 | 0.242009132 | 0.292237443 | 0.267123288 |
| Q9UBD6 | 249 | RHCG | Ammonium transp | 89 | 5 | 29 | 0.011235955 | 0 | 0.005617978 |
| P25105 | 250 | PTAFR | Platelet-activating | 80 | 4 | 28 | 0.0625 | 0.0375 | 0.05 |
| Q8NH64 | 251 | OR51A7 | Olfactory receptor | 96 | 4 | 35 | 0.03125 | 0.020833333 | 0.026041667 |
| Q86VZ1 | 252 | P2RY8 | P2Y purinoceptor | 70 | 4 | 27 | 0.071428571 | 0.057142857 | 0.064285714 |
| O95977 | 253 | S1PR4 | Sphingosine 1-ph | 97 | 4 | 49 | 0.041237113 | 0.06185567 | 0.051546392 |
| B4DS77 | 254 | SHISA9 | Protein shisa-9 | 125 | 1 | 125 | 0.072 | 0.032 | 0.052 |
| O43300 | 255 | LRRTM2 | Leucine-rich repe | 388 | 1 | 388 | 0.048969072 | 0.059278351 | 0.054123711 |
| Q8NGF4 | 256 | OR5AP2 | Olfactory receptor | 123 | 4 | 53 | 0.024390244 | 0.008130081 | 0.016260163 |
| Q8NGZ9 | 257 | OR2T10 | Olfactory receptor | 92 | 4 | 36 | 0.02173913 | 0.010869565 | 0.016304348 |
| Q9UN66 | 258 | PCDHB8 | Protocadherin bet | 661 | 1 | 661 | 0.039334342 | 0.004538578 | 0.02193646 |
| Q9Y5E7 | 259 | PCDHB2 | Protocadherin bet | 661 | 1 | 661 | 0.030257186 | 0.006051437 | 0.018154312 |
| Q9Y5E5 | 260 | PCDHB4 | Protocadherin bet | 661 | 1 | 661 | 0.030257186 | 0.004538578 | 0.017397882 |
| B8ZZ34 | 261 | SHISA8 | Protein shisa-8 (S | 99 | 1 | 99 | 0.070707071 | 0.03030303 | 0.050505051 |
| Q9Y267 | 262 | SLC22A14 | Solute carrier fam | 140 | 6 | 92 | 0.035714286 | 0.028571429 | 0.032142857 |
| Q8NGJ6 | 263 | OR51A4 | Olfactory receptor | 98 | 4 | 35 | 0.051020408 | 0.010204082 | 0.030612245 |
| Q8NH90 | 264 | OR5AK2 | Olfactory receptor | 100 | 4 | 41 | 0.07 | 0.02 | 0.045 |
| Q9H2Y9 | 265 | SLCO5A1 | Solute carrier orga | 251 | 6 | 129 | 0.067729084 | 0.035856574 | 0.051792829 |
| Q2VWP7 | 266 | PRTG | Protogenin (Prote | 913 | 1 | 913 | 0.048192771 | 0.028477547 | 0.038335159 |
| P47872 | 267 | SCTR | Secretin receptor | 164 | 4 | 120 | 0.012195122 | 0.036585366 | 0.024390244 |
| Q4G0T1 | 268 | 0 | Scavenger recept | 886 | 1 | 886 | 0.019187359 | 0.028216704 | 0.023702032 |
| Q8NGS8 | 269 | OR13C5 | Olfactory receptor | 101 | 4 | 36 | 0.01980198 | 0.00990099 | 0.014851485 |
| Q8NGE3 | 270 | OR10P1 | Olfactory receptor | 96 | 4 | 36 | 0.052083333 | 0.010416667 | 0.03125 |
| Q8NGT2 | 271 | OR13J1 | Olfactory receptor | 95 | 4 | 36 | 0.021052632 | 0 | 0.010526316 |
| Q5TZ20 | 272 | OR2G6 | Olfactory receptor | 92 | 4 | 36 | 0.054347826 | 0.02173913 | 0.038043478 |
| Q8NGR8 | 273 | OR1L8 | Olfactory receptor | 93 | 4 | 37 | 0.021505376 | 0.010752688 | 0.016129032 |
| Q8NGB6 | 274 | OR4M2 | Olfactory receptor | 90 | 4 | 36 | 0.044444444 | 0.011111111 | 0.027777778 |
| A3KFT3 | 275 | OR2M5 | Olfactory receptor | 92 | 4 | 36 | 0.065217391 | 0.02173913 | 0.043478261 |
| Q8NGG7 | 276 | OR8A1 | Olfactory receptor | 114 | 4 | 50 | 0.035087719 | 0.01754386 | 0.026315789 |
| Q86UG4 | 277 | SLCO6A1 | Solute carrier orga | 219 | 6 | 108 | 0.063926941 | 0.02739726 | 0.0456621 |
| P59551 | 278 | TAS2R60 | Taste receptor typ | 73 | 4 | 33 | 0.02739726 | 0.01369863 | 0.020547945 |
| A6NFU0 | 279 | FAM187A | Ig-like V-type dor | 358 | 1 | 358 | 0 | 0.016759777 | 0.008379888 |
| Q9H0X4 | 280 | FAM234A | Protein FAM234A | 481 | 1 | 481 | 0.087318087 | 0.033264033 | 0.06029106 |
| Q8TBQ9 | 281 | TMEM167A | Protein kish-A (Tr | 26 | 1 | 26 | 0.038461538 | 0.038461538 | 0.038461538 |
| Q14952 | 282 | KIR2DS3 | Killer cell immuno | 223 | 1 | 223 | 0.062780269 | 0.044843049 | 0.053811659 |
| Q86VZ4 | 283 | LRP11 | Low-density lipop | 412 | 1 | 412 | 0 | 0.060679612 | 0.030339806 |
| Q9P1P4 | 284 | TAAR3P | Putative trace am | 106 | 4 | 60 | 0.037735849 | 0.009433962 | 0.023584906 |
| P59541 | 285 | TAS2R30 | Taste receptor typ | 56 | 3 | 30 | 0.035714286 | 0.035714286 | 0.035714286 |
| Q8IW52 | 286 | SLITRK4 | SLIT and NTRK-li | 599 | 1 | 599 | 0.033388982 | 0.048414023 | 0.040901503 |
| Q8NC67 | 287 | NETO2 | Neuropilin and tol | 324 | 1 | 324 | 0.024691358 | 0.015432099 | 0.020061728 |
| Q6NVV3 | 288 | NIPAL1 | Magnesium trans | 113 | 4 | 66 | 0.053097345 | 0.044247788 | 0.048672566 |
| Q6IFG1 | 289 | OR52E8 | Olfactory receptor | 102 | 4 | 35 | 0.039215686 | 0.019607843 | 0.029411765 |
| Q8NH74 | 290 | OR10A6 | Olfactory receptor | 95 | 4 | 36 | 0.010526316 | 0.010526316 | 0.010526316 |
| Q8WZA6 | 291 | OR1E3 | Olfactory receptor | 81 | 4 | 27 | 0.049382716 | 0.012345679 | 0.030864198 |
| Q9NZP0 | 292 | OR6C3 | Olfactory receptor | 92 | 4 | 36 | 0.054347826 | 0.032608696 | 0.043478261 |
| Q8NGM9 | 293 | OR8D4 | Olfactory receptor | 95 | 4 | 36 | 0.031578947 | 0.010526316 | 0.021052632 |
| Q8NGE1 | 294 | OR6C4 | Olfactory receptor | 93 | 4 | 36 | 0.043010753 | 0.021505376 | 0.032258065 |
| O95047 | 295 | OR2A4 | Olfactory receptor | 91 | 4 | 36 | 0.021978022 | 0.010989011 | 0.016483516 |
| Q8NGT1 | 296 | OR2K2 | Olfactory receptor | 73 | 4 | 34 | 0.01369863 | 0.01369863 | 0.01369863 |
| Q9Y3N9 | 297 | OR2W1 | Olfactory receptor | 92 | 4 | 36 | 0.032608696 | 0.010869565 | 0.02173913 |
| Q7Z3T1 | 298 | OR2W3 | Olfactory receptor | 92 | 4 | 36 | 0.054347826 | 0.010869565 | 0.032608696 |
| P59534 | 299 | TAS2R39 | Taste receptor typ | 83 | 4 | 29 | 0.036144578 | 0.036144578 | 0.036144578 |
| Q9NYW6 | 300 | TAS2R3 | Taste receptor typ | 81 | 4 | 36 | 0.037037037 | 0.037037037 | 0.037037037 |
| Q7Z402 | 301 | TMC7 | Transmembrane c | 290 | 5 | 167 | 0.065517241 | 0.093103448 | 0.079310345 |
| Q9NYW3 | 302 | TAS2R7 | Taste receptor typ | 71 | 4 | 37 | 0.042253521 | 0.042253521 | 0.042253521 |
| Q7Z3Q1 | 303 | SLC46A3 | Solute carrier fam | 83 | 6 | 47 | 0.048192771 | 0.036144578 | 0.042168675 |
| Q58EX2 | 304 | SDK2 | Protein sidekick-2 | 1911 | 1 | 1911 | 0.052328624 | 0.026687598 | 0.039508111 |
| Q96RJ0 | 305 | TAAR1 | Trace amine-asso | 84 | 4 | 30 | 0.023809524 | 0.023809524 | 0.023809524 |
| Q9Y5P1 | 306 | OR51B2 | Olfactory receptor | 94 | 4 | 35 | 0.031914894 | 0 | 0.015957447 |
| Q8NGJ8 | 307 | OR51S1 | Olfactory receptor | 103 | 4 | 34 | 0.077669903 | 0.058252427 | 0.067961165 |
| Q9H228 | 308 | S1PR5 | Sphingosine 1-ph | 90 | 4 | 39 | 0.033333333 | 0.033333333 | 0.033333333 |
| A6NK97 | 309 | SLC22A20P | Solute carrier fam | 171 | 6 | 92 | 0.064327485 | 0.01754386 | 0.040935673 |
| Q6UWL2 | 310 | SUSD1 | Sushi domain-cor | 691 | 1 | 691 | 0.073806078 | 0.075253256 | 0.074529667 |
| Q3SY77 | 311 | UGT3A2 | UDP-glucuronosy | 460 | 1 | 460 | 0 | 0.006521739 | 0.00326087 |
| Q7RTR8 | 312 | TAS2R42 | Taste receptor typ | 78 | 4 | 38 | 0.038461538 | 0.012820513 | 0.025641026 |
| Q7Z5N4 | 313 | SDK1 | Protein sidekick-1 | 2008 | 1 | 2008 | 0.0687251 | 0.030378486 | 0.049551793 |
| Q8NGJ9 | 314 | OR51T1 | Olfactory receptor | 98 | 4 | 35 | 0.06122449 | 0.030612245 | 0.045918367 |
| Q9Y5H3 | 315 | PCDHGA10 | Protocadherin gar | 663 | 1 | 663 | 0.040723982 | 0.004524887 | 0.022624434 |
| Q9BSA4 | 316 | TTYH2 | Protein tweety ho | 273 | 3 | 126 | 0.080586081 | 0.021978022 | 0.051282051 |
| Q9Y6U7 | 317 | RNF215 | RING finger prote | 206 | 1 | 206 | 0.019417476 | 0.009708738 | 0.014563107 |
| Q14DG7 | 318 | TMEM132B | Transmembrane p | 902 | 1 | 902 | 0.041019956 | 0.026607539 | 0.033813747 |
| Q9C0I4 | 319 | THSD7B | Thrombospondin | 1523 | 1 | 1523 | 0.024950755 | 0.105055811 | 0.065003283 |
| Q96H15 | 320 | TIMD4 | T-cell immunoglol | 289 | 1 | 289 | 0 | 0.238754325 | 0.119377163 |
| Q6UXD5 | 321 | SEZ6L2 | Seizure 6-like pro | 816 | 1 | 816 | 0.067401961 | 0.082107843 | 0.074754902 |
| P48145 | 322 | NPBWR1 | Neuropeptides B/ | 83 | 4 | 36 | 0.072289157 | 0.108433735 | 0.090361446 |
| Q9HBY0 | 323 | NOX3 | NADPH oxidase 3 | 230 | 3 | 172 | 0.026086957 | 0.034782609 | 0.030434783 |
| A6NL88 | 324 | SHISA7 | Protein shisa-7 (C | 166 | 1 | 166 | 0.090361446 | 0.090361446 | 0.090361446 |
| P59539 | 325 | TAS2R45 | Taste receptor typ | 59 | 3 | 30 | 0.050847458 | 0.016949153 | 0.033898305 |
| P59533 | 326 | TAS2R38 | Taste receptor typ | 62 | 4 | 26 | 0.129032258 | 0.080645161 | 0.10483871 |
| P59544 | 327 | TAS2R50 | Taste receptor typ | 51 | 3 | 33 | 0.058823529 | 0.019607843 | 0.039215686 |
| P0DTE0 | 328 | TAS2R36 | Putative taste rec | 52 | 3 | 33 | 0.038461538 | 0.038461538 | 0.038461538 |
| Q9UBY0 | 329 | SLC9A2 | Sodium/hydrogen | 101 | 6 | 31 | 0.01980198 | 0.00990099 | 0.014851485 |
| Q8N3T6 | 330 | TMEM132C | Transmembrane p | 894 | 1 | 894 | 0.023489933 | 0.029082774 | 0.026286353 |
| Q86XT9 | 331 | TMEM219 | Insulin-like growtl | 165 | 1 | 165 | 0.109090909 | 0.018181818 | 0.063636364 |
| Q8NH53 | 332 | OR52N1 | Olfactory receptor | 99 | 4 | 35 | 0.03030303 | 0.01010101 | 0.02020202 |
| A6NM03 | 333 | OR2AG2 | Olfactory receptor | 97 | 4 | 44 | 0.020618557 | 0.020618557 | 0.020618557 |
| A6NMU1 | 334 | OR52A4P | Olfactory receptor | 88 | 4 | 35 | 0.034090909 | 0.011363636 | 0.022727273 |
| P30954 | 335 | OR10J1 | Olfactory receptor | 105 | 4 | 35 | 0.019047619 | 0.019047619 | 0.019047619 |
| W5XKT8 | 336 | SPACA6 | Sperm acrosome | 268 | 1 | 268 | 0.007462687 | 0.003731343 | 0.005597015 |
| Q8NH04 | 337 | OR2T27 | Olfactory receptor | 75 | 4 | 36 | 0.026666667 | 0.013333333 | 0.02 |
| Q8NGC3 | 338 | OR10G2 | Olfactory receptor | 97 | 4 | 36 | 0.041237113 | 0.010309278 | 0.025773196 |

| | | | | | | | | | |
|---|---|---|---|---|---|---|---|---|---|
| Q8NGC4 | 339 | OR10G3 | Olfactory receptor | 93 | 4 | 36 | 0.075268817 | 0.021505376 | 0.048387097 |
| Q6UWM9 | 340 | UGT2A3 | UDP-glucuronosy | 467 | 1 | 467 | 0 | 0.004282655 | 0.002141328 |
| Q8NGH8 | 341 | OR56A4 | Olfactory receptor | 99 | 4 | 35 | 0.060606061 | 0.02020202 | 0.04040404 |
| Q8NGK2 | 342 | OR52B4 | Olfactory receptor | 98 | 4 | 35 | 0.051020408 | 0.010204082 | 0.030612245 |
| Q9GZN6 | 343 | SLC6A16 | Orphan sodium- a | 134 | 2 | 91 | 0.014925373 | 0.02238806 | 0.018656716 |
| Q6AI14 | 344 | SLC9A4 | Sodium/hydrogen | 105 | 6 | 30 | 0.019047619 | 0.00952381 | 0.014285714 |
| Q9Y5F8 | 345 | PCDHGB7 | Protocadherin gar | 660 | 1 | 660 | 0.022727273 | 0.006060606 | 0.014393939 |
| Q8TDF5 | 346 | NETO1 | Neuropilin and tol | 321 | 1 | 321 | 0.018691589 | 0.021806854 | 0.020249221 |
| Q8NGH7 | 347 | OR52L1 | Olfactory receptor | 114 | 4 | 42 | 0.01754386 | 0.00877193 | 0.013157895 |
| Q9H340 | 348 | OR51B6 | Olfactory receptor | 94 | 4 | 35 | 0.042553191 | 0.010638298 | 0.026595745 |
| Q9Y5F6 | 349 | PCDHGC5 | Protocadherin gar | 663 | 1 | 663 | 0.028657617 | 0.006033183 | 0.0173454 |
| Q9Y5G3 | 350 | PCDHGB1 | Protocadherin gar | 658 | 1 | 658 | 0.031914894 | 0.009118541 | 0.020516717 |
| Q9ULK6 | 351 | RNF150 | RING finger prote | 173 | 1 | 173 | 0.057803468 | 0.028901734 | 0.043352601 |
| Q8NGI3 | 352 | OR56B1 | Olfactory receptor | 104 | 4 | 35 | 0.038461538 | 0.019230769 | 0.028846154 |
| Q8NG77 | 353 | OR2T12 | Olfactory receptor | 90 | 4 | 36 | 0.033333333 | 0.011111111 | 0.022222222 |
| Q8NGN3 | 354 | OR10G4 | Olfactory receptor | 92 | 4 | 36 | 0.065217391 | 0.010869565 | 0.038043478 |
| Q9H207 | 355 | OR10A5 | Olfactory receptor | 96 | 4 | 36 | 0.052083333 | 0.010416667 | 0.03125 |
| Q9GZK7 | 356 | OR11A1 | Olfactory receptor | 96 | 4 | 36 | 0.041666667 | 0.010416667 | 0.026041667 |
| Q8NGX9 | 357 | OR6P1 | Olfactory receptor | 95 | 4 | 36 | 0.031578947 | 0.021052632 | 0.026315789 |
| Q8NGQ2 | 358 | OR6Q1 | Olfactory receptor | 99 | 4 | 36 | 0.050505051 | 0 | 0.025252525 |
| Q8NH40 | 359 | OR6S1 | Olfactory receptor | 94 | 4 | 38 | 0.053191489 | 0.010638298 | 0.031914894 |
| Q9Y5H9 | 360 | PCDHA2 | Protocadherin alp | 674 | 1 | 674 | 0.03264095 | 0.008902077 | 0.020771513 |
| Q8N6Y1 | 361 | PCDH20 | Protocadherin-20 | 829 | 1 | 829 | 0.031363088 | 0.002412545 | 0.016887817 |
| Q9HC56 | 362 | PCDH9 | Protocadherin-9 | 790 | 1 | 790 | 0.121518987 | 0.017721519 | 0.069620253 |
| Q9Y5H2 | 363 | PCDHGA11 | Protocadherin gar | 663 | 1 | 663 | 0.031674208 | 0.007541478 | 0.019607843 |
| O60330 | 364 | PCDHGA12 | Protocadherin gar | 662 | 1 | 662 | 0.03021148 | 0.004531722 | 0.017371601 |
| Q8TDU9 | 365 | RXFP4 | Relaxin-3 recepto | 101 | 4 | 42 | 0.079207921 | 0.108910891 | 0.094059406 |
| Q63ZE4 | 366 | SLC22A10 | Solute carrier fam | 236 | 6 | 108 | 0.042372881 | 0.046610169 | 0.044491525 |
| Q99884 | 367 | SLC6A7 | Sodium-depender | 76 | 1 | 76 | 0.039473684 | 0.026315789 | 0.032894737 |
| O95502 | 368 | NPTXR | Neuronal pentraxi | 476 | 1 | 476 | 0.033613445 | 0.050420168 | 0.042016807 |
| O60779 | 369 | SLC19A2 | Thiamine transpo | 81 | 6 | 26 | 0.037037037 | 0.024691358 | 0.030864198 |
| Q8NG76 | 370 | OR2T33 | Olfactory receptor | 90 | 4 | 36 | 0.033333333 | 0.011111111 | 0.022222222 |
| Q9H341 | 371 | OR51M1 | Olfactory receptor | 85 | 4 | 39 | 0.035294118 | 0.011764706 | 0.023529412 |
| Q8NGX0 | 372 | OR11L1 | Olfactory receptor | 95 | 4 | 36 | 0.031578947 | 0.010526316 | 0.021052632 |
| O60403 | 373 | OR10H2 | Olfactory receptor | 95 | 4 | 36 | 0.021052632 | 0.010526316 | 0.015789474 |
| Q96KK4 | 374 | OR10C1 | Olfactory receptor | 94 | 4 | 36 | 0.074468085 | 0.010638298 | 0.042553191 |
| Q8NH72 | 375 | OR4C6 | Olfactory receptor | 88 | 4 | 36 | 0.045454545 | 0.011363636 | 0.028409091 |
| Q8NGI4 | 376 | OR4D11 | Olfactory receptor | 90 | 4 | 36 | 0.033333333 | 0.011111111 | 0.022222222 |
| P0C626 | 377 | OR5G3 | Olfactory receptor | 63 | 4 | 24 | 0.031746032 | 0.015873016 | 0.023809524 |
| A6NL26 | 378 | OR5B21 | Olfactory receptor | 96 | 4 | 40 | 0.052083333 | 0.020833333 | 0.036458333 |
| Q5JQS5 | 379 | OR2B11 | Olfactory receptor | 96 | 4 | 36 | 0.03125 | 0.020833333 | 0.026041667 |
| Q9UGF6 | 380 | OR5V1 | Olfactory receptor | 94 | 4 | 35 | 0.031914894 | 0.021276596 | 0.026595745 |
| Q8NGL2 | 381 | OR5L1 | Olfactory receptor | 94 | 4 | 35 | 0.031914894 | 0 | 0.015957447 |
| O76002 | 382 | OR2J2 | Olfactory receptor | 93 | 4 | 36 | 0.021505376 | 0.010752688 | 0.016129032 |
| Q8NGS1 | 383 | OR1J4 | Olfactory receptor | 92 | 4 | 36 | 0.010869565 | 0 | 0.005434783 |
| Q96RC9 | 384 | OR8B4 | Olfactory receptor | 94 | 4 | 36 | 0.063829787 | 0.010638298 | 0.037234043 |
| Q5H943 | 385 | CT83 | Kita-kyushu lung | 91 | 1 | 91 | 0.032967033 | 0.021978022 | 0.027472527 |
| Q8NGS9 | 386 | OR13C2 | Olfactory receptor | 101 | 4 | 36 | 0.01980198 | 0.00990099 | 0.014851485 |
| O14718 | 387 | RRH | Visual pigment-lik | 68 | 4 | 25 | 0.058823529 | 0.044117647 | 0.051470588 |
| Q8NH69 | 388 | OR5W2 | Olfactory receptor | 94 | 4 | 35 | 0.021276596 | 0.021276596 | 0.021276596 |
| Q8NGP8 | 389 | OR5M1 | Olfactory receptor | 94 | 4 | 35 | 0.042553191 | 0.010638298 | 0.026595745 |
| Q8WZ94 | 390 | OR5P3 | Olfactory receptor | 94 | 4 | 35 | 0.042553191 | 0.010638298 | 0.026595745 |
| Q8NH09 | 391 | OR8S1 | Olfactory receptor | 93 | 4 | 36 | 0.021505376 | 0.010752688 | 0.016129032 |
| O43749 | 392 | OR1F1 | Olfactory receptor | 93 | 4 | 37 | 0.043010753 | 0.010752688 | 0.02688172 |
| Q6IF00 | 393 | OR2T2 | Olfactory receptor | 93 | 4 | 36 | 0.053763441 | 0 | 0.02688172 |
| Q8NH51 | 394 | OR8K3 | Olfactory receptor | 95 | 4 | 36 | 0.021052632 | 0.010526316 | 0.015789474 |
| Q15620 | 395 | OR8B8 | Olfactory receptor | 95 | 4 | 36 | 0.042105263 | 0.010526316 | 0.026315789 |
| Q86WI1 | 396 | PKHD1L1 | Fibrocystin-L (Pol | 4189 | 1 | 4189 | 0.04678921 | 0.029362616 | 0.038075913 |
| Q6PXP3 | 397 | SLC2A7 | Solute carrier fam | 81 | 6 | 35 | 0.012345679 | 0.012345679 | 0.012345679 |
| Q8NH89 | 398 | OR5AK3P | Putative olfactory | 100 | 4 | 41 | 0.06 | 0.02 | 0.04 |
| Q8NGK3 | 399 | OR52K2 | Olfactory receptor | 98 | 4 | 35 | 0.06122449 | 0.010204082 | 0.035714286 |
| Q8NH37 | 400 | OR4C3 | Olfactory receptor | 88 | 4 | 36 | 0.011363636 | 0.011363636 | 0.011363636 |
| P0DN77 | 401 | OPN1MW2 | Medium-wave-se | 96 | 4 | 51 | 0.072916667 | 0.104166667 | 0.088541667 |
| Q8NGZ2 | 402 | OR14K1 | Olfactory receptor | 98 | 4 | 41 | 0.030612245 | 0.010204082 | 0.020408163 |
| Q96R67 | 403 | OR4C12 | Olfactory receptor | 88 | 4 | 36 | 0.034090909 | 0.011363636 | 0.022727273 |
| Q8NH05 | 404 | OR4Q3 | Olfactory receptor | 90 | 4 | 36 | 0.011111111 | 0.011111111 | 0.011111111 |
| Q8NGP4 | 405 | OR5M3 | Olfactory receptor | 92 | 4 | 35 | 0.043478261 | 0.010869565 | 0.027173913 |
| P0C7N1 | 406 | OR8U8 | Olfactory receptor | 116 | 4 | 60 | 0.025862069 | 0.00862069 | 0.017241379 |
| Q96R27 | 407 | OR2M4 | Olfactory receptor | 92 | 4 | 36 | 0.032608696 | 0.010869565 | 0.02173913 |
| Q8NGE9 | 408 | OR9Q2 | Olfactory receptor | 95 | 4 | 36 | 0.031578947 | 0.010526316 | 0.021052632 |
| O14581 | 409 | OR7A17 | Olfactory receptor | 95 | 4 | 36 | 0.052631579 | 0 | 0.026315789 |
| Q8NGK9 | 410 | OR5D16 | Olfactory receptor | 96 | 4 | 35 | 0.041666667 | 0.010416667 | 0.026041667 |
| Q9GZK6 | 411 | OR2J1 | Olfactory receptor | 93 | 4 | 36 | 0.021505376 | 0.010752688 | 0.016129032 |
| Q8N0Y3 | 412 | OR4N4 | Olfactory receptor | 90 | 4 | 36 | 0.066666667 | 0.011111111 | 0.038888889 |
| Q8NGS2 | 413 | OR1J2 | Olfactory receptor | 93 | 4 | 37 | 0.021505376 | 0.010752688 | 0.016129032 |
| Q8NGR3 | 414 | OR1K1 | Olfactory receptor | 94 | 4 | 37 | 0.042553191 | 0.010638298 | 0.026595745 |
| Q9Y5G7 | 415 | PCDHGA6 | Protocadherin gar | 662 | 1 | 662 | 0.036253776 | 0.001510574 | 0.018882175 |
| Q8NGK1 | 416 | OR51G1 | Olfactory receptor | 98 | 4 | 35 | 0.020408163 | 0.010204082 | 0.015306122 |
| Q8NGK0 | 417 | OR51G2 | Olfactory receptor | 101 | 4 | 35 | 0.069306931 | 0.00990099 | 0.03960396 |
| Q99075 | 418 | HBEGF | Proheparin-bindin | 140 | 1 | 140 | 0.035714286 | 0.071428571 | 0.053571429 |
| P53708 | 419 | ITGA8 | Integrin alpha-8 [ | 973 | 1 | 973 | 0.02672148 | 0.023638232 | 0.025179856 |
| Q969F8 | 420 | KISS1R | KiSS-1 receptor (I | 106 | 4 | 45 | 0.08490566 | 0.066037736 | 0.075471698 |
| Q9H1U4 | 421 | MEGF9 | Multiple epiderma | 483 | 1 | 483 | 0.169772257 | 0.188405797 | 0.179089027 |
| Q96LB2 | 422 | MRGPRX1 | Mas-related G-pro | 59 | 4 | 30 | 0 | 0.118644068 | 0.059322034 |
| Q9UKL2 | 423 | OR52A1 | Olfactory receptor | 99 | 4 | 36 | 0.04040404 | 0.01010101 | 0.025252525 |
| A6ND48 | 424 | OR14I1 | Olfactory receptor | 72 | 4 | 26 | 0.027777778 | 0.013888889 | 0.020833333 |
| Q96R09 | 425 | OR5B2 | Olfactory receptor | 90 | 4 | 36 | 0.044444444 | 0.011111111 | 0.027777778 |
| Q8WZ92 | 426 | OR5P2 | Olfactory receptor | 97 | 4 | 35 | 0.051546392 | 0.020618557 | 0.036082474 |
| Q96RD1 | 427 | OR6C1 | Olfactory receptor | 93 | 4 | 36 | 0.043010753 | 0 | 0.021505376 |
| Q8NGF7 | 428 | OR5B17 | Olfactory receptor | 92 | 4 | 35 | 0.054347826 | 0 | 0.027173913 |
| Q9GZM6 | 429 | OR8D2 | Olfactory receptor | 95 | 4 | 36 | 0.042105263 | 0.010526316 | 0.026315789 |
| A6NKK0 | 430 | OR5H1 | Olfactory receptor | 86 | 4 | 30 | 0.034883721 | 0 | 0.01744186 |
| Q8NGC2 | 431 | OR4E2 | Olfactory receptor | 90 | 4 | 36 | 0.033333333 | 0.011111111 | 0.022222222 |
| Q8NGT9 | 432 | OR2A1; | Olfactory receptor | 92 | 4 | 37 | 0.010869565 | 0.010869565 | 0.010869565 |
| P41217 | 433 | CD200 | OX-2 membrane g | 201 | 1 | 201 | 0 | 0.034825871 | 0.017412935 |
| Q99062 | 434 | CSF3R | Granulocyte color | 602 | 1 | 602 | 0.051495017 | 0.024916944 | 0.03820598 |
| P98073 | 435 | TMPRSS15 | Enteropeptidase ( | 971 | 1 | 971 | 0.056642636 | 0.042224511 | 0.049433574 |
| P49961 | 436 | ENTPD1 | Ectonucleoside tri | 440 | 1 | 440 | 0.022727273 | 0.013636364 | 0.018181818 |
| P25092 | 437 | GUCY2C | Heat-stable enter | 406 | 1 | 406 | 0.044334975 | 0.022167488 | 0.033251232 |
| Q02846 | 438 | GUCY2D | Retinal guanylyl c | 410 | 1 | 410 | 0.029268293 | 0.002439024 | 0.015853659 |
| Q9NRM0 | 439 | SLC2A9 | Solute carrier fam | 85 | 6 | 34 | 0.023529412 | 0.023529412 | 0.023529412 |
| Q86W47 | 440 | KCNMB4 | Calcium-activated | 126 | 1 | 126 | 0.111111111 | 0.015873016 | 0.063492063 |
| O14511 | 441 | NRG2 | Pro-neuregulin-2, | 293 | 1 | 293 | 0 | 0.020477816 | 0.010238908 |
| Q15743 | 442 | GPR68 | Ovarian cancer G- | 71 | 4 | 24 | 0.098591549 | 0.056338028 | 0.077464789 |
| Q8NGJ5 | 443 | OR51L1 | Olfactory receptor | 98 | 4 | 35 | 0.010204082 | 0.010204082 | 0.010204082 |
| Q9UGF7 | 444 | OR12D3 | Olfactory receptor | 93 | 4 | 36 | 0.043010753 | 0.010752688 | 0.02688172 |
| Q8NH85 | 445 | OR8U3 | Olfactory receptor | 94 | 4 | 35 | 0.063829787 | 0 | 0.031914894 |
| Q8NH93 | 446 | OR1L3 | Olfactory receptor | 92 | 4 | 37 | 0.010869565 | 0 | 0.005434783 |
| Q8NGD0 | 447 | OR4M1 | Olfactory receptor | 90 | 4 | 36 | 0.044444444 | 0.011111111 | 0.027777778 |
| Q8NGY5 | 448 | OR6N1 | Olfactory receptor | 95 | 4 | 36 | 0.021052632 | 0.010526316 | 0.015789474 |
| Q8NHB7 | 449 | OR5K1 | Olfactory receptor | 94 | 4 | 35 | 0.031914894 | 0.010638298 | 0.021276596 |
| Q8NG78 | 450 | OR8G5 | Olfactory receptor | 130 | 4 | 59 | 0.023076923 | 0.015384615 | 0.019230769 |
| Q9NQN1 | 451 | OR2S2 | Olfactory receptor | 99 | 4 | 36 | 0.02020202 | 0.01010101 | 0.015151515 |
| P47888 | 452 | OR3A3 | Olfactory receptor | 102 | 4 | 37 | 0.039215686 | 0.009803922 | 0.024509804 |

| | | | | | | | | | |
|---|---|---|---|---|---|---|---|---|---|
| Q8NGY3 | 453 | OR6K3 | Olfactory receptor | 110 | 4 | 40 | 0.163636364 | 0.036363636 | 0.1 |
| O76001 | 454 | OR2J3 | Olfactory receptor | 95 | 4 | 36 | 0.031578947 | 0.010526316 | 0.021052632 |
| Q96R30 | 455 | OR2V2 | Olfactory receptor | 93 | 4 | 36 | 0.021505376 | 0.010752688 | 0.016129032 |
| Q8NG80 | 456 | OR2L5 | Olfactory receptor | 91 | 4 | 36 | 0.054945055 | 0.021978022 | 0.038461538 |
| Q8NGQ1 | 457 | OR9G4 | Olfactory receptor | 112 | 4 | 42 | 0.035714286 | 0.017857143 | 0.026785714 |
| Q8NGX3 | 458 | OR10T2 | Olfactory receptor | 96 | 4 | 36 | 0.041666667 | 0.010416667 | 0.026041667 |
| Q93086 | 459 | P2RX5 | P2X purinoceptor | 267 | 1 | 267 | 0.02247191 | 0.011235955 | 0.016853933 |
| O43157 | 460 | PLXNB1 | Plexin-B1 (Semap | 1470 | 1 | 1470 | 0.058503401 | 0.059863946 | 0.059183673 |
| O60741 | 461 | HCN1 | Potassium/sodiur | 60 | 4 | 25 | 0.05 | 0 | 0.025 |
| P24394 | 462 | IL4R | Interleukin-4 rece | 206 | 1 | 206 | 0.04368932 | 0.029126214 | 0.036407767 |
| P05106 | 463 | ITGB3 | Integrin beta-3 (P | 691 | 1 | 691 | 0.020260492 | 0.031837916 | 0.026049204 |
| O75022 | 464 | LILRB3 | Leukocyte immun | 419 | 1 | 419 | 0.057279236 | 0.028639618 | 0.042959427 |
| P41145 | 465 | OPRK1 | Kappa-type opioic | 107 | 4 | 56 | 0.093457944 | 0.093457944 | 0.093457944 |
| P42263 | 466 | GRIA3 | Glutamate recepto | 697 | 2 | 523 | 0.032998565 | 0.015781923 | 0.024390244 |
| O75074 | 467 | LRP3 | Low-density lipop | 459 | 1 | 459 | 0 | 0.076252723 | 0.038126362 |
| Q6P9F7 | 468 | LRRC8B | Volume-regulated | 96 | 2 | 72 | 0.03125 | 0.041666667 | 0.036458333 |
| Q68DH5 | 469 | LMBRD2 | G-protein coupled | 118 | 5 | 51 | 0 | 0.06779661 | 0.033898305 |
| Q8NHL6 | 470 | LILRB1 | Leukocyte immun | 437 | 1 | 437 | 0.057208238 | 0.04576659 | 0.051487414 |
| O60449 | 471 | LY75 | Lymphocyte antig | 1638 | 1 | 1638 | 0.023809524 | 0.020757021 | 0.022283272 |
| P0DQD5 | 472 | NPY4R2 | Neuropeptide Y re | 96 | 4 | 38 | 0.0625 | 0.083333333 | 0.072916667 |
| P50391 | 473 | NPY4R | Neuropeptide Y re | 96 | 4 | 38 | 0.0625 | 0.083333333 | 0.072916667 |
| A6NCV1 | 474 | OR6C74 | Olfactory receptor | 74 | 4 | 32 | 0.040540541 | 0.013513514 | 0.027027027 |
| Q96PE5 | 475 | OPALIN | Opalin (Oligodenc | 28 | 1 | 28 | 0.392857143 | 0.285714286 | 0.339285714 |
| Q9NZP5 | 476 | OR5AC2 | Olfactory receptor | 102 | 4 | 41 | 0.049019608 | 0.009803922 | 0.029411765 |
| Q9NPG4 | 477 | PCDH12 | Protocadherin-12 | 693 | 1 | 693 | 0.033189033 | 0.017316017 | 0.025252525 |
| Q9Y5F9 | 478 | PCDHGB6 | Protocadherin gar | 660 | 1 | 660 | 0.013636364 | 0.003030303 | 0.008333333 |
| Q9Y5E3 | 479 | PCDHB6 | Protocadherin bet | 660 | 1 | 660 | 0.021212121 | 0.007575758 | 0.014393939 |
| Q96NT5 | 480 | SLC46A1 | Proton-coupled fo | 83 | 6 | 35 | 0.048192771 | 0.048192771 | 0.048192771 |
| O60245 | 481 | PCDH7 | Protocadherin-7 ( | 850 | 1 | 850 | 0.030588235 | 0.011764706 | 0.021176471 |
| P07202 | 482 | TPO | Thyroid peroxidas | 827 | 1 | 827 | 0.026602177 | 0.029020556 | 0.027811366 |
| Q6Q8B3 | 483 | CD200R1L | Cell surface glyco | 215 | 1 | 215 | 0.074418605 | 0.03255814 | 0.053488372 |
| P49286 | 484 | MTNR1B | Melatonin recepto | 93 | 4 | 41 | 0.086021505 | 0.064516129 | 0.075268817 |
| P15941 | 485 | MUC1 | Mucin-1 (MUC-1) | 1134 | 1 | 1134 | 0.242504409 | 0.254850088 | 0.248677249 |
| Q6NSJ0 | 486 | MYORG | Myogenesis-regu | 636 | 1 | 636 | 0.018867925 | 0.009433962 | 0.014150943 |
| Q92542 | 487 | NCSTN | Nicastrin | 635 | 1 | 635 | 0.048818898 | 0.040944882 | 0.04488189 |
| A6NL08 | 488 | OR6C75 | Olfactory receptor | 74 | 4 | 32 | 0.054054054 | 0.027027027 | 0.040540541 |
| Q8NGB8 | 489 | OR4F15 | Olfactory receptor | 90 | 4 | 36 | 0.022222222 | 0.011111111 | 0.016666667 |
| Q9H209 | 490 | OR10A4 | Olfactory receptor | 96 | 4 | 36 | 0.020833333 | 0.010416667 | 0.015625 |
| Q8NGN6 | 491 | OR10G7 | Olfactory receptor | 92 | 4 | 36 | 0.076086957 | 0 | 0.038043478 |
| Q8NGC7 | 492 | OR11H6 | Olfactory receptor | 113 | 4 | 42 | 0.088495575 | 0.008849558 | 0.048672566 |
| Q8NGS6 | 493 | OR13C3 | Olfactory receptor | 131 | 4 | 54 | 0.022900763 | 0.015267176 | 0.019083969 |
| Q8NGQ4 | 494 | OR10Q1 | Olfactory receptor | 100 | 4 | 37 | 0.01 | 0.01 | 0.01 |
| Q8NGL1 | 495 | OR5D18 | Olfactory receptor | 95 | 4 | 35 | 0.052631579 | 0.010526316 | 0.031578947 |
| P47887 | 496 | OR1E2 | Olfactory receptor | 92 | 4 | 37 | 0.02173913 | 0.010869565 | 0.016304348 |
| Q8NGB4 | 497 | OR4S1 | Olfactory receptor | 88 | 4 | 36 | 0.022727273 | 0.011363636 | 0.017045455 |
| Q8NGA2 | 498 | OR7A2P | Putative olfactory | 96 | 4 | 36 | 0.03125 | 0.010416667 | 0.020833333 |
| Q8NHA8 | 499 | OR1F12 | Olfactory receptor | 93 | 4 | 37 | 0.043010753 | 0.021505376 | 0.032258065 |
| A6NF89 | 500 | OR6C6 | Olfactory receptor | 79 | 4 | 36 | 0.037974684 | 0.012658228 | 0.025316456 |
| P47881 | 501 | OR3A1 | Olfactory receptor | 94 | 4 | 37 | 0.053191489 | 0.010638298 | 0.031914894 |
| Q92896 | 502 | GLG1 | Golgi apparatus p | 1115 | 1 | 1115 | 0.014349776 | 0.012556054 | 0.013452915 |
| Q14114 | 503 | LRP8 | Low-density lipop | 784 | 1 | 784 | 0 | 0.0625 | 0.03125 |
| Q05586 | 504 | GRIN1 | Glutamate recepto | 704 | 2 | 540 | 0.036931818 | 0.029829545 | 0.033380682 |
| Q96NY8 | 505 | NECTIN4 | Nectin-4 (Ig supe | 317 | 1 | 317 | 0.050473186 | 0.022082019 | 0.036277603 |
| Q9Y5S8 | 506 | NOX1 | NADPH oxidase 1 | 225 | 3 | 168 | 0.017777778 | 0.008888889 | 0.013333333 |
| Q15155 | 507 | NOMO1 | Nodal modulator 1 | 1123 | 1 | 1123 | 0.038290294 | 0.010685663 | 0.024487979 |
| Q8NGI1 | 508 | OR56B2P | Putative olfactory | 104 | 4 | 35 | 0.019230769 | 0.009615385 | 0.014423077 |
| Q92824 | 509 | PCSK5 | Proprotein conver | 1628 | 1 | 1628 | 0.032555283 | 0.049140049 | 0.040847666 |
| Q8NGN1 | 510 | OR6T1 | Olfactory receptor | 95 | 4 | 36 | 0.010526316 | 0.010526316 | 0.010526316 |
| Q8N148 | 511 | OR6V1 | Olfactory receptor | 93 | 4 | 36 | 0.021505376 | 0.010752688 | 0.016129032 |
| Q9UN73 | 512 | PCDHA6 | Protocadherin alp | 667 | 1 | 667 | 0.020989505 | 0.010494753 | 0.015742129 |
| Q9Y5G9 | 513 | PCDHGA4 | Protocadherin gar | 663 | 1 | 663 | 0.034690799 | 0.003016591 | 0.018853695 |
| Q9Y5F2 | 514 | PCDHB11 | Protocadherin bet | 663 | 1 | 663 | 0.042232278 | 0.007541478 | 0.024886878 |
| Q9Y5F1 | 515 | PCDHB12 | Protocadherin bet | 663 | 1 | 663 | 0.043740573 | 0.007541478 | 0.025641026 |
| O75325 | 516 | LRRN2 | Leucine-rich repe | 611 | 1 | 611 | 0.031096563 | 0.027823241 | 0.029459902 |
| Q9H3W5 | 517 | LRRN3 | Leucine-rich repe | 605 | 1 | 605 | 0.028099174 | 0.018181818 | 0.023140496 |
| Q9H0U3 | 518 | MAGT1 | Magnesium trans | 206 | 3 | 154 | 0.004854369 | 0.004854369 | 0.004854369 |
| Q9UEW3 | 519 | MARCO | Macrophage rece | 455 | 1 | 455 | 0.035164835 | 0.079120879 | 0.057142857 |
| O95255 | 520 | ABCC6 | ATP-binding cass | 248 | 8 | 114 | 0 | 0.008064516 | 0.004032258 |
| Q68D85 | 521 | NCR3LG1 | Natural cytotoxicit | 237 | 1 | 237 | 0.071729958 | 0.033755274 | 0.052742616 |
| P58181 | 522 | OR10A3 | Olfactory receptor | 95 | 4 | 36 | 0.010526316 | 0.010526316 | 0.010526316 |
| Q6IEV9 | 523 | OR4C11 | Olfactory receptor | 88 | 4 | 36 | 0.034090909 | 0.011363636 | 0.022727273 |
| Q8NGR5 | 524 | OR1L4 | Olfactory receptor | 94 | 4 | 37 | 0.021276596 | 0.010638298 | 0.015957447 |
| Q96R72 | 525 | OR4K3 | Olfactory receptor | 90 | 4 | 36 | 0.066666667 | 0.011111111 | 0.038888889 |
| Q8NH73 | 526 | OR4S2 | Olfactory receptor | 88 | 4 | 36 | 0.022727273 | 0.011363636 | 0.017045455 |
| Q8N146 | 527 | OR8H3 | Olfactory receptor | 95 | 4 | 36 | 0.042105263 | 0 | 0.021052632 |
| Q8NGG1 | 528 | OR8J2 | Olfactory receptor | 91 | 4 | 40 | 0.065934066 | 0 | 0.032967033 |
| Q8NH94 | 529 | OR1L1 | Olfactory receptor | 142 | 4 | 74 | 0.028169014 | 0.028169014 | 0.028169014 |
| P41586 | 530 | ADCYAP1R1 | Pituitary adenylat | 185 | 4 | 134 | 0.032432432 | 0.027027027 | 0.02972973 |
| P30559 | 531 | OXTR | Oxytocin receptor | 92 | 4 | 37 | 0.054347826 | 0.054347826 | 0.054347826 |
| P32418 | 532 | SLC8A1 | Sodium/calcium e | 74 | 6 | 38 | 0.040540541 | 0.027027027 | 0.033783784 |
| P57103 | 533 | SLC8A3 | Sodium/calcium e | 103 | 5 | 42 | 0.058252427 | 0.077669903 | 0.067961165 |
| Q9Y5E6 | 534 | PCDHB3 | Protocadherin bet | 663 | 1 | 663 | 0.033182504 | 0.006033183 | 0.019607843 |
| Q9Y5F0 | 535 | PCDHB13 | Protocadherin bet | 661 | 1 | 661 | 0.036308623 | 0.007564297 | 0.02193646 |
| Q96AM1 | 536 | MRGPRF | Mas-related G-pr | 86 | 4 | 43 | 0 | 0.034883721 | 0.01744186 |
| Q5SSG8 | 537 | MUC21 | Mucin-21 (MUC-2 | 454 | 1 | 454 | 0.579295154 | 0.588105727 | 0.583700441 |
| Q9Y5G6 | 538 | PCDHGA7 | Protocadherin gar | 663 | 1 | 663 | 0.036199095 | 0 | 0.018099548 |
| Q9Y5G5 | 539 | PCDHGA8 | Protocadherin gar | 662 | 1 | 662 | 0.03021148 | 0.00755287 | 0.018882175 |
| Q96QE2 | 540 | SLC2A13 | Proton myo-inosit | 152 | 6 | 93 | 0.052631579 | 0.032894737 | 0.042763158 |
| Q92859 | 541 | NEO1 | Neogenin (Immun | 1071 | 1 | 1071 | 0.055088702 | 0.025210084 | 0.040149393 |
| P29371 | 542 | TACR3 | Neuromedin-K rec | 136 | 4 | 83 | 0.080882353 | 0.095588235 | 0.088235294 |
| P49279 | 543 | SLC11A1 | Natural resistance | 91 | 6 | 41 | 0.032967033 | 0.021978022 | 0.027472527 |
| Q9H205 | 544 | OR2AG1 | Olfactory receptor | 92 | 4 | 36 | 0.032608696 | 0.02173913 | 0.027173913 |
| Q8NG92 | 545 | OR13H1 | Olfactory receptor | 95 | 4 | 36 | 0.021052632 | 0 | 0.010526316 |
| Q8NHC4 | 546 | OR10J5 | Olfactory receptor | 94 | 4 | 35 | 0.021276596 | 0.010638298 | 0.015957447 |
| Q8NH48 | 547 | OR5B3 | Olfactory receptor | 98 | 4 | 41 | 0.040816327 | 0.030612245 | 0.035714286 |
| Q8NGP0 | 548 | OR4C13 | Olfactory receptor | 88 | 4 | 36 | 0.056818182 | 0.022727273 | 0.039772727 |
| Q8NGD4 | 549 | OR4K1 | Olfactory receptor | 90 | 4 | 36 | 0.033333333 | 0.011111111 | 0.022222222 |
| Q15617 | 550 | OR8G1 | Olfactory receptor | 95 | 4 | 36 | 0.105263158 | 0.021052632 | 0.063157895 |
| Q8NGR4 | 551 | OR5C1 | Olfactory receptor | 98 | 4 | 35 | 0.030612245 | 0.010204082 | 0.020408163 |
| O95222 | 552 | OR6A2 | Olfactory receptor | 99 | 4 | 35 | 0.03030303 | 0.02020202 | 0.025252525 |
| O95006 | 553 | OR2F2 | Olfactory receptor | 92 | 4 | 36 | 0.02173913 | 0.010869565 | 0.016304348 |
| Q08174 | 554 | PCDH1 | Protocadherin-1 ( | 794 | 1 | 794 | 0.032745592 | 0.010075567 | 0.021410579 |
| Q9Y5E2 | 555 | PCDHB7 | Protocadherin bet | 661 | 1 | 661 | 0.037821483 | 0.012102874 | 0.024962179 |
| O43614 | 556 | HCRTR2 | Orexin receptor ty | 114 | 4 | 53 | 0.096491228 | 0.087719298 | 0.092105263 |
| O14494 | 557 | PLPP1 | Phospholipid pho | 81 | 3 | 48 | 0.012345679 | 0.012345679 | 0.012345679 |
| Q9Y6C5 | 558 | PTCH2 | Protein patched h | 656 | 5 | 313 | 0.013719512 | 0.006097561 | 0.009908537 |
| Q9H244 | 559 | P2RY12 | P2Y purinoceptor | 80 | 4 | 26 | 0.0875 | 0.075 | 0.08125 |
| P98161 | 560 | PKD1 | Polycystin-1 (PC1 | 3357 | 6 | 3050 | 0.050938338 | 0.048257373 | 0.049597855 |
| Q8N131 | 561 | TMEM123 | Porimin (Keratino | 139 | 1 | 139 | 0.410071942 | 0.467625899 | 0.438848921 |
| Q8NFJ5 | 562 | GPRC5A | Retinoic acid-indu | 77 | 4 | 32 | 0.064935065 | 0.038961039 | 0.051948052 |
| Q01973 | 563 | ROR1 | Inactive tyrosine-| 376 | 1 | 376 | 0.039893617 | 0.010638298 | 0.025265957 |
| Q9UQQ1 | 564 | NAALADL1 | Aminopeptidase N | 711 | 1 | 711 | 0.033755274 | 0.032348805 | 0.033052039 |
| P30989 | 565 | NTSR1 | Neurotensin recep | 130 | 4 | 66 | 0.092307692 | 0.046153846 | 0.069230769 |
| Q92823 | 566 | NRCAM | Neuronal cell adh | 1142 | 1 | 1142 | 0.049912434 | 0.025394046 | 0.03765324 |

| | | | | | | | | | |
|---|---|---|---|---|---|---|---|---|---|
| Q9H344 | 567 | OR51I2 | Olfactory receptor | 96 | 4 | 35 | 0.104166667 | 0 | 0.052083333 |
| Q8NGP9 | 568 | OR5AR1 | Olfactory receptor | 101 | 4 | 44 | 0.02970297 | 0.00990099 | 0.01980198 |
| P51575 | 569 | P2RX1 | P2X purinoceptor | 287 | 1 | 287 | 0.034843206 | 0.017421603 | 0.026132404 |
| Q9BXB1 | 570 | LGR4 | Leucine-rich repe | 567 | 4 | 519 | 0.031746032 | 0.028218695 | 0.029982363 |
| Q9BTN0 | 571 | LRFN3 | Leucine-rich repe | 522 | 1 | 522 | 0.053639847 | 0.04789272 | 0.050766284 |
| Q8NGK6 | 572 | OR52I1 | Olfactory receptor | 101 | 4 | 35 | 0.02970297 | 0.00990099 | 0.01980198 |
| Q6IF82 | 573 | OR4A47 | Olfactory receptor | 88 | 4 | 36 | 0.034090909 | 0.022727273 | 0.028409091 |
| Q9Y5G0 | 574 | PCDHGB5 | Protocadherin gar | 656 | 1 | 656 | 0.024390244 | 0.00152439 | 0.012957317 |
| Q96LA9 | 575 | MRGPRX4 | Mas-related G-pro | 76 | 4 | 30 | 0 | 0.065789474 | 0.032894737 |
| Q9Y5P0 | 576 | OR51B4 | Olfactory receptor | 94 | 4 | 35 | 0.021276596 | 0.010638298 | 0.015957447 |
| Q6IF36 | 577 | OR8G2P | Putative olfactory | 94 | 4 | 40 | 0.053191489 | 0.021276596 | 0.037234043 |
| Q9H1Y3 | 578 | OPN3 | Opsin-3 (Encepha | 83 | 4 | 39 | 0.060240964 | 0.060240964 | 0.060240964 |
| B2RN74 | 579 | OR11H12 | Olfactory receptor | 103 | 4 | 43 | 0.058252427 | 0.019417476 | 0.038834951 |
| Q8NGE0 | 580 | OR10AD1 | Olfactory receptor | 95 | 4 | 36 | 0.042105263 | 0.010526316 | 0.026315789 |
| Q9UGF5 | 581 | OR14J1 | Olfactory receptor | 92 | 4 | 35 | 0.043478261 | 0.02173913 | 0.032608696 |
| Q8NGL7 | 582 | OR4P4 | Olfactory receptor | 88 | 4 | 36 | 0.045454545 | 0.022727273 | 0.034090909 |
| Q8NH43 | 583 | OR4L1 | Olfactory receptor | 90 | 4 | 36 | 0.055555556 | 0.022222222 | 0.038888889 |
| Q8WZ84 | 584 | OR8D1 | Olfactory receptor | 95 | 4 | 36 | 0.031578947 | 0.021052632 | 0.026315789 |
| Q8NGP6 | 585 | OR5M8 | Olfactory receptor | 93 | 4 | 35 | 0.021505376 | 0.010752688 | 0.016129032 |
| Q8NGR2 | 586 | OR1L6 | Olfactory receptor | 130 | 4 | 61 | 0.015384615 | 0.007692308 | 0.011538462 |
| Q8NGS3 | 587 | OR1J1 | Olfactory receptor | 93 | 4 | 37 | 0.021505376 | 0 | 0.010752688 |
| Q8NH00 | 588 | OR2T4 | Olfactory receptor | 124 | 4 | 56 | 0.14516129 | 0.040322581 | 0.092741935 |
| Q8NGR6 | 589 | OR1B1 | Olfactory receptor | 80 | 4 | 35 | 0.05 | 0.025 | 0.0375 |
| Q8NGV0 | 590 | OR2Y1 | Olfactory receptor | 91 | 4 | 35 | 0.032967033 | 0.021978022 | 0.027472527 |
| Q12913 | 591 | PTPRJ | Receptor-type tyr | 939 | 1 | 939 | 0.118210863 | 0.056443024 | 0.087326944 |
| Q96P65 | 592 | QRFPR | Pyroglutamylated | 108 | 4 | 45 | 0.037037037 | 0.055555556 | 0.046296296 |
| Q9HCN3 | 593 | PGAP6 | Post-GPI attachm | 532 | 4 | 510 | 0.045112782 | 0.037593985 | 0.041353383 |
| Q15077 | 594 | P2RY6 | P2Y purinoceptor | 93 | 4 | 28 | 0.064516129 | 0.064516129 | 0.064516129 |
| P42261 | 595 | GRIA1 | Glutamate recepto | 691 | 2 | 517 | 0.024602026 | 0.013024602 | 0.018813314 |
| O60487 | 596 | MPZL2 | Myelin protein zer | 127 | 1 | 127 | 0.039370079 | 0.015748031 | 0.027559055 |
| Q8NH60 | 597 | OR52J3 | Olfactory receptor | 98 | 4 | 35 | 0.010204082 | 0.010204082 | 0.010204082 |
| Q9Y4A9 | 598 | OR10H1 | Olfactory receptor | 95 | 4 | 36 | 0.021052632 | 0.010526316 | 0.015789474 |
| Q8NGS7 | 599 | OR13C8 | Olfactory receptor | 101 | 4 | 36 | 0.02970297 | 0.00990099 | 0.01980198 |
| Q8NHC7 | 600 | OR14C36 | Olfactory receptor | 92 | 4 | 35 | 0.054347826 | 0.010869565 | 0.032608696 |
| Q8IXE1 | 601 | OR4N5 | Olfactory receptor | 90 | 4 | 36 | 0.033333333 | 0.011111111 | 0.022222222 |
| Q8NGE8 | 602 | OR4D9 | Olfactory receptor | 90 | 4 | 36 | 0.033333333 | 0 | 0.016666667 |
| Q13606 | 603 | OR5I1 | Olfactory receptor | 96 | 4 | 35 | 0.041666667 | 0.020833333 | 0.03125 |
| Q8NGB9 | 604 | OR4F6 | Olfactory receptor | 90 | 4 | 36 | 0.022222222 | 0.011111111 | 0.016666667 |
| Q8NGM8 | 605 | OR6M1 | Olfactory receptor | 92 | 4 | 35 | 0.065217391 | 0.02173913 | 0.043478261 |
| Q8NGD2 | 606 | OR4K2 | Olfactory receptor | 90 | 4 | 36 | 0.022222222 | 0.011111111 | 0.016666667 |
| Q8NGY6 | 607 | OR6N2 | Olfactory receptor | 95 | 4 | 36 | 0.010526316 | 0.010526316 | 0.010526316 |
| Q8NH49 | 608 | OR4X1 | Olfactory receptor | 88 | 4 | 36 | 0.045454545 | 0.022727273 | 0.034090909 |
| Q96R28 | 609 | OR2M2 | Olfactory receptor | 92 | 4 | 36 | 0.086956522 | 0.02173913 | 0.054347826 |
| Q9P2E7 | 610 | PCDH10 | Protocadherin-10 | 696 | 1 | 696 | 0.027298851 | 0.005747126 | 0.016522989 |
| Q13258 | 611 | PTGDR | Prostaglandin D2 | 95 | 4 | 26 | 0.042105263 | 0.031578947 | 0.036842105 |
| Q9UN67 | 612 | PCDHB10 | Protocadherin bet | 665 | 1 | 665 | 0.042105263 | 0.007518797 | 0.02481203 |
| Q9BZA7 | 613 | PCDH11X | Protocadherin-11 | 788 | 1 | 788 | 0.04822335 | 0.020304569 | 0.034263959 |
| P22888 | 614 | LHCGR | Lutropin-choriogo | 387 | 4 | 336 | 0.03875969 | 0.054263566 | 0.046511628 |
| Q8TF66 | 615 | LRRC15 | Leucine-rich repe | 516 | 1 | 516 | 0.03875969 | 0.034883721 | 0.036821705 |
| Q8NGH6 | 616 | OR52L2P | Putative olfactory | 114 | 4 | 42 | 0.00877193 | 0.00877193 | 0.00877193 |
| P23467 | 617 | PTPRB | Receptor-type tyr | 1598 | 1 | 1598 | 0.056946183 | 0.035669587 | 0.046307885 |
| P23469 | 618 | PTPRE | Receptor-type tyr | 26 | 1 | 26 | 0.384615385 | 0.384615385 | 0.384615385 |
| Q96P68 | 619 | OXGR1 | 2-oxoglutarate rec | 106 | 4 | 33 | 0 | 0.047169811 | 0.023584906 |
| Q96MS0 | 620 | ROBO3 | Roundabout homo | 870 | 1 | 870 | 0.04137931 | 0.020689655 | 0.031034483 |
| Q9UIK5 | 621 | TMEFF2 | Tomoregulin-2 (T | 279 | 1 | 279 | 0.025089606 | 0.089605735 | 0.05734767 |
| Q9UKU6 | 622 | TRHDE | Thyrotropin-relea | 962 | 1 | 962 | 0.029106029 | 0.022869023 | 0.025987526 |
| Q6UXZ0 | 623 | TMIGD1 | Transmembrane a | 190 | 1 | 190 | 0.042105263 | 0.026315789 | 0.034210526 |
| Q9H3S3 | 624 | TMPRSS5 | Transmembrane p | 386 | 1 | 386 | 0.028497409 | 0.049222798 | 0.038860104 |
| Q8N614 | 625 | TMEM156 | Transmembrane p | 185 | 1 | 185 | 0.059459459 | 0.07027027 | 0.064864865 |
| Q7Z2W7 | 626 | TRPM8 | Transient recepto | 141 | 3 | 107 | 0.021276596 | 0.007092199 | 0.014184397 |
| Q6ZN44 | 627 | UNC5A | Netrin receptor UI | 280 | 1 | 280 | 0.021428571 | 0.010714286 | 0.016071429 |
| Q9NYW2 | 628 | TAS2R8 | Taste receptor typ | 52 | 4 | 31 | 0.019230769 | 0.019230769 | 0.019230769 |
| Q9NX61 | 629 | TMEM161A | Transmembrane p | 148 | 5 | 69 | 0.006756757 | 0.006756757 | 0.006756757 |
| A2VDJ0 | 630 | TMEM131L | Transmembrane p | 828 | 1 | 828 | 0.037439614 | 0.018115942 | 0.027777778 |
| Q9NYV9 | 631 | TAS2R13 | Taste receptor typ | 55 | 4 | 34 | 0.090909091 | 0.036363636 | 0.063636364 |
| P59542 | 632 | TAS2R19 | Taste receptor typ | 53 | 3 | 32 | 0.056603774 | 0.018867925 | 0.037735849 |
| P34981 | 633 | TRHR | Thyrotropin-relea | 73 | 4 | 27 | 0.109589041 | 0.054794521 | 0.082191781 |
| Q8NBM4 | 634 | UBAC2 | Ubiquitin-associa | 71 | 2 | 55 | 0.014084507 | 0.014084507 | 0.014084507 |
| A8MXK1 | 635 | VSTM5 | V-set and transme | 118 | 1 | 118 | 0 | 0.025423729 | 0.012711864 |
| Q01974 | 636 | ROR2 | Tyrosine-protein k | 369 | 1 | 369 | 0.037940379 | 0.008130081 | 0.02303523 |
| O75762 | 637 | TRPA1 | Transient recepto | 44 | 4 | 23 | 0.090909091 | 0.045454545 | 0.068181818 |
| P59536 | 638 | TAS2R41 | Taste receptor typ | 72 | 4 | 32 | 0.013888889 | 0.013888889 | 0.013888889 |
| P02786 | 639 | TFRC | Transferrin recept | 671 | 1 | 671 | 0.029806259 | 0.023845007 | 0.026825633 |
| O75954 | 640 | TSPAN9 | Tetraspanin-9 (Ts | 116 | 2 | 96 | 0.043103448 | 0.025862069 | 0.034482759 |
| P43115 | 641 | PTGER3 | Prostaglandin E2 | 118 | 4 | 52 | 0.076271186 | 0.06779661 | 0.072033898 |
| Q9Y336 | 642 | SIGLEC9 | Sialic acid-binding | 330 | 1 | 330 | 0.084848485 | 0.033333333 | 0.059090909 |
| Q9P0V8 | 643 | SLAMF8 | SLAM family mem | 210 | 1 | 210 | 0.076190476 | 0.014285714 | 0.045238095 |
| P40200 | 644 | CD96 | T-cell surface pro | 497 | 1 | 497 | 0 | 0.084507042 | 0.042253521 |
| P59543 | 645 | TAS2R20 | Taste receptor typ | 55 | 4 | 31 | 0.072727273 | 0.054545455 | 0.063636364 |
| Q14C87 | 646 | TMEM132D | Transmembrane p | 884 | 1 | 884 | 0.023755656 | 0.037330317 | 0.030542986 |
| P0DSN6 | 647 | TAS2R33 | Putative taste rec | 58 | 3 | 32 | 0.034482759 | 0.034482759 | 0.034482759 |
| P37288 | 648 | AVPR1A | Vasopressin V1a r | 109 | 4 | 51 | 0.119266055 | 0.091743119 | 0.105504587 |
| Q9BXS4 | 649 | TMEM59 | Transmembrane p | 202 | 1 | 202 | 0.01980198 | 0.04950495 | 0.034653465 |
| P36941 | 650 | LTBR | Tumor necrosis fa | 196 | 1 | 196 | 0.045918367 | 0.076530612 | 0.06122449 |
| Q15035 | 651 | TRAM2 | Translocating cha | 68 | 4 | 31 | 0.029411765 | 0.014705882 | 0.022058824 |
| O75509 | 652 | TNFRSF21 | Tumor necrosis fa | 307 | 1 | 307 | 0.094462541 | 0.19218241 | 0.143322476 |
| Q9H0C3 | 653 | TMEM117 | Transmembrane p | 146 | 4 | 77 | 0.034246575 | 0.034246575 | 0.034246575 |
| Q9UL52 | 654 | TMPRSS11E | Transmembrane p | 382 | 1 | 382 | 0.039267016 | 0.031413613 | 0.035340314 |
| Q8NER1 | 655 | TRPV1 | Transient recepto | 77 | 3 | 58 | 0.025974026 | 0.038961039 | 0.032467532 |
| Q6NUS8 | 656 | UGT3A1 | UDP-glucuronosy | 460 | 1 | 460 | 0 | 0.004347826 | 0.002173913 |
| Q9UBN6 | 657 | TNFRSF10D | Tumor necrosis fa | 155 | 1 | 155 | 0.090322581 | 0.167741935 | 0.129032258 |
| Q9HAV5 | 658 | EDA2R | Tumor necrosis fa | 137 | 1 | 137 | 0.02919708 | 0.102189781 | 0.065693431 |
| Q9BXR5 | 659 | TLR10 | Toll-like receptor | 556 | 1 | 556 | 0.04676259 | 0.021582734 | 0.034172662 |
| Q9Y2C9 | 660 | TLR6 | Toll-like receptor | 554 | 1 | 554 | 0 | 0.023465704 | 0.011732852 |
| P36897 | 661 | TGFBR1 | TGF-beta recepto | 92 | 1 | 92 | 0 | 0.119565217 | 0.059782609 |
| Q8N0Z9 | 662 | VSIG10 | V-set and immunc | 382 | 1 | 382 | 0 | 0.036649215 | 0.018324607 |
| P48039 | 663 | MTNR1A | Melatonin recepto | 80 | 4 | 28 | 0.05 | 0.05 | 0.05 |
| Q9Y5H5 | 664 | PCDHA9 | Protocadherin alp | 667 | 1 | 667 | 0.031484258 | 0.010494753 | 0.020989505 |
| Q9ULB1 | 665 | NRXN1 | Neurexin-1 (Neur | 1370 | 1 | 1370 | 0.035766423 | 0.052554745 | 0.044160584 |
| Q8NGE2 | 666 | OR2AP1 | Olfactory receptor | 90 | 4 | 36 | 0.077777778 | 0.033333333 | 0.055555556 |
| Q9H2C5 | 667 | OR52A5 | Olfactory receptor | 99 | 4 | 36 | 0.04040404 | 0.01010101 | 0.025252525 |
| Q96GX1 | 668 | TCTN2 | Tectonic-2 | 642 | 1 | 642 | 0.046728972 | 0.031152648 | 0.03894081 |
| Q9NS62 | 669 | THSD1 | Thrombospondin | 388 | 1 | 388 | 0.06185567 | 0.059278351 | 0.06056701 |
| Q6P7N7 | 670 | TMEM81 | Transmembrane p | 195 | 1 | 195 | 0.020512821 | 0.005128205 | 0.012820513 |
| O14817 | 671 | TSPAN4 | Tetraspanin-4 (Ts | 114 | 2 | 94 | 0.052631579 | 0.035087719 | 0.043859649 |
| Q9NS68 | 672 | TNFRSF19 | Tumor necrosis fa | 140 | 1 | 140 | 0.014285714 | 0.042857143 | 0.028571429 |
| Q6ZP80 | 673 | TMEM182 | Transmembrane p | 112 | 2 | 87 | 0.0625 | 0.035714286 | 0.049107143 |
| P21754 | 674 | ZP3 | Zona pellucida sp | 364 | 1 | 364 | 0.035714286 | 0.046703297 | 0.041208791 |
| Q9UKP6 | 675 | UTS2R | Urotensin-2 recep | 97 | 4 | 53 | 0.134020619 | 0.154639175 | 0.144329897 |
| Q96IQ7 | 676 | VSIG2 | V-set and immunc | 219 | 1 | 219 | 0 | 0.02739726 | 0.01369863 |
| Q86XK7 | 677 | VSIG1 | V-set and immunc | 210 | 1 | 210 | 0 | 0.033333333 | 0.016666667 |
| Q9BXE9 | 678 | VN1R3 | Vomeronasal type | 72 | 4 | 35 | 0.027777778 | 0.027777778 | 0.027777778 |
| Q9UPY5 | 679 | SLC7A11 | Cystine/glutamate | 91 | 6 | 30 | 0.032967033 | 0.032967033 | 0.032967033 |
| Q8TCW7 | 680 | ZPLD1 | Zona pellucida-lik | 352 | 1 | 352 | 0.071022727 | 0.056818182 | 0.063920455 |

| | | | | | | | | | |
|---|---|---|---|---|---|---|---|---|---|
| P19320 | 681 | VCAM1 | Vascular cell adhe | 673 | 1 | 673 | 0.044576523 | 0.008915305 | 0.026745914 |
| Q8NGC0 | 682 | OR5AU1 | Olfactory receptor | 129 | 4 | 78 | 0.03875969 | 0.015503876 | 0.027131783 |
| Q9UN71 | 683 | PCDHGB4 | Protocadherin gar | 658 | 1 | 658 | 0.025835866 | 0.004559271 | 0.015197568 |
| Q9UBL9 | 684 | P2RX2 | P2X purinoceptor | 273 | 1 | 273 | 0.047619048 | 0.029304029 | 0.038461538 |
| Q96FE7 | 685 | PIK3IP1 | Phosphoinositide- | 146 | 1 | 146 | 0.047945205 | 0.095890411 | 0.071917808 |
| P34995 | 686 | PTGER1 | Prostaglandin E2 | 81 | 4 | 34 | 0.098765432 | 0.111111111 | 0.104938272 |
| P23470 | 687 | PTPRG | Receptor-type tyr | 716 | 1 | 716 | 0.083798883 | 0.107541899 | 0.095670391 |
| O60896 | 688 | RAMP3 | Receptor activity- | 94 | 1 | 94 | 0.085106383 | 0.042553191 | 0.063829787 |
| P07949 | 689 | RET | Proto-oncogene t | 606 | 1 | 606 | 0 | 0.028052805 | 0.014026403 |
| Q02094 | 690 | RHAG | Ammonium transp | 62 | 6 | 27 | 0.096774194 | 0.032258065 | 0.064516129 |
| A6BM72 | 691 | MEGF11 | Multiple epiderma | 828 | 1 | 828 | 0.038647343 | 0.080917874 | 0.059782609 |
| O15438 | 692 | ABCC3 | ATP-binding cass | 250 | 8 | 114 | 0 | 0.012 | 0.006 |
| Q9UHC9 | 693 | NPC1L1 | NPC1-like intrace | 823 | 7 | 271 | 0.038882139 | 0.034021871 | 0.036452005 |
| P26717 | 694 | KLRC2 | NKG2-C type II in | 137 | 1 | 137 | 0.04379562 | 0.00729927 | 0.025547445 |
| O95436 | 695 | SLC34A2 | Sodium-depender | 202 | 4 | 128 | 0.089108911 | 0.02970297 | 0.059405941 |
| Q8NH70 | 696 | OR4A16 | Olfactory receptor | 88 | 4 | 36 | 0.022727273 | 0.022727273 | 0.022727273 |
| A6NDH6 | 697 | OR5H15 | Olfactory receptor | 86 | 4 | 30 | 0.034883721 | 0.011627907 | 0.023255814 |
| P0C7T3 | 698 | OR56A5 | Olfactory receptor | 86 | 4 | 35 | 0.081395349 | 0.046511628 | 0.063953488 |
| Q9H346 | 699 | OR52D1 | Olfactory receptor | 100 | 4 | 35 | 0.06 | 0 | 0.03 |
| Q9Y5F3 | 700 | PCDHB1 | Protocadherin bet | 662 | 1 | 662 | 0.039274924 | 0.009063444 | 0.024169184 |
| P43119 | 701 | PTGIR | Prostacyclin recep | 67 | 4 | 22 | 0.059701493 | 0.044776119 | 0.052238806 |
| Q5VY43 | 702 | PEAR1 | Platelet endotheli | 734 | 1 | 734 | 0.059945504 | 0.091280654 | 0.075613079 |
| O00222 | 703 | GRM8 | Metabotropic glut | 597 | 4 | 549 | 0.018425461 | 0.020100503 | 0.019262982 |
| A6NM11 | 704 | LRRC37A2 | Leucine-rich repe | 1546 | 1 | 1546 | 0.065329884 | 0.133247089 | 0.099288486 |
| Q86UK5 | 705 | EVC2 | Limbin (Ellis-van ( | 273 | 1 | 273 | 0.043956044 | 0.021978022 | 0.032967033 |
| Q8NGJ7 | 706 | OR51A2 | Olfactory receptor | 98 | 4 | 35 | 0.051020408 | 0.010204082 | 0.030612245 |
| Q8N271 | 707 | PROM2 | Prominin-2 (PROM | 615 | 3 | 285 | 0.021138211 | 0.034146341 | 0.027642276 |
| Q92932 | 708 | PTPRN2 | Receptor-type tyr | 593 | 1 | 593 | 0.021922428 | 0.075885329 | 0.048903879 |
| Q04671 | 709 | OCA2 | P protein (Melanc | 288 | 6 | 132 | 0.03125 | 0.038194444 | 0.034722222 |
| Q8IVU1 | 710 | IGDCC3 | Immunoglobulin s | 604 | 1 | 604 | 0.048013245 | 0.01986755 | 0.033940397 |
| Q5DX21 | 711 | IGSF11 | Immunoglobulin s | 218 | 1 | 218 | 0.055045872 | 0.009174312 | 0.032110092 |
| Q8NFM7 | 712 | IL17RD | Interleukin-17 rec | 282 | 1 | 282 | 0.053191489 | 0.031914894 | 0.042553191 |
| Q14953 | 713 | KIR2DS5 | Killer cell immuno | 223 | 1 | 223 | 0.067264574 | 0.040358744 | 0.053811659 |
| Q8IZU9 | 714 | KIRREL3 | Kin of IRRE-like pr | 513 | 1 | 513 | 0.048732943 | 0.009746589 | 0.029239766 |
| O94898 | 715 | LRIG2 | Leucine-rich repe | 766 | 1 | 766 | 0.036553525 | 0.020887728 | 0.028720627 |
| A6NND4 | 716 | OR2AT4 | Olfactory receptor | 90 | 4 | 33 | 0.066666667 | 0.011111111 | 0.038888889 |
| Q9UMZ3 | 717 | PTPRQ | Phosphatidylinosi | 1911 | 1 | 1911 | 0.076923077 | 0.038199895 | 0.057561486 |
| P58182 | 718 | OR12D2 | Olfactory receptor | 93 | 4 | 36 | 0.053763441 | 0.010752688 | 0.032258065 |
| Q8NGY7 | 719 | OR10J6P | Putative olfactory | 94 | 4 | 35 | 0.053191489 | 0.021276596 | 0.037234043 |
| Q8NH79 | 720 | OR6X1 | Olfactory receptor | 93 | 4 | 36 | 0.032258065 | 0.010752688 | 0.021505376 |
| Q8NGP3 | 721 | OR5M9 | Olfactory receptor | 92 | 4 | 35 | 0.032608696 | 0.010869565 | 0.02173913 |
| Q8NGV7 | 722 | OR5H2 | Olfactory receptor | 89 | 4 | 32 | 0.02247191 | 0.011235955 | 0.016853933 |
| O95918 | 723 | OR2H2 | Olfactory receptor | 90 | 4 | 36 | 0.033333333 | 0.011111111 | 0.022222222 |
| Q96R48 | 724 | OR2A5 | Olfactory receptor | 93 | 4 | 37 | 0.053763441 | 0.010752688 | 0.032258065 |
| Q9UN70 | 725 | PCDHGC3 | Protocadherin gar | 661 | 1 | 661 | 0.045385779 | 0.012102874 | 0.028744327 |
| Q9Y5F7 | 726 | PCDHGC4 | Protocadherin gar | 662 | 1 | 662 | 0.025679758 | 0.001510574 | 0.013595166 |
| P43116 | 727 | PTGER2 | Prostaglandin E2 | 74 | 4 | 22 | 0.054054054 | 0.067567568 | 0.060810811 |
| Q15262 | 728 | PTPRK | Receptor-type tyr | 725 | 1 | 725 | 0.046896552 | 0.028965517 | 0.037931034 |
| Q9NZ94 | 729 | NLGN3 | Neuroligin-3 (Glio | 671 | 1 | 671 | 0.031296572 | 0.035767511 | 0.033532042 |
| Q12879 | 730 | GRIN2A | Glutamate recept | 702 | 2 | 532 | 0.031339031 | 0.008547009 | 0.01994302 |
| O60462 | 731 | NRP2 | Neuropilin-2 (Vas | 843 | 1 | 843 | 0.039145907 | 0.033214709 | 0.036180308 |
| Q8NGJ3 | 732 | OR52E1 | Olfactory receptor | 95 | 4 | 35 | 0.052631579 | 0.010526316 | 0.031578947 |
| Q8NGT0 | 733 | OR13C9 | Olfactory receptor | 101 | 4 | 36 | 0.059405941 | 0.00990099 | 0.034653465 |
| Q8NGC1 | 734 | OR11G2 | Olfactory receptor | 132 | 4 | 61 | 0.03030303 | 0.015151515 | 0.022727273 |
| Q8NGB2 | 735 | OR4C5 | Olfactory receptor | 111 | 4 | 45 | 0.018018018 | 0.009009009 | 0.013513514 |
| Q9NZQ7 | 736 | CD274 | Programmed cell | 219 | 1 | 219 | 0.04109589 | 0.03196347 | 0.03652968 |
| O00398 | 737 | P2RY10 | Putative P2Y purir | 95 | 4 | 38 | 0.073684211 | 0.105263158 | 0.089473684 |
| P49683 | 738 | PRLHR | Prolactin-releasin | 111 | 4 | 61 | 0.108108108 | 0.135135135 | 0.121621622 |
| P16471 | 739 | PRLR | Prolactin receptor | 209 | 1 | 209 | 0.04784689 | 0.014354067 | 0.031100478 |
| Q6ISU1 | 740 | PTCRA | Pre T-cell antigen | 122 | 1 | 122 | 0.057377049 | 0.073770492 | 0.06557377 |
| Q7Z7M0 | 741 | MEGF8 | Multiple epiderma | 2619 | 1 | 2619 | 0.039327988 | 0.063764796 | 0.051546392 |
| Q9Y5X5 | 742 | NPFFR2 | Neuropeptide FF | 210 | 4 | 146 | 0.028571429 | 0.104761905 | 0.066666667 |
| Q92982 | 743 | NINJ1 | Ninjurin-1 (Nerve | 89 | 2 | 79 | 0.06741573 | 0.06741573 | 0.06741573 |
| Q8NA29 | 744 | MFSD2A | Sodium-depender | 179 | 6 | 74 | 0.083798883 | 0.016759777 | 0.05027933 |
| P58400 | 745 | NRXN1 | Neurexin-1-beta ( | 345 | 1 | 345 | 0.063768116 | 0.089855072 | 0.076811594 |
| Q8NH02 | 746 | OR2T29 | Olfactory receptor | 96 | 4 | 36 | 0.052083333 | 0.020833333 | 0.036458333 |
| Q8NGJ2 | 747 | OR52H1 | Olfactory receptor | 104 | 4 | 35 | 0.048076923 | 0.019230769 | 0.033653846 |
| Q8NGI8 | 748 | OR5AN1 | Olfactory receptor | 101 | 4 | 41 | 0.03960396 | 0.00990099 | 0.024752475 |
| Q8NGF1 | 749 | OR52R1 | Olfactory receptor | 99 | 4 | 35 | 0.03030303 | 0.01010101 | 0.02020202 |
| P41231 | 750 | P2RY2 | P2Y purinoceptor | 85 | 4 | 31 | 0.058823529 | 0.047058824 | 0.052941176 |
| P43630 | 751 | KIR3DL2 | Killer cell immuno | 318 | 1 | 318 | 0.066037736 | 0.037735849 | 0.051886792 |
| Q9NPC1 | 752 | LTB4R2 | Leukotriene B4 re | 89 | 4 | 29 | 0.033707865 | 0.033707865 | 0.033707865 |
| Q9Y561 | 753 | LRP12 | Low-density lipop | 459 | 1 | 459 | 0 | 0.061002179 | 0.030501089 |
| O75121 | 754 | MFAP3L | Microfibrillar-assc | 120 | 1 | 120 | 0.083333333 | 0.041666667 | 0.0625 |
| Q5SR56 | 755 | MFSD14B | Hippocampus abu | 200 | 7 | 56 | 0.035 | 0.03 | 0.0325 |
| P21757 | 756 | MSR1 | Macrophage scav | 374 | 1 | 374 | 0.040106952 | 0.06684492 | 0.053475936 |
| Q9H342 | 757 | OR51J1 | Olfactory receptor | 68 | 4 | 30 | 0.029411765 | 0.014705882 | 0.022058824 |
| Q86XR5 | 758 | PRIMA1 | Proline-rich memb | 56 | 1 | 56 | 0.071428571 | 0.107142857 | 0.089285714 |
| P10586 | 759 | PTPRF | Receptor-type tyr | 1233 | 1 | 1233 | 0.052716951 | 0.01784266 | 0.035279805 |
| Q3MIW9 | 760 | MUCL3 | Mucin-like protein | 418 | 1 | 418 | 0.377990431 | 0.301435407 | 0.339712919 |
| Q01726 | 761 | MC1R | Melanocyte-stimu | 79 | 4 | 36 | 0.037974684 | 0.012658228 | 0.025316456 |
| P13591 | 762 | NCAM1 | Neural cell adhesi | 698 | 1 | 698 | 0.042979943 | 0.018624642 | 0.030802292 |
| Q9HB89 | 763 | NMUR1 | Neuromedin-U rec | 137 | 4 | 64 | 0.02919708 | 0.02919708 | 0.02919708 |
| Q8NH61 | 764 | OR51F2 | Olfactory receptor | 110 | 4 | 38 | 0.045454545 | 0.009090909 | 0.027272727 |
| Q6IEY1 | 765 | OR4F3; | Olfactory receptor | 90 | 4 | 36 | 0.011111111 | 0.011111111 | 0.011111111 |
| P04001 | 766 | OPN1MW | Medium-wave-se | 96 | 4 | 51 | 0.072916667 | 0.104166667 | 0.088541667 |
| Q8NGC6 | 767 | OR4K17 | Olfactory receptor | 93 | 4 | 36 | 0.032258065 | 0.021505376 | 0.02688172 |
| Q8NGW6 | 768 | OR6K6 | Olfactory receptor | 122 | 4 | 52 | 0.032786885 | 0.008196721 | 0.020491803 |
| Q8NH18 | 769 | OR5J2 | Olfactory receptor | 94 | 4 | 35 | 0.031914894 | 0.010638298 | 0.021276596 |
| Q9NZP2 | 770 | OR6C2 | Olfactory receptor | 93 | 4 | 36 | 0.032258065 | 0.021505376 | 0.02688172 |
| P34982 | 771 | OR1D2 | Olfactory receptor | 92 | 4 | 37 | 0.02173913 | 0.02173913 | 0.02173913 |
| P59922 | 772 | OR2B8P | Putative olfactory | 92 | 4 | 36 | 0.02173913 | 0.032608696 | 0.027173913 |
| Q6IEU7 | 773 | OR5M10 | Olfactory receptor | 94 | 4 | 35 | 0.042553191 | 0.010638298 | 0.026595745 |
| O76099 | 774 | OR7C1 | Olfactory receptor | 95 | 4 | 36 | 0.052631579 | 0.010526316 | 0.031578947 |
| Q8NGZ5 | 775 | OR2G2 | Olfactory receptor | 95 | 4 | 36 | 0.021052632 | 0.010526316 | 0.015789474 |
| Q8NH87 | 776 | OR9G1 | Olfactory receptor | 94 | 4 | 36 | 0.031914894 | 0.010638298 | 0.021276596 |
| Q96QZ0 | 777 | PANX3 | Pannexin-3 | 82 | 2 | 52 | 0.048780488 | 0 | 0.024390244 |
| Q99572 | 778 | P2RX7 | P2X purinoceptor | 287 | 1 | 287 | 0.027874564 | 0.038327526 | 0.033101045 |
| P16284 | 779 | PECAM1 | Platelet endotheli | 573 | 1 | 573 | 0.040139616 | 0.017452007 | 0.028795812 |
| Q5FWE3 | 780 | PRRT3 | Proline-rich transr | 490 | 4 | 446 | 0.065306122 | 0.118367347 | 0.091836735 |
| Q13332 | 781 | PTPRS | Receptor-type tyr | 1252 | 1 | 1252 | 0.03913738 | 0.015175719 | 0.02715655 |
| Q92729 | 782 | PTPRU | Receptor-type tyr | 730 | 1 | 730 | 0.042465753 | 0.020547945 | 0.031506849 |
| P48357 | 783 | LEPR | Leptin receptor (L | 817 | 1 | 817 | 0.045287638 | 0.035495716 | 0.040391677 |
| Q9UJQ1 | 784 | LAMP5 | Lysosome-associ | 205 | 1 | 205 | 0.053658537 | 0.048780488 | 0.051219512 |
| A6NHG9 | 785 | OR5H14 | Olfactory receptor | 86 | 4 | 30 | 0.034883721 | 0.011627907 | 0.023255814 |
| O95013 | 786 | OR4F21 | Olfactory receptor | 90 | 4 | 36 | 0.011111111 | 0.011111111 | 0.011111111 |
| Q8NH67 | 787 | OR52I2 | Olfactory receptor | 127 | 4 | 54 | 0.023622047 | 0.015748031 | 0.019685039 |
| Q8NH63 | 788 | OR51H1 | Olfactory receptor | 99 | 4 | 35 | 0.04040404 | 0.01010101 | 0.025252525 |
| Q6P1J6 | 789 | PLB1 | Phospholipase B1 | 1395 | 1 | 1395 | 0.028673835 | 0.034408602 | 0.031541219 |
| Q969N4 | 790 | TAAR8 | Trace amine-asso | 79 | 3 | 30 | 0.050632911 | 0.063291139 | 0.056962025 |
| Q86VY9 | 791 | TMEM200A | Transmembrane p | 43 | 1 | 43 | 0.023255814 | 0.139534884 | 0.081395349 |
| Q96RI8 | 792 | TAAR6 | Trace amine-asso | 86 | 4 | 33 | 0.046511628 | 0.069767442 | 0.058139535 |
| P57727 | 793 | TMPRSS3 | Transmembrane p | 384 | 1 | 384 | 0.0078125 | 0.0078125 | 0.0078125 |
| P35408 | 794 | PTGER4 | Prostaglandin E2 | 73 | 4 | 23 | 0.095890411 | 0.082191781 | 0.089041096 |

| | | | | | | | | | |
|---|---|---|---|---|---|---|---|---|---|
| O00241 | 795 | SIRPB1 | Signal-regulatory | 341 | 1 | 341 | 0.052785924 | 0.032258065 | 0.042521994 |
| O43291 | 796 | SPINT2 | Kunitz-type prote | 169 | 1 | 169 | 0.053254438 | 0.076923077 | 0.065088757 |
| Q9NYW5 | 797 | TAS2R4 | Taste receptor typ | 50 | 4 | 19 | 0.12 | 0.04 | 0.08 |
| Q9NYW1 | 798 | TAS2R9 | Taste receptor typ | 57 | 4 | 33 | 0.035087719 | 0.01754386 | 0.026315789 |
| P13726 | 799 | F3 | Tissue factor (TF) | 218 | 1 | 218 | 0.064220183 | 0.018348624 | 0.041284404 |
| Q02763 | 800 | TEK | Angiopoietin-1 rec | 725 | 1 | 725 | 0.026206897 | 0.024827586 | 0.025517241 |
| P40238 | 801 | MPL | Thrombopoietin re | 465 | 1 | 465 | 0.047311828 | 0.03655914 | 0.041935484 |
| P16473 | 802 | TSHR | Thyrotropin recep | 441 | 4 | 392 | 0.036281179 | 0.031746032 | 0.034013605 |
| P30530 | 803 | AXL | Tyrosine-protein k | 425 | 1 | 425 | 0.056470588 | 0.030588235 | 0.043529412 |
| Q8NGJ4 | 804 | OR52E2 | Olfactory receptor | 98 | 4 | 35 | 0.020408163 | 0.010204082 | 0.015306122 |
| Q96QU1 | 805 | PCDH15 | Protocadherin-15 | 1349 | 1 | 1349 | 0.034099333 | 0.010378058 | 0.022238695 |
| Q9Y5H1 | 806 | PCDHGA2 | Protocadherin gar | 663 | 1 | 663 | 0.034690799 | 0.006033183 | 0.020361991 |
| Q8TAB3 | 807 | PCDH19 | Protocadherin-19 | 656 | 1 | 656 | 0.030487805 | 0.010670732 | 0.020579268 |
| Q9H158 | 808 | PCDHAC1 | Protocadherin alp | 664 | 1 | 664 | 0.028614458 | 0.009036145 | 0.018825301 |
| Q6V1P9 | 809 | DCHS2 | Protocadherin-23 | 2918 | 1 | 2918 | 0.037354352 | 0.007882111 | 0.022618232 |
| Q9UN72 | 810 | PCDHA7 | Protocadherin alp | 667 | 1 | 667 | 0.062968516 | 0.008995502 | 0.035982009 |
| Q9H1Z9 | 811 | TSPAN10 | Tetraspanin-10 (T | 199 | 2 | 179 | 0.020100503 | 0.025125628 | 0.022613065 |
| Q9BY10 | 812 | SLC46A2 | Thymic stromal co | 76 | 6 | 33 | 0.052631579 | 0.092105263 | 0.072368421 |
| Q6UXZ4 | 813 | UNC5D | Netrin receptor UI | 346 | 1 | 346 | 0.014450867 | 0.034682081 | 0.024566474 |
| Q9NS40 | 814 | KCNH7 | Potassium voltage | 68 | 4 | 43 | 0.014705882 | 0.014705882 | 0.014705882 |
| P22001 | 815 | KCNA3 | Potassium voltage | 93 | 3 | 40 | 0.075268817 | 0.010752688 | 0.043010753 |
| Q8IWT6 | 816 | LRRC8A | Volume-regulated | 104 | 2 | 75 | 0.057692308 | 0.019230769 | 0.038461538 |
| Q6PJG9 | 817 | LRFN4 | Leucine-rich repe | 501 | 1 | 501 | 0.045908184 | 0.031936128 | 0.038922156 |
| P0C6S8 | 818 | LINGO3 | Leucine-rich repe | 506 | 1 | 506 | 0.04743083 | 0.045454545 | 0.046442688 |
| Q12866 | 819 | MERTK | Tyrosine-protein k | 484 | 1 | 484 | 0.078512397 | 0.05785124 | 0.068181818 |
| Q8TCW9 | 820 | PROKR1 | Prokineticin recep | 135 | 4 | 61 | 0.081481481 | 0.081481481 | 0.081481481 |
| Q12836 | 821 | ZP4 | Zona pellucida sp | 486 | 1 | 486 | 0.051440329 | 0.028806584 | 0.040123457 |
| Q8NGH9 | 822 | OR52E4 | Olfactory receptor | 98 | 4 | 35 | 0.040816327 | 0.010204082 | 0.025510204 |
| Q8NGX2 | 823 | OR2T35 | Olfactory receptor | 93 | 4 | 36 | 0.053763441 | 0.010752688 | 0.032258065 |
| Q6U736 | 824 | OPN5 | Opsin-5 (G-protei | 83 | 4 | 32 | 0.024096386 | 0.024096386 | 0.024096386 |
| Q5JRS4 | 825 | OR10J3 | Olfactory receptor | 96 | 4 | 36 | 0.020833333 | 0.010416667 | 0.015625 |
| Q8NGN0 | 826 | OR4D5 | Olfactory receptor | 90 | 4 | 36 | 0.033333333 | 0.022222222 | 0.027777778 |
| Q96R08 | 827 | OR5B12 | Olfactory receptor | 92 | 4 | 35 | 0.054347826 | 0.02173913 | 0.038043478 |
| P0C645 | 828 | OR4E1 | Olfactory receptor | 103 | 4 | 43 | 0.058252427 | 0.009708738 | 0.033980583 |
| P47890 | 829 | OR1G1 | Olfactory receptor | 93 | 4 | 37 | 0.021505376 | 0.010752688 | 0.016129032 |
| O76100 | 830 | OR7A10 | Olfactory receptor | 95 | 4 | 36 | 0.052631579 | 0 | 0.026315789 |
| A6NMS3 | 831 | OR5K4 | Olfactory receptor | 95 | 4 | 44 | 0.021052632 | 0.010526316 | 0.015789474 |
| Q96RD0 | 832 | OR8B2 | Olfactory receptor | 94 | 4 | 36 | 0.021276596 | 0 | 0.010638298 |
| Q8NGW1 | 833 | OR6B3 | Olfactory receptor | 94 | 4 | 35 | 0.042553191 | 0.010638298 | 0.026595745 |
| Q8NH50 | 834 | OR8K5 | Olfactory receptor | 95 | 4 | 36 | 0.042105263 | 0.021052632 | 0.031578947 |
| Q8NGT5 | 835 | OR9A2 | Olfactory receptor | 94 | 4 | 36 | 0.031914894 | 0.010638298 | 0.021276596 |
| Q9GZK3 | 836 | OR2B2 | Olfactory receptor | 92 | 4 | 36 | 0.032608696 | 0.010869565 | 0.02173913 |
| Q8N349 | 837 | OR2L13 | Olfactory receptor | 91 | 4 | 36 | 0.021978022 | 0.021978022 | 0.021978022 |
| Q9Y5H8 | 838 | PCDHA3 | Protocadherin alp | 667 | 1 | 667 | 0.047976012 | 0.008995502 | 0.028485757 |
| Q9Y5H0 | 839 | PCDHGA3 | Protocadherin gar | 662 | 1 | 662 | 0.033232628 | 0.006042296 | 0.019637462 |
| Q9Y5I3 | 840 | PCDHA1 | Protocadherin alp | 667 | 1 | 667 | 0.026986507 | 0.010494753 | 0.01874063 |
| O14894 | 841 | TM4SF5 | Transmembrane 4 | 60 | 2 | 45 | 0.033333333 | 0.033333333 | 0.033333333 |
| P59538 | 842 | TAS2R31 | Taste receptor typ | 65 | 4 | 33 | 0.076923077 | 0.015384615 | 0.046153846 |
| Q8N3G9 | 843 | TMEM130 | Transmembrane p | 314 | 1 | 314 | 0.210191083 | 0.022292994 | 0.116242038 |
| Q12999 | 844 | TSPAN31 | Tetraspanin-31 (T | 89 | 2 | 79 | 0.08988764 | 0.011235955 | 0.050561798 |
| Q7L0X0 | 845 | TRIL | TLR4 interactor w | 670 | 1 | 670 | 0.034328358 | 0.040298507 | 0.037313433 |
| Q7Z5M5 | 846 | TMC3 | Transmembrane c | 182 | 5 | 72 | 0.010989011 | 0.016483516 | 0.013736264 |
| O95150 | 847 | TNFSF15 | Tumor necrosis fa | 194 | 1 | 194 | 0.046391753 | 0.015463918 | 0.030927835 |
| Q8NG11 | 848 | TSPAN14 | Tetraspanin-14 (T | 140 | 2 | 118 | 0.007142857 | 0.028571429 | 0.017857143 |
| Q8N9W7 | 849 | 0 | Putative transmer | 100 | 1 | 100 | 0.09 | 0.04 | 0.065 |
| P35590 | 850 | TIE1 | Tyrosine-protein k | 737 | 1 | 737 | 0.029850746 | 0.03256445 | 0.031207598 |
| Q96DZ7 | 851 | TM4SF19 | Transmembrane 4 | 81 | 2 | 60 | 0.024691358 | 0 | 0.012345679 |
| P30408 | 852 | TM4SF1 | Transmembrane 4 | 64 | 2 | 46 | 0.09375 | 0.046875 | 0.0703125 |
| Q9P1P5 | 853 | TAAR2 | Trace amine-asso | 101 | 4 | 47 | 0.03960396 | 0.079207921 | 0.059405941 |
| O43193 | 854 | MLNR | Motilin receptor (( | 131 | 4 | 67 | 0.06870229 | 0.053435115 | 0.061068702 |
| A6NIJ9 | 855 | OR6C70 | Olfactory receptor | 75 | 4 | 31 | 0.026666667 | 0 | 0.013333333 |
| Q8N127 | 856 | OR5AS1 | Olfactory receptor | 100 | 4 | 41 | 0.05 | 0.01 | 0.03 |
| Q8NGS5 | 857 | OR13C4 | Olfactory receptor | 101 | 4 | 36 | 0.01980198 | 0.00990099 | 0.014851485 |
| Q8NHC5 | 858 | OR14A16 | Olfactory receptor | 98 | 4 | 41 | 0.040816327 | 0.020408163 | 0.030612245 |
| Q8NGI7 | 859 | OR10V1 | Olfactory receptor | 96 | 4 | 36 | 0.052083333 | 0.010416667 | 0.03125 |
| P01833 | 860 | PIGR | Polymeric immunc | 619 | 1 | 619 | 0.030694669 | 0.016155089 | 0.023424879 |
| Q01118 | 861 | SCN7A | Sodium channel p | 298 | 16 | 78 | 0.026845638 | 0.026845638 | 0.026845638 |
| Q96DU3 | 862 | SLAMF6 | SLAM family mem | 204 | 1 | 204 | 0.058823529 | 0.039215686 | 0.049019608 |
| Q9NQ25 | 863 | SLAMF7 | SLAM family mem | 203 | 1 | 203 | 0.0591133 | 0.02955665 | 0.044334975 |
| Q5VX71 | 864 | SUSD4 | Sushi domain-cor | 277 | 1 | 277 | 0.025270758 | 0.036101083 | 0.030685921 |
| Q7L1I2 | 865 | SV2B | Synaptic vesicle g | 168 | 6 | 123 | 0.053571429 | 0.005952381 | 0.029761905 |
| Q6UX71 | 866 | PLXDC2 | Plexin domain-cor | 423 | 1 | 423 | 0.070921986 | 0.075650118 | 0.073286052 |
| Q16348 | 867 | SLC15A2 | Solute carrier fam | 264 | 6 | 209 | 0.037878788 | 0.03030303 | 0.034090909 |
| Q9NY91 | 868 | SLC5A4 | Solute carrier fam | 237 | 5 | 88 | 0.033755274 | 0.021097046 | 0.02742616 |
| Q8IWT1 | 869 | SCN4B | Sodium channel s | 131 | 1 | 131 | 0.030534351 | 0.022900763 | 0.026717557 |
| Q6ZMJ2 | 870 | SCARA5 | Scavenger recept | 413 | 1 | 413 | 0.024213075 | 0.050847458 | 0.037530266 |
| Q9BZG2 | 871 | ACP4 | Testicular acid ph | 366 | 1 | 366 | 0.021857923 | 0.021857923 | 0.021857923 |
| Q5R3F8 | 872 | ELFN2 | Protein phosphata | 374 | 1 | 374 | 0.064171123 | 0.069518717 | 0.06684492 |
| Q03431 | 873 | PTH1R | Parathyroid hormc | 232 | 4 | 161 | 0.034482759 | 0.068965517 | 0.051724138 |
| Q5TEA6 | 874 | SEL1L2 | Protein sel-1 hom | 638 | 1 | 638 | 0.014106583 | 0.010971787 | 0.012539185 |
| P13866 | 875 | SLC5A1 | Sodium/glucose c | 261 | 8 | 79 | 0.072796935 | 0.045977011 | 0.059386973 |
| P31645 | 876 | SLC6A4 | Sodium-depender | 158 | 6 | 65 | 0.044303797 | 0.044303797 | 0.044303797 |
| Q9NXL6 | 877 | SIDT1 | SID1 transmembra | 365 | 6 | 289 | 0.035616438 | 0.032876712 | 0.034246575 |
| Q13308 | 878 | PTK7 | Inactive tyrosine-p | 673 | 1 | 673 | 0.041604755 | 0.017830609 | 0.029717682 |
| O60939 | 879 | SCN2B | Sodium channel s | 129 | 1 | 129 | 0.03875969 | 0.031007752 | 0.034883721 |
| Q08ET2 | 880 | SIGLEC14 | Sialic acid-binding | 341 | 1 | 341 | 0.055718475 | 0.023460411 | 0.039589443 |
| Q9NPD5 | 881 | SLCO1B3 | Solute carrier orga | 246 | 6 | 111 | 0.113821138 | 0.044715447 | 0.079268293 |
| Q96A28 | 882 | SLAMF9 | SLAM family mem | 217 | 1 | 217 | 0.041474654 | 0.00921659 | 0.025345622 |
| Q13326 | 883 | SGCG | Gamma-sarcoglyc | 232 | 1 | 232 | 0.025862069 | 0.012931034 | 0.019396552 |
| Q496J9 | 884 | SV2C | Synaptic vesicle g | 164 | 5 | 119 | 0.06097561 | 0.018292683 | 0.039634146 |
| Q7L0J3 | 885 | SV2A | Synaptic vesicle g | 174 | 6 | 129 | 0.040229885 | 0.011494253 | 0.025862069 |
| P60508 | 886 | ERVFRD-1 | Syncytin-2 (Endog | 462 | 1 | 462 | 0.073593074 | 0.043290043 | 0.058441558 |
| P48230 | 887 | TM4SF4 | Transmembrane 4 | 57 | 2 | 43 | 0.035087719 | 0.035087719 | 0.035087719 |
| O14804 | 888 | TAAR5 | Trace amine-asso | 87 | 4 | 33 | 0.011494253 | 0.022988506 | 0.017241379 |
| Q8IYL9 | 889 | GPR65 | Psychosine recep | 84 | 4 | 29 | 0.047619048 | 0.035714286 | 0.041666667 |
| Q4U2R8 | 890 | SLC22A6 | Solute carrier fam | 133 | 6 | 104 | 0.097744361 | 0.060150376 | 0.078947368 |
| Q8N695 | 891 | SLC5A8 | Sodium-coupled r | 149 | 7 | 57 | 0.067114094 | 0.046979866 | 0.05704698 |
| Q92854 | 892 | SEMA4D | Semaphorin-4D (/ | 712 | 1 | 712 | 0.049157303 | 0.050561798 | 0.049859551 |
| Q9P283 | 893 | SEMA5B | Semaphorin-5B | 1035 | 1 | 1035 | 0.030917874 | 0.083091787 | 0.057004831 |
| Q53EL9 | 894 | SEZ6 | Seizure protein 6 | 905 | 1 | 905 | 0.077348066 | 0.102762431 | 0.090055249 |
| P51168 | 895 | SCNN1B | Amiloride-sensitiv | 460 | 1 | 460 | 0.052173913 | 0.023913043 | 0.038043478 |
| A6NMB1 | 896 | SIGLEC16 | Sialic acid-binding | 417 | 1 | 417 | 0.055155875 | 0.033573141 | 0.044364508 |
| Q9UQF0 | 897 | ERVW-1 | Syncytin-1 (Endog | 422 | 1 | 422 | 0.066350711 | 0.066350711 | 0.066350711 |
| Q8WWT9 | 898 | SLC13A3 | Solute carrier fam | 147 | 6 | 34 | 0.06122449 | 0.020408163 | 0.040816327 |
| Q2M3M2 | 899 | SLC5A9 | Sodium/glucose c | 172 | 7 | 44 | 0.046511628 | 0.052325581 | 0.049418605 |
| Q9Y345 | 900 | SLC6A5 | Sodium- and chlo | 101 | 1 | 101 | 0.059405941 | 0.02970297 | 0.044554455 |
| P34741 | 901 | SDC2 | Syndecan-2 (SYN | 125 | 1 | 125 | 0.072 | 0.168 | 0.12 |
| Q9H2E6 | 902 | SEMA6A | Semaphorin-6A (S | 630 | 1 | 630 | 0.041269841 | 0.047619048 | 0.044444444 |
| O94933 | 903 | SLITRK3 | SLIT and NTRK-li | 625 | 1 | 625 | 0.0608 | 0.0416 | 0.0512 |
| Q9BZZ2 | 904 | SIGLEC1 | Sialoadhesin (Sia | 1621 | 1 | 1621 | 0.054287477 | 0.017890191 | 0.036088834 |
| P58743 | 905 | SLC26A5 | Prestin (Solute ca | 108 | 6 | 30 | 0.027777778 | 0.018518519 | 0.023148148 |
| Q9HBX9 | 906 | RXFP1 | Relaxin receptor 1 | 457 | 3 | 408 | 0.026258206 | 0.01750547 | 0.021881838 |
| Q8TCC7 | 907 | SLC22A8 | Solute carrier fam | 151 | 5 | 92 | 0.059602649 | 0.039735099 | 0.049668874 |
| P41440 | 908 | SLC19A1 | Reduced folate tra | 78 | 6 | 25 | 0.064102564 | 0.012820513 | 0.038461538 |

| | | | | | | | | | |
|---|---|---|---|---|---|---|---|---|---|
| Q9HBV2 | 909 | SPACA1 | Sperm acrosome r | 191 | 1 | 191 | 0.031413613 | 0.036649215 | 0.034031414 |
| P31639 | 910 | SLC5A2 | Sodium/glucose c | 240 | 5 | 88 | 0.025 | 0.020833333 | 0.022916667 |
| Q92911 | 911 | SLC5A5 | Sodium/iodide co | 146 | 7 | 59 | 0.04109589 | 0.075342466 | 0.058219178 |
| Q1EHB4 | 912 | SLC5A12 | Sodium-coupled r | 134 | 7 | 45 | 0.067164179 | 0.052238806 | 0.059701493 |
| Q9H3T2 | 913 | SEMA6C | Semaphorin-6C (S | 579 | 1 | 579 | 0.025906736 | 0.029360967 | 0.027633851 |
| O75056 | 914 | SDC3 | Syndecan-3 (SYN | 386 | 1 | 386 | 0.310880829 | 0.204663212 | 0.257772021 |
| Q9UPX0 | 915 | IGSF9B | Protein turtle hom | 701 | 1 | 701 | 0.062767475 | 0.012838802 | 0.037803138 |
| P21731 | 916 | TBXA2R | Thromboxane A2 | 84 | 4 | 28 | 0.05952381 | 0.071428571 | 0.06547619 |
| P59535 | 917 | TAS2R40 | Taste receptor typ | 67 | 4 | 27 | 0.089552239 | 0.014925373 | 0.052238806 |
| Q15109 | 918 | AGER | Advanced glycosy | 319 | 1 | 319 | 0.053291536 | 0.006269592 | 0.029780564 |
| Q9UN76 | 919 | SLC6A14 | Sodium- and chlo | 103 | 1 | 103 | 0.067961165 | 0.087378641 | 0.077669903 |
| P35499 | 920 | SCN4A | Sodium channel p | 341 | 16 | 118 | 0.052785924 | 0.029325513 | 0.041055718 |
| Q6AZY7 | 921 | SCARA3 | Scavenger recepto | 528 | 1 | 528 | 0.041666667 | 0.060606061 | 0.051136364 |
| P30825 | 922 | SLC7A1 | High affinity catio | 120 | 7 | 38 | 0.05 | 0.025 | 0.0375 |
| Q99523 | 923 | SORT1 | Sortilin (100 kDa I | 677 | 1 | 677 | 0.045790251 | 0.019202363 | 0.032496307 |
| Q96LD1 | 924 | SGCZ | Zeta-sarcoglycan | 240 | 1 | 240 | 0 | 0.041666667 | 0.020833333 |
| Q5JXA9 | 925 | SIRPB2 | Signal-regulatory | 254 | 1 | 254 | 0.051181102 | 0.039370079 | 0.045275591 |
| Q9BXA5 | 926 | SUCNR1 | Succinate recepto | 103 | 4 | 29 | 0.038834951 | 0.019417476 | 0.029126214 |
| A6NJZ3 | 927 | OR6C65 | Olfactory receptor | 76 | 4 | 31 | 0.039473684 | 0.026315789 | 0.032894737 |
| Q8NH01 | 928 | OR2T11 | Olfactory receptor | 89 | 4 | 36 | 0.06741573 | 0.02247191 | 0.04494382 |
| O15031 | 929 | PLXNB2 | Plexin-B2 (MM1) | 1177 | 1 | 1177 | 0.034834325 | 0.018691589 | 0.026762957 |
| Q9HCM2 | 930 | PLXNA4 | Plexin-A4 | 1213 | 1 | 1213 | 0.025556472 | 0.018961253 | 0.022258862 |
| Q9UHI7 | 931 | SLC23A1 | Solute carrier fam | 87 | 7 | 34 | 0.034482759 | 0.022988506 | 0.028735632 |
| Q96N87 | 932 | SLC6A18 | Inactive sodium-d | 213 | 6 | 87 | 0.03286385 | 0.018779343 | 0.025821596 |
| P21583 | 933 | KITLG | Kit ligand (Mast c | 188 | 1 | 188 | 0.04787234 | 0.106382979 | 0.07712766 |
| P51170 | 934 | SCNN1G | Amiloride-sensitiv | 464 | 1 | 464 | 0.030172414 | 0.023706897 | 0.026939655 |
| P46721 | 935 | SLCO1A2 | Solute carrier orga | 237 | 6 | 107 | 0.046413502 | 0.025316456 | 0.035864979 |
| Q92629 | 936 | SGCD | Delta-sarcoglycar | 232 | 1 | 232 | 0.051724138 | 0.017241379 | 0.034482759 |
| Q5TFQ8 | 937 | SIRPB1 | Signal-regulatory | 341 | 1 | 341 | 0.082111437 | 0.049853372 | 0.065982405 |
| Q8IYJ0 | 938 | PIANP | PILR alpha-assoc | 146 | 1 | 146 | 0.068493151 | 0.095890411 | 0.082191781 |
| Q9ULL4 | 939 | PLXNB3 | Plexin-B3 | 1210 | 1 | 1210 | 0.023966942 | 0.021487603 | 0.022727273 |
| O43490 | 940 | PROM1 | Prominin-1 (Antig | 626 | 3 | 284 | 0.020766773 | 0.033546326 | 0.02715655 |
| O15244 | 941 | SLC22A2 | Solute carrier fam | 136 | 6 | 106 | 0.036764706 | 0.022058824 | 0.029411765 |
| Q6T423 | 942 | SLC22A25 | Solute carrier fam | 136 | 6 | 114 | 0.044117647 | 0.022058824 | 0.033088235 |
| P48065 | 943 | SLC6A12 | Sodium- and chlo | 72 | 1 | 72 | 0.111111111 | 0.055555556 | 0.083333333 |
| Q6U841 | 944 | SLC4A10 | Sodium-driven ch | 192 | 6 | 72 | 0.041666667 | 0.036458333 | 0.0390625 |
| Q8NBS3 | 945 | SLC4A11 | Sodium bicarbona | 57 | 1 | 57 | 0.105263158 | 0.175438596 | 0.140350877 |
| A0PJK1 | 946 | SLC5A10 | Sodium/glucose c | 128 | 7 | 42 | 0.09375 | 0.0859375 | 0.08984375 |
| Q07837 | 947 | SLC3A1 | Neutral and basic | 576 | 1 | 576 | 0.026041667 | 0.022569444 | 0.024305556 |
| Q8NBJ9 | 948 | SIDT2 | SID1 transmembra | 402 | 6 | 277 | 0.037313433 | 0.024875622 | 0.031094527 |
| Q3SXP7 | 949 | SHISAL1 | Protein shisa-like- | 71 | 1 | 71 | 0.070422535 | 0.070422535 | 0.070422535 |
| Q9C0H2 | 950 | TTYH3 | Protein tweety ho | 272 | 3 | 128 | 0.033088235 | 0.014705882 | 0.023897059 |
| Q9UGT4 | 951 | SUSD2 | Sushi domain-con | 757 | 1 | 757 | 0.06340819 | 0.043593131 | 0.053500661 |
| P08912 | 952 | CHRM5 | Muscarinic acetylc | 78 | 4 | 28 | 0.153846154 | 0.064102564 | 0.108974359 |
| P28223 | 953 | HTR2A | 5-hydroxytryptami | 121 | 4 | 74 | 0 | 0.066115702 | 0.033057851 |
| P28221 | 954 | HTR1D | 5-hydroxytryptami | 75 | 4 | 37 | 0 | 0.12 | 0.06 |
| Q9BY15 | 955 | ADGRE3 | Adhesion G protei | 363 | 4 | 335 | 0.066115702 | 0.074380165 | 0.070247934 |
| P20023 | 956 | CR2 | Complement rece | 950 | 1 | 950 | 0.044210526 | 0.077894737 | 0.061052632 |
| Q96D31 | 957 | ORAI1 | Calcium release-a | 52 | 2 | 39 | 0.134615385 | 0.096153846 | 0.115384615 |
| P35462 | 958 | DRD3 | D(3) dopamine re | 76 | 4 | 31 | 0 | 0.105263158 | 0.052631579 |
| O75388 | 959 | GPR32 | Probable G-protei | 118 | 4 | 43 | 0.042372881 | 0.06779661 | 0.055084746 |
| Q9UPC5 | 960 | GPR34 | Probable G-protei | 120 | 4 | 60 | 0.158333333 | 0.175 | 0.166666667 |
| Q9HC97 | 961 | GPR35 | G-protein coupled | 70 | 4 | 23 | 0 | 0.042857143 | 0.021428571 |
| O15354 | 962 | GPR37 | Prosaposin recept | 310 | 4 | 238 | 0.103225806 | 0.093548387 | 0.098387097 |
| O43194 | 963 | GPR39 | G-protein coupled | 113 | 4 | 44 | 0 | 0.115044248 | 0.057522124 |
| P46089 | 964 | GPR3 | G-protein coupled | 80 | 4 | 41 | 0 | 0.0625 | 0.03125 |
| O15529 | 965 | GPR42 | G-protein coupled | 77 | 4 | 24 | 0 | 0.025974026 | 0.012987013 |
| Q9Y5Y3 | 966 | GPR45 | Probable G-protei | 91 | 4 | 37 | 0.087912088 | 0.098901099 | 0.093406593 |
| P46093 | 967 | GPR4 | G-protein coupled | 71 | 4 | 24 | 0 | 0.028169014 | 0.014084507 |
| Q9Y2T5 | 968 | GPR52 | G-protein coupled | 81 | 4 | 43 | 0 | 0.111111111 | 0.055555556 |
| Q9Y2T6 | 969 | GPR55 | G-protein coupled | 73 | 4 | 21 | 0 | 0.068493151 | 0.034246575 |
| Q9BZJ8 | 970 | GPR61 | G-protein coupled | 96 | 4 | 43 | 0 | 0.114583333 | 0.057291667 |
| P12821 | 971 | ACE | Angiotensin-conve | 1226 | 1 | 1226 | 0.017944535 | 0.015497553 | 0.016721044 |
| Q9BXJ7 | 972 | AMN | Protein amnionles | 337 | 1 | 337 | 0.005934718 | 0.011869436 | 0.008902077 |
| O43306 | 973 | ADCY6 | Adenylate cyclase | 59 | 1 | 59 | 0.169491525 | 0.016949153 | 0.093220339 |
| Q86SQ3 | 974 | ADGRE4P | Putative adhesion | 202 | 4 | 175 | 0.024752475 | 0.044554455 | 0.034653465 |
| Q32M45 | 975 | ANO4 | Anoctamin-4 (Tra | 515 | 5 | 351 | 0.017475728 | 0.011650485 | 0.014563107 |
| Q6UW56 | 976 | ATRAID | All-trans retinoic a | 168 | 1 | 168 | 0 | 0.023809524 | 0.011904762 |
| O95180 | 977 | CACNA1H | Voltage-depende | 433 | 12 | 140 | 0.011547344 | 0.009237875 | 0.01039261 |
| P33151 | 978 | CDH5 | Cadherin-5 (7B4 a | 551 | 1 | 551 | 0.045372051 | 0.012704174 | 0.029038113 |
| O43570 | 979 | CA12 | Carbonic anhydra | 276 | 1 | 276 | 0.032608696 | 0.028985507 | 0.030797101 |
| P08962 | 980 | CD63 | CD63 antigen (Gr | 118 | 2 | 100 | 0.050847458 | 0.059322034 | 0.055084746 |
| P11912 | 981 | CD79A | B-cell antigen rec | 110 | 1 | 110 | 0 | 0.054545455 | 0.027272727 |
| A0A087X1C5 | 982 | CYP2D7 | Putative cytochror | 193 | 2 | 192 | 0 | 0 | 0 |
| P21709 | 983 | EPHA1 | Ephrin type-A rece | 521 | 1 | 521 | 0.044145873 | 0.046065259 | 0.045105566 |
| Q5JZY3 | 984 | EPHA10 | Ephrin type-A rece | 531 | 1 | 531 | 0.054613936 | 0.047080979 | 0.050847458 |
| P54753 | 985 | EPHB3 | Ephrin type-B rece | 525 | 1 | 525 | 0.125714286 | 0.034285714 | 0.08 |
| O60883 | 986 | GPR37L1 | G-protein coupled | 176 | 4 | 108 | 0 | 0.034090909 | 0.017045455 |
| Q9BY21 | 987 | GPR87 | G-protein coupled | 111 | 4 | 46 | 0 | 0.054054054 | 0.027027027 |
| Q8TDU6 | 988 | GPBAR1 | G-protein coupled | 60 | 4 | 18 | 0 | 0.116666667 | 0.058333333 |
| Q13255 | 989 | GRM1 | Metabotropic glut | 609 | 4 | 573 | 0.016420361 | 0.009852217 | 0.013136289 |
| Q8N743 | 990 | KIR3DL3 | Killer cell immuno | 296 | 1 | 296 | 0.047297297 | 0.016891892 | 0.032094595 |
| Q9UL51 | 991 | HCN2 | Potassium/sodiur | 36 | 4 | 22 | 0.027777778 | 0.027777778 | 0.027777778 |
| Q9NY64 | 992 | SLC2A8 | Solute carrier fam | 88 | 6 | 26 | 0.022727273 | 0.068181818 | 0.045454545 |
| P01889 | 993 | HLA-B | HLA class I histoc | 284 | 1 | 284 | 0.021126761 | 0.01056338 | 0.01584507 |
| Q8N6C5 | 994 | IGSF1 | Immunoglobulin s | 1244 | 2 | 755 | 0.038585209 | 0.024919614 | 0.031752412 |
| Q9UHX3 | 995 | ADGRE2 | Adhesion G protei | 550 | 4 | 516 | 0.045454545 | 0.045454545 | 0.045454545 |
| P78325 | 996 | ADAM8 | Disintegrin and m | 638 | 1 | 638 | 0.026645768 | 0.028213166 | 0.027429467 |
| Q8WVV5 | 997 | BTN2A2 | Butyrophilin subfa | 232 | 1 | 232 | 0.034482759 | 0.012931034 | 0.023706897 |
| P78410 | 998 | BTN3A2 | Butyrophilin subfa | 218 | 1 | 218 | 0.018348624 | 0.004587156 | 0.01146789 |
| Q9UBN1 | 999 | CACNG4 | Voltage-depende | 104 | 2 | 76 | 0.048076923 | 0.019230769 | 0.033653846 |
| P19440 | 1000 | GGT1 | Glutathione hydro | 542 | 1 | 542 | 0.044280443 | 0.014760148 | 0.029520295 |
| P60893 | 1001 | GPR85 | Probable G-protei | 75 | 4 | 29 | 0.08 | 0.053333333 | 0.066666667 |
| Q9NZH0 | 1002 | GPRC5B | G-protein coupled | 67 | 4 | 27 | 0 | 0.029850746 | 0.014925373 |
| Q02413 | 1003 | DSG1 | Desmoglein-1 (Ca | 498 | 1 | 498 | 0.108433735 | 0.010040161 | 0.059236948 |
| P21728 | 1004 | DRD1 | D(1A) dopamine r | 70 | 4 | 28 | 0 | 0.1 | 0.05 |
| B6SEH8 | 1005 | ERVV-1 | Endogenous retro | 299 | 1 | 299 | 0.08361204 | 0.076923077 | 0.080267559 |
| P54762 | 1006 | EPHB1 | Ephrin type-B rece | 522 | 1 | 522 | 0.04789272 | 0.040229885 | 0.044061303 |
| P29323 | 1007 | EPHB2 | Ephrin type-B rece | 524 | 1 | 524 | 0.053435115 | 0.030534351 | 0.041984733 |
| O15552 | 1008 | FFAR2 | Free fatty acid rec | 71 | 4 | 25 | 0.028169014 | 0.028169014 | 0.028169014 |
| P23945 | 1009 | FSHR | Follicle-stimulatin | 398 | 4 | 348 | 0.012562814 | 0.037688442 | 0.025125628 |
| Q8N1C3 | 1010 | GABRG1 | Gamma-aminobut | 236 | 1 | 236 | 0.021186441 | 0.012711864 | 0.016949153 |
| Q9BXC1 | 1011 | GPR174 | Probable G-protei | 84 | 4 | 26 | 0.047619048 | 0.023809524 | 0.035714286 |
| Q08334 | 1012 | IL10RB | Interleukin-10 rec | 200 | 1 | 200 | 0.035 | 0.02 | 0.0275 |
| Q8NAC3 | 1013 | IL17RC | Interleukin-17 rec | 517 | 1 | 517 | 0.02901354 | 0.03868472 | 0.03384913 |
| P26951 | 1014 | IL3RA | Interleukin-3 rece | 286 | 1 | 286 | 0.031468531 | 0.020979021 | 0.026223776 |
| Q9UKX5 | 1015 | ITGA11 | Integrin alpha-11 | 1118 | 1 | 1118 | 0.030411449 | 0.02236136 | 0.026386404 |
| P11215 | 1016 | ITGAM | Integrin alpha-M ( | 1087 | 1 | 1087 | 0.036798528 | 0.031278749 | 0.034038638 |
| Q6UXV1 | 1017 | IZUMO2 | Izumo sperm-egg | 165 | 1 | 165 | 0.012121212 | 0.006060606 | 0.009090909 |
| P18084 | 1018 | ITGB5 | Integrin beta-5 | 695 | 1 | 695 | 0.014388489 | 0.041726619 | 0.028057554 |
| P48551 | 1019 | IFNAR2 | Interferon alpha/b | 216 | 1 | 216 | 0.037037037 | 0.027777778 | 0.032407407 |
| P29275 | 1020 | ADORA2B | Adenosine recepto | 57 | 4 | 33 | 0.070175439 | 0.035087719 | 0.052631579 |
| Q75V66 | 1021 | ANO5 | Anoctamin-5 (Gna | 266 | 4 | 100 | 0.07518797 | 0.033834586 | 0.054511278 |
| P10966 | 1022 | CD8B | T-cell surface glyc | 148 | 1 | 148 | 0 | 0.047297297 | 0.023648649 |

| | | | | | | | | | |
|---|---|---|---|---|---|---|---|---|---|
| P54764 | 1023 | EPHA4 | Ephrin type-A rece | 527 | 1 | 527 | 0.028462998 | 0.024667932 | 0.026565465 |
| P54756 | 1024 | EPHA5 | Ephrin type-A rece | 548 | 1 | 548 | 0.02919708 | 0.038321168 | 0.033759124 |
| Q8N441 | 1025 | FGFRL1 | Fibroblast growth | 353 | 1 | 353 | 0.056657224 | 0.036827195 | 0.04674221 |
| Q96PJ5 | 1026 | FCRL4 | Fc receptor-like pr | 367 | 1 | 367 | 0.032697548 | 0.010899183 | 0.021798365 |
| P10912 | 1027 | GHR | Growth hormone r | 245 | 1 | 245 | 0.040816327 | 0.044897959 | 0.042857143 |
| P47869 | 1028 | GABRA2 | Gamma-aminobut | 222 | 1 | 222 | 0.054054054 | 0.013513514 | 0.033783784 |
| Q6UXV0 | 1029 | GFRAL | GDNF family rece | 332 | 1 | 332 | 0.024096386 | 0.039156627 | 0.031626506 |
| P13224 | 1030 | GP1BB | Platelet glycoprot | 120 | 1 | 120 | 0.05 | 0.025 | 0.0375 |
| P11230 | 1031 | CHRNB1 | Acetylcholine rece | 220 | 1 | 220 | 0.009090909 | 0.004545455 | 0.006818182 |
| Q15758 | 1032 | SLC1A5 | Neutral amino aci | 126 | 5 | 70 | 0.055555556 | 0.079365079 | 0.067460317 |
| Q16570 | 1033 | ACKR1 | Atypical chemokin | 114 | 4 | 62 | 0.052631579 | 0.01754386 | 0.035087719 |
| O00590 | 1034 | ACKR2 | Atypical chemokin | 107 | 4 | 49 | 0.074766355 | 0.065420561 | 0.070093458 |
| P41595 | 1035 | HTR2B | 5-hydroxytryptami | 107 | 4 | 55 | 0 | 0.08411215 | 0.042056075 |
| Q8N3J6 | 1036 | CADM2 | Cell adhesion mol | 342 | 1 | 342 | 0.078947368 | 0.040935673 | 0.05994152 |
| P20273 | 1037 | CD22 | B-cell receptor CD | 667 | 1 | 667 | 0 | 0.016491754 | 0.008245877 |
| Q8IWA5 | 1038 | SLC44A2 | Choline transporte | 339 | 5 | 177 | 0.020648968 | 0.029498525 | 0.025073746 |
| P01903 | 1039 | HLA-DRA | HLA class II histo | 190 | 1 | 190 | 0.047368421 | 0.036842105 | 0.042105263 |
| P61565 | 1040 | ERVK-21 | Endogenous retro | 542 | 1 | 542 | 0.036900369 | 0.016605166 | 0.026752768 |
| P47211 | 1041 | GALR1 | Galanin receptor t | 80 | 4 | 35 | 0.05 | 0.05 | 0.05 |
| Q9BWV1 | 1042 | BOC | Brother of CDO (F | 824 | 1 | 824 | 0.053398058 | 0.033980583 | 0.04368932 |
| Q9UJ99 | 1043 | CDH22 | Cadherin-22 (Pitu | 588 | 1 | 588 | 0.197278912 | 0.005102041 | 0.101190476 |
| P55283 | 1044 | CDH4 | Cadherin-4 (Retin | 564 | 1 | 564 | 0.042553191 | 0.010638298 | 0.026595745 |
| Q7Z692 | 1045 | CEACAM19 | Carcinoembryonic | 124 | 1 | 124 | 0.048387097 | 0.008064516 | 0.028225806 |
| O94985 | 1046 | CLSTN1 | Calsyntenin-1 (Al | 830 | 1 | 830 | 0.025301205 | 0.006024096 | 0.015662651 |
| Q14118 | 1047 | DAG1 | Dystroglycan (Dys | 95 | 1 | 95 | 0.073684211 | 0.031578947 | 0.052631579 |
| Q1HG44 | 1048 | DUOXA2 | Dual oxidase matu | 144 | 3 | 105 | 0.0625 | 0.020833333 | 0.041666667 |
| Q9NQ84 | 1049 | GPRC5C | G-protein coupled | 86 | 4 | 31 | 0 | 0.011627907 | 0.005813953 |
| Q14833 | 1050 | GRM4 | Metabotropic glut | 605 | 4 | 554 | 0.036363636 | 0.016528926 | 0.026446281 |
| Q9H222 | 1051 | ABCG5 | ATP-binding cass | 99 | 3 | 73 | 0 | 0.03030303 | 0.015151515 |
| O60242 | 1052 | ADGRB3 | Adhesion G protei | 887 | 4 | 855 | 0.03945885 | 0.06313416 | 0.051296505 |
| Q8IZP9 | 1053 | ADGRG2 | Adhesion G-prote | 624 | 4 | 589 | 0.094551282 | 0.099358974 | 0.096955128 |
| P35368 | 1054 | ADRA1B | Alpha-1B adrener | 75 | 4 | 44 | 0.133333333 | 0.133333333 | 0.133333333 |
| P14415 | 1055 | ATP1B2 | Sodium/potassiur | 222 | 1 | 222 | 0.040540541 | 0.036036036 | 0.038288288 |
| P78348 | 1056 | ASIC1 | Acid-sensing ion c | 357 | 1 | 357 | 0.014005602 | 0.008403361 | 0.011204482 |
| Q13705 | 1057 | ACVR2B | Activin receptor ty | 118 | 1 | 118 | 0.050847458 | 0.016949153 | 0.033898305 |
| P36894 | 1058 | BMPR1A | Bone morphogene | 128 | 1 | 128 | 0.015625 | 0.046875 | 0.03125 |
| Q13698 | 1059 | CACNA1S | Voltage-depende | 315 | 16 | 60 | 0.028571429 | 0.012698413 | 0.020634921 |
| Q9NR16 | 1060 | CD163L1 | Scavenger recept | 1318 | 1 | 1318 | 0.005311077 | 0.018968134 | 0.012139605 |
| Q6ZS10 | 1061 | CLEC17A | C-type lectin dom | 184 | 1 | 184 | 0 | 0.016304348 | 0.008152174 |
| Q15760 | 1062 | GPR19 | Probable G-protei | 114 | 4 | 68 | 0.052631579 | 0.078947368 | 0.065789474 |
| Q14CZ8 | 1063 | HEPACAM | Hepatocyte cell a | 206 | 1 | 206 | 0.077669903 | 0.038834951 | 0.058252427 |
| Q92838 | 1064 | EDA | Ectodysplasin-A ( | 328 | 1 | 328 | 0.036585366 | 0.085365854 | 0.06097561 |
| Q8TDQ0 | 1065 | HAVCR2 | Hepatitis A virus c | 180 | 1 | 180 | 0.055555556 | 0.072222222 | 0.063888889 |
| Q13261 | 1066 | IL15RA | Interleukin-15 rec | 174 | 1 | 174 | 0.206896552 | 0.206896552 | 0.206896552 |
| Q13651 | 1067 | IL10RA | Interleukin-10 rec | 213 | 1 | 213 | 0.061032864 | 0.028169014 | 0.044600939 |
| Q5SQ64 | 1068 | LY6G6F | Lymphocyte antig | 218 | 1 | 218 | 0.013761468 | 0.009174312 | 0.01146789 |
| Q9HBG7 | 1069 | LY9 | T-lymphocyte surl | 406 | 1 | 406 | 0 | 0.036945813 | 0.018472906 |
| P14151 | 1070 | SELL | L-selectin (CD62 | 293 | 1 | 293 | 0 | 0.027303754 | 0.013651877 |
| P16581 | 1071 | SELE | E-selectin (CD62 | 534 | 1 | 534 | 0 | 0.050561798 | 0.025280899 |
| Q13477 | 1072 | MADCAM1 | Mucosal addressi | 298 | 1 | 298 | 0.117449664 | 0.093959732 | 0.105704698 |
| P16410 | 1073 | CTLA4 | Cytotoxic T-lymph | 125 | 1 | 125 | 0.048 | 0.016 | 0.032 |
| Q9Y4D2 | 1074 | DAGLA | Diacylglycerol lipa | 29 | 2 | 16 | 0.034482759 | 0.034482759 | 0.034482759 |
| P58658 | 1075 | EVA1C | Protein eva-1 hon | 273 | 1 | 273 | 0.018315018 | 0.021978022 | 0.02014652 |
| Q9NZU1 | 1076 | FLRT1 | Leucine-rich repe | 503 | 1 | 503 | 0.047713718 | 0.039761431 | 0.043737575 |
| O00144 | 1077 | FZD9 | Frizzled-9 (Fz-9) | 295 | 4 | 206 | 0.033898305 | 0.020338983 | 0.027118644 |
| Q9NPG1 | 1078 | FZD3 | Frizzled-3 (Fz-3) | 270 | 4 | 182 | 0.025925926 | 0.011111111 | 0.018518519 |
| Q14330 | 1079 | GPR18 | N-arachidonyl gly | 87 | 4 | 31 | 0 | 0.045977011 | 0.022988506 |
| Q9UJ42 | 1080 | GPR160 | Probable G-protei | 56 | 4 | 22 | 0.035714286 | 0.053571429 | 0.044642857 |
| Q9H3N8 | 1081 | HRH4 | Histamine H4 rece | 65 | 4 | 19 | 0.092307692 | 0.092307692 | 0.092307692 |
| Q9HB29 | 1082 | IL1RL2 | Interleukin-1 rece | 315 | 1 | 315 | 0.050793651 | 0.025396825 | 0.038095238 |
| Q9NP60 | 1083 | IL1RAPL2 | X-linked interleuk | 337 | 1 | 337 | 0 | 0.017804154 | 0.008902077 |
| Q6UXC1 | 1084 | MAMDC4 | Apical endosomal | 1128 | 1 | 1128 | 0.034574468 | 0.029255319 | 0.031914894 |
| Q9NQ90 | 1085 | ANO2 | Anoctamin-2 (Tra | 256 | 4 | 103 | 0.03515625 | 0.03515625 | 0.03515625 |
| O94778 | 1086 | AQP8 | Aquaporin-8 (AQF | 76 | 3 | 27 | 0.026315789 | 0.026315789 | 0.026315789 |
| P34972 | 1087 | CNR2 | Cannabinoid rece | 69 | 4 | 32 | 0.028985507 | 0.014492754 | 0.02173913 |
| Q10589 | 1088 | BST2 | Bone marrow stro | 112 | 1 | 112 | 0.026785714 | 0.044642857 | 0.035714286 |
| Q96KV6 | 1089 | BTN2A3P | Putative butyroph | 218 | 1 | 218 | 0.036697248 | 0.032110092 | 0.03440367 |
| Q13873 | 1090 | BMPR2 | Bone morphogene | 123 | 1 | 123 | 0.089430894 | 0.024390244 | 0.056910569 |
| O43497 | 1091 | CACNA1G | Voltage-depende | 416 | 12 | 135 | 0.052884615 | 0.021634615 | 0.037259615 |
| A6NJW9 | 1092 | CD8B2 | T-cell surface glyc | 151 | 1 | 151 | 0 | 0.039735099 | 0.01986755 |
| P30203 | 1093 | CD6 | T-cell differentiati | 384 | 1 | 384 | 0 | 0.0390625 | 0.01953125 |
| Q9NYQ6 | 1094 | CELSR1 | Cadherin EGF LAC | 2474 | 4 | 2447 | 0.078415521 | 0.030315279 | 0.0543654 |
| P32248 | 1095 | CCR7 | C-C chemokine re | 97 | 4 | 34 | 0 | 0.051546392 | 0.025773196 |
| Q9HBJ8 | 1096 | CLTRN | Collectrin (Transn | 126 | 1 | 126 | 0.03968254 | 0.015873016 | 0.027777778 |
| Q86SJ2 | 1097 | AMIGO2 | Amphoterin-induc | 358 | 1 | 358 | 0.036312849 | 0.033519553 | 0.034916201 |
| Q86SQ4 | 1098 | ADGRG6 | Adhesion G-prote | 861 | 4 | 824 | 0.059233449 | 0.041811847 | 0.050522648 |
| Q9H2U9 | 1099 | ADAM7 | Disintegrin and m | 649 | 1 | 649 | 0.018489985 | 0.024653313 | 0.021571649 |
| O75077 | 1100 | ADAM23 | Disintegrin and m | 505 | 1 | 505 | 0.023762376 | 0.021782178 | 0.022772277 |
| Q16515 | 1101 | ASIC2 | Acid-sensing ion c | 368 | 1 | 368 | 0.048913043 | 0.008152174 | 0.028532609 |
| Q86T13 | 1102 | CLEC14A | C-type lectin dom | 375 | 1 | 375 | 0 | 0.069333333 | 0.034666667 |
| Q8WXI8 | 1103 | CLEC4D | C-type lectin dom | 176 | 1 | 176 | 0 | 0.017045455 | 0.008522727 |
| P50895 | 1104 | BCAM | Basal cell adhesio | 515 | 1 | 515 | 0.048543689 | 0.013592233 | 0.031067961 |
| Q8IVY1 | 1105 | C1orf210 | Type III endosom | 28 | 1 | 28 | 0.285714286 | 0.214285714 | 0.25 |
| A0ZSE6 | 1106 | TMEM30CP | Cell cycle control p | 57 | 1 | 57 | 0.070175439 | 0.035087719 | 0.052631579 |
| Q16602 | 1107 | CALCRL | Calcitonin gene-re | 168 | 4 | 123 | 0.023809524 | 0.029761905 | 0.026785714 |
| Q12864 | 1108 | CDH17 | Cadherin-17 (Inte | 764 | 1 | 764 | 0.062827225 | 0.011780105 | 0.037303665 |
| P01730 | 1109 | CD4 | T-cell surface glyc | 370 | 1 | 370 | 0 | 0.013513514 | 0.006756757 |
| Q6ZTQ4 | 1110 | CDHR3 | Cadherin-related | 693 | 1 | 693 | 0.041847042 | 0.017316017 | 0.02958153 |
| Q6NUJ2 | 1111 | C11orf87 | Uncharacterized p | 37 | 1 | 37 | 0.108108108 | 0.108108108 | 0.108108108 |
| Q9UGM1 | 1112 | CHRNA9 | Neuronal acetylch | 211 | 1 | 211 | 0.028436019 | 0.009478673 | 0.018957346 |
| Q14246 | 1113 | ADGRE1 | Adhesion G protei | 616 | 4 | 578 | 0.058441558 | 0.081168831 | 0.069805195 |
| Q96PE1 | 1114 | ADGRA2 | Adhesion G protei | 762 | 4 | 737 | 0.034120735 | 0.039370079 | 0.036745407 |
| P35414 | 1115 | APLNR | Apelin receptor (A | 78 | 4 | 33 | 0.051282051 | 0.025641026 | 0.038461538 |
| P54709 | 1116 | ATP1B3 | Sodium/potassiur | 222 | 1 | 222 | 0.027027027 | 0.009009009 | 0.018018018 |
| Q86TY3 | 1117 | ARMH4 | Armadillo-like heli | 686 | 1 | 686 | 0.06851312 | 0.209912536 | 0.139212828 |
| Q5VV63 | 1118 | ATRNL1 | Attractin-like prot | 1177 | 1 | 1177 | 0.032285472 | 0.053525913 | 0.042905692 |
| Q6UX41 | 1119 | BTNL8 | Butyrophilin-like p | 220 | 1 | 220 | 0.009090909 | 0.004545455 | 0.006818182 |
| P04920 | 1120 | SLC4A2 | Anion exchange p | 52 | 1 | 52 | 0.096153846 | 0.115384615 | 0.105769231 |
| O00555 | 1121 | CACNA1A | Voltage-depende | 374 | 12 | 86 | 0.024064171 | 0.010695187 | 0.017379679 |
| Q8NFZ8 | 1122 | CADM4 | Cell adhesion mol | 303 | 1 | 303 | 0.0330033 | 0.01320132 | 0.02310231 |
| P20138 | 1123 | CD33 | Myeloid cell surfa | 241 | 1 | 241 | 0.099585062 | 0.033195021 | 0.066390041 |
| Q9BUF7 | 1124 | CRB3 | Protein crumbs ho | 32 | 1 | 32 | 1.09375 | 0.40625 | 0.75 |
| P08173 | 1125 | CHRM4 | Muscarinic acetyl | 80 | 4 | 30 | 0.05 | 0.125 | 0.0875 |
| Q9H172 | 1126 | ABCG4 | ATP-binding cass | 82 | 3 | 63 | 0 | 0 | 0 |
| Q9H221 | 1127 | ABCG8 | ATP-binding cass | 79 | 3 | 62 | 0 | 0.025316456 | 0.012658228 |
| P30939 | 1128 | HTR1F | 5-hydroxytryptami | 74 | 4 | 28 | 0 | 0.027027027 | 0.013513514 |
| P28335 | 1129 | HTR2C | 5-hydroxytryptami | 68 | 4 | 19 | 0 | 0.073529412 | 0.036764706 |
| P28566 | 1130 | HTR1E | 5-hydroxytryptami | 62 | 4 | 21 | 0 | 0.112903226 | 0.056451613 |
| Q01718 | 1131 | MC2R | Adrenocorticotrop | 68 | 4 | 24 | 0.073529412 | 0.029411765 | 0.051470588 |
| P07307 | 1132 | ASGR2 | Asialoglycoprotei | 231 | 1 | 231 | 0.060606061 | 0.047619048 | 0.054112554 |
| Q9ULY5 | 1133 | CLEC4E | C-type lectin dom | 178 | 1 | 178 | 0 | 0.016853933 | 0.008426966 |
| Q8N1N0 | 1134 | CLEC4F | C-type lectin dom | 528 | 1 | 528 | 0 | 0.075757576 | 0.037878788 |
| Q6UXB4 | 1135 | CLEC4G | C-type lectin dom | 240 | 1 | 240 | 0 | 0.0375 | 0.01875 |
| Q9BZ76 | 1136 | CNTNAP3 | Contactin-associa | 1219 | 1 | 1219 | 0.037735849 | 0.034454471 | 0.03609516 |

| | | | | | | | | | |
|---|---|---|---|---|---|---|---|---|---|
| P52569 | 1137 | SLC7A2 | Cationic amino ac | 120 | 7 | 37 | 0.058333333 | 0.05 | 0.054166667 |
| O60503 | 1138 | ADCY9 | Adenylate cyclase | 89 | 6 | 33 | 0.101123596 | 0.04494382 | 0.073033708 |
| P13945 | 1139 | ADRB3 | Beta-3 adrenergic | 89 | 4 | 35 | 0.101123596 | 0.078651685 | 0.08988764 |
| Q9H6X2 | 1140 | ANTXR1 | Anthrax toxin rece | 288 | 1 | 288 | 0.173611111 | 0.010416667 | 0.092013889 |
| Q9H2X3 | 1141 | CLEC4M | C-type lectin dom | 328 | 1 | 328 | 0 | 0.012195122 | 0.006097561 |
| Q9NY25 | 1142 | CLEC5A | C-type lectin dom | 160 | 1 | 160 | 0 | 0.025 | 0.0125 |
| Q6EIG7 | 1143 | CLEC6A | C-type lectin dom | 167 | 1 | 167 | 0 | 0.005988024 | 0.002994012 |
| Q9BXN2 | 1144 | CLEC7A | C-type lectin dom | 181 | 1 | 181 | 0 | 0 | 0 |
| Q6UXN8 | 1145 | CLEC9A | C-type lectin dom | 184 | 1 | 184 | 0 | 0.016304348 | 0.008152174 |
| P78357 | 1146 | CNTNAP1 | Contactin-associa | 1263 | 1 | 1263 | 0.026128266 | 0.026128266 | 0.026128266 |
| Q9UQC9 | 1147 | CLCA2 | Calcium-activated | 869 | 1 | 869 | 0.048331415 | 0.025316456 | 0.036823936 |
| Q9UHC6 | 1148 | CNTNAP2 | Contactin-associa | 1234 | 1 | 1234 | 0.027552674 | 0.025931929 | 0.026742301 |
| O95727 | 1149 | CRTAM | Cytotoxic and regu | 269 | 1 | 269 | 0.092936803 | 0.05204461 | 0.072490706 |
| P27037 | 1150 | ACVR2A | Activin receptor ty | 115 | 1 | 115 | 0.043478261 | 0.034782609 | 0.039130435 |
| Q16790 | 1151 | CA9 | Carbonic anhydra | 376 | 1 | 376 | 0.013297872 | 0.029255319 | 0.021276596 |
| P12830 | 1152 | CDH1 | Cadherin-1 (CAM | 554 | 1 | 554 | 0.081227437 | 0.003610108 | 0.042418773 |
| P29965 | 1153 | CD40LG | CD40 ligand (CD4 | 214 | 1 | 214 | 0.023364486 | 0.004672897 | 0.014018692 |
| Q496F6 | 1154 | CD300E | CMRF35-like mole | 155 | 1 | 155 | 0.064516129 | 0.058064516 | 0.061290323 |
| P02708 | 1155 | CHRNA1 | Acetylcholine rece | 234 | 1 | 234 | 0.025641026 | 0 | 0.012820513 |
| Q8NFM4 | 1156 | ADCY4 | Adenylate cyclase | 26 | 1 | 26 | 0.076923077 | 0.076923077 | 0.076923077 |
| P58335 | 1157 | ANTXR2 | Anthrax toxin rece | 284 | 1 | 284 | 0.073943662 | 0.007042254 | 0.040492958 |
| Q6UVW9 | 1158 | CLEC2A | C-type lectin dom | 125 | 1 | 125 | 0 | 0.032 | 0.016 |
| Q9UMR7 | 1159 | CLEC4A | C-type lectin dom | 167 | 1 | 167 | 0 | 0.02994012 | 0.01497006 |
| Q9UIR0 | 1160 | BTNL2 | Butyrophilin-like p | 431 | 1 | 431 | 0.018561485 | 0.006960557 | 0.012761021 |
| P48509 | 1161 | CD151 | CD151 antigen (G | 125 | 2 | 108 | 0.016 | 0.04 | 0.028 |
| P30542 | 1162 | ADORA1 | Adenosine recept | 55 | 4 | 29 | 0.072727273 | 0.036363636 | 0.054545455 |
| Q6QNK2 | 1163 | ADGRD1 | Adhesion G-prote | 570 | 4 | 544 | 0.061403509 | 0.043859649 | 0.052631579 |
| Q8IZF6 | 1164 | ADGRG4 | Adhesion G-prote | 2741 | 4 | 2713 | 0.194819409 | 0.27800073 | 0.236410069 |
| O95490 | 1165 | ADGRL2 | Adhesion G protei | 849 | 4 | 825 | 0.047114252 | 0.044758539 | 0.045936396 |
| P51693 | 1166 | APLP1 | Amyloid-like prote | 541 | 1 | 541 | 0.029574861 | 0.057301294 | 0.043438078 |
| P41181 | 1167 | AQP2 | Aquaporin-2 (AQF | 36 | 4 | 19 | 0.055555556 | 0.027777778 | 0.041666667 |
| P15144 | 1168 | ANPEP | Aminopeptidase N | 934 | 1 | 934 | 0.043897216 | 0.028907923 | 0.03640257 |
| Q8IZS8 | 1169 | CACNA2D3 | Voltage-depende | 1039 | 1 | 1039 | 0.024061598 | 0.013474495 | 0.018768046 |
| Q01668 | 1170 | CACNA1D | Voltage-depende | 413 | 12 | 89 | 0.024213075 | 0.007263923 | 0.015738499 |
| Q5VU97 | 1171 | CACHD1 | VWFA and cache | 1059 | 1 | 1059 | 0.037771483 | 0.017941454 | 0.027856468 |
| P55286 | 1172 | CDH8 | Cadherin-8 | 559 | 1 | 559 | 0.177101968 | 0.008944544 | 0.093023256 |
| P33681 | 1173 | CD80 | T-lymphocyte acti | 207 | 1 | 207 | 0 | 0.033816425 | 0.016908213 |
| P06127 | 1174 | CD5 | T-cell surface glyc | 347 | 1 | 347 | 0 | 0.037463977 | 0.018731988 |
| P15391 | 1175 | CD19 | B-lymphocyte ant | 271 | 1 | 271 | 0 | 0.040590406 | 0.020295203 |
| Q9UHN6 | 1176 | CEMIP2 | Cell surface hyalu | 1279 | 1 | 1279 | 0.077404222 | 0.017200938 | 0.04730258 |
| O00421 | 1177 | CCRL2 | C-C chemokine re | 108 | 4 | 42 | 0 | 0.018518519 | 0.009259259 |
| Q2HXU8 | 1178 | CLEC12B | C-type lectin dom | 211 | 1 | 211 | 0 | 0.018957346 | 0.009478673 |
| Q96NU0 | 1179 | CNTNAP3B | Contactin-associa | 1219 | 1 | 1219 | 0.03609516 | 0.032813782 | 0.034454471 |
| P05538 | 1180 | HLA-DQB2 | HLA class II histo | 196 | 1 | 196 | 0.051020408 | 0.020408163 | 0.035714286 |
| Q9UJ71 | 1181 | CD207 | C-type lectin dom | 263 | 1 | 263 | 0 | 0.02661597 | 0.013307985 |
| Q9C0A0 | 1182 | CNTNAP4 | Contactin-associa | 1215 | 1 | 1215 | 0.037037037 | 0.03127572 | 0.034156379 |
| Q8WYK1 | 1183 | CNTNAP5 | Contactin-associa | 1212 | 1 | 1212 | 0.042079208 | 0.031353135 | 0.036716172 |
| Q8N608 | 1184 | DPP10 | Inactive dipeptidy | 740 | 1 | 740 | 0.027027027 | 0.02027027 | 0.023648649 |
| P27487 | 1185 | DPP4 | Dipeptidyl peptida | 737 | 1 | 737 | 0.043419267 | 0.02578019 | 0.034599729 |
| Q9NRD9 | 1186 | DUOX1 | Dual oxidase 1 (E | 604 | 3 | 574 | 0.026490066 | 0.033112583 | 0.029801325 |
| Q9NRD8 | 1187 | DUOX2 | Dual oxidase 2 (E | 621 | 3 | 575 | 0.01610306 | 0.022544283 | 0.019323671 |
| Q96PZ7 | 1188 | CSMD1 | CUB and sushi do | 3460 | 1 | 3460 | 0.044508671 | 0.065028902 | 0.054768786 |
| Q9HC73 | 1189 | CRLF2 | Cytokine receptor | 208 | 1 | 208 | 0.048076923 | 0.028846154 | 0.038461538 |
| Q8IZY2 | 1190 | ABCA7 | Phospholipid-tran | 779 | 2 | 506 | 0.037227214 | 0.026957638 | 0.032092426 |
| Q9GZZ6 | 1191 | CHRNA10 | Neuronal acetylch | 212 | 1 | 212 | 0.028301887 | 0.009433962 | 0.018867925 |
| Q8IZF5 | 1192 | ADGRF3 | Adhesion G-prote | 799 | 4 | 749 | 0.038798498 | 0.028785982 | 0.03379224 |
| Q13444 | 1193 | ADAM15 | Disintegrin and m | 489 | 1 | 489 | 0.036809816 | 0.049079755 | 0.042944785 |
| P29972 | 1194 | AQP1 | Aquaporin-1 (AQF | 39 | 4 | 20 | 0.076923077 | 0.051282051 | 0.064102564 |
| Q9HD20 | 1195 | ATP13A1 | Endoplasmic retic | 305 | 4 | 178 | 0.016393443 | 0.013114754 | 0.014754098 |
| Q9NYX4 | 1196 | CALY | Neuron-specific v | 86 | 1 | 86 | 0.011627907 | 0.011627907 | 0.011627907 |
| Q9P0X4 | 1197 | CACNA1I | Voltage-depende | 441 | 12 | 144 | 0.015873016 | 0.009070295 | 0.012471655 |
| O75309 | 1198 | CDH16 | Cadherin-16 (Kidr | 767 | 1 | 767 | 0.032594524 | 0.005215124 | 0.018904824 |
| P29973 | 1199 | CNGA1 | cGMP-gated catic | 60 | 3 | 42 | 0.1 | 0.016666667 | 0.058333333 |
| Q9NR61 | 1200 | DLL4 | Delta-like protein | 502 | 1 | 502 | 0.09561753 | 0.071713147 | 0.083665339 |
| Q09428 | 1201 | ABCC8 | ATP-binding cass | 255 | 8 | 114 | 0 | 0.007843137 | 0.003921569 |
| P20309 | 1202 | CHRM3 | Muscarinic acetyl | 110 | 4 | 66 | 0.154545455 | 0.2 | 0.177272727 |
| P0DMS8 | 1203 | ADORA3 | Adenosine recept | 57 | 4 | 28 | 0.122807018 | 0.087719298 | 0.105263158 |
| Q15822 | 1204 | CHRNA2 | Neuronal acetylch | 237 | 1 | 237 | 0.063291139 | 0.033755274 | 0.048523207 |
| P11229 | 1205 | CHRM1 | Muscarinic acetyl | 73 | 4 | 23 | 0.082191781 | 0.082191781 | 0.082191781 |
| Q8IUN9 | 1206 | CLEC10A | C-type lectin dom | 255 | 1 | 255 | 0 | 0.047058824 | 0.023529412 |
| Q04656 | 1207 | ATP7A | Copper-transporti | 95 | 4 | 38 | 0.063157895 | 0.021052632 | 0.042105263 |
| Q3KPI0 | 1208 | CEACAM21 | Carcinoembryonic | 205 | 1 | 205 | 0.082926829 | 0.009756098 | 0.046341463 |
| O75871 | 1209 | CEACAM4 | Carcinoembryonic | 119 | 1 | 119 | 0.050420168 | 0.033613445 | 0.042016807 |
| Q9BZW8 | 1210 | CD244 | Natural killer cell r | 207 | 1 | 207 | 0.053140097 | 0.038647343 | 0.04589372 |
| P25100 | 1211 | ADRA1D | Alpha-1D adrener | 121 | 4 | 94 | 0.066115702 | 0.115702479 | 0.090909091 |
| O95622 | 1212 | ADCY5 | Adenylate cyclase | 52 | 1 | 52 | 0.038461538 | 0.038461538 | 0.038461538 |
| Q07075 | 1213 | ENPEP | Glutamyl aminope | 917 | 1 | 917 | 0.165757906 | 0.029443839 | 0.097600872 |
| Q9UHP7 | 1214 | CLEC2D | C-type lectin dom | 131 | 1 | 131 | 0 | 0.015267176 | 0.007633588 |
| Q8WTT0 | 1215 | CLEC4C | C-type lectin dom | 168 | 1 | 168 | 0 | 0.017857143 | 0.008928571 |
| P42081 | 1216 | CD86 | T-lymphocyte acti | 223 | 1 | 223 | 0 | 0.035874439 | 0.01793722 |
| P21926 | 1217 | CD9 | CD9 antigen (5H9 | 104 | 2 | 83 | 0.019230769 | 0.019230769 | 0.019230769 |
| P29017 | 1218 | CD1C | T-cell surface glyc | 284 | 1 | 284 | 0 | 0.017605634 | 0.008802817 |
| Q16280 | 1219 | CNGA2 | Cyclic nucleotide- | 120 | 3 | 81 | 0.016666667 | 0.008333333 | 0.0125 |
| Q9NZV1 | 1220 | CRIM1 | Cysteine-rich mot | 904 | 1 | 904 | 0.024336283 | 0.080752212 | 0.052544248 |
| P07333 | 1221 | CSF1R | Macrophage color | 497 | 1 | 497 | 0.104627767 | 0.024144869 | 0.064386318 |
| P49682 | 1222 | CXCR3 | C-X-C chemokine | 108 | 4 | 52 | 0 | 0.055555556 | 0.027777778 |
| Q5SY80 | 1223 | CATSPERE | Cation channel sp | 883 | 1 | 883 | 0.031710079 | 0.012457531 | 0.022083805 |
| Q9UNQ0 | 1224 | ABCG2 | Broad substrate s | 91 | 3 | 73 | 0.032967033 | 0.021978022 | 0.027472527 |
| Q15825 | 1225 | CHRNA6 | Neuronal acetylch | 213 | 1 | 213 | 0.028169014 | 0.009389671 | 0.018779343 |
| P48960 | 1226 | ADGRE5 | Adhesion G protei | 570 | 4 | 531 | 0.040350877 | 0.071929825 | 0.056140351 |
| Q9UM73 | 1227 | ALK | ALK tyrosine kinas | 1019 | 1 | 1019 | 0.049067713 | 0.045142296 | 0.047105005 |
| O43506 | 1228 | ADAM20 | Disintegrin and m | 486 | 1 | 486 | 0.020576132 | 0.03909465 | 0.029835391 |
| Q6UXG3 | 1229 | CD300LG | CMRF35-like mole | 228 | 1 | 228 | 0.122807018 | 0.140350877 | 0.131578947 |
| P17927 | 1230 | CR1 | Complement rece | 1929 | 1 | 1929 | 0.038880249 | 0.062208398 | 0.050544323 |
| Q6PCB8 | 1231 | EMB | Embigin | 227 | 1 | 227 | 0.074889868 | 0.052863436 | 0.063876652 |
| Q96LA5 | 1232 | FCRL2 | Fc receptor-like pr | 381 | 1 | 381 | 0.041994751 | 0.01312336 | 0.027559055 |
| Q6V0I7 | 1233 | FAT4 | Protocadherin Fat | 4465 | 1 | 4465 | 0.043225084 | 0.008734602 | 0.025979843 |
| Q9Y3Q7 | 1234 | ADAM18 | Disintegrin and m | 510 | 1 | 510 | 0.007843137 | 0.043137255 | 0.025490196 |
| P08588 | 1235 | ADRB1 | Beta-1 adrenergic | 94 | 4 | 54 | 0.063829787 | 0.074468085 | 0.069148936 |
| P05026 | 1236 | ATP1B1 | Sodium/potassiur | 240 | 1 | 240 | 0.025 | 0.016666667 | 0.020833333 |
| P46663 | 1237 | BDKRB1 | B1 bradykinin rece | 89 | 4 | 39 | 0.056179775 | 0.08988764 | 0.073033708 |
| Q00975 | 1238 | CACNA1B | Voltage-depende | 376 | 12 | 86 | 0.021276596 | 0.002659574 | 0.011968085 |
| Q9P296 | 1239 | C5AR2 | C5a anaphylatoxir | 97 | 4 | 37 | 0.092783505 | 0.020618557 | 0.056701031 |
| P28907 | 1240 | CD38 | ADP-ribosyl cycla | 257 | 1 | 257 | 0 | 0.023346304 | 0.011673152 |
| P51686 | 1241 | CCR9 | C-C chemokine re | 85 | 4 | 47 | 0 | 0.105882353 | 0.052941176 |
| P21918 | 1242 | DRD5 | D(1B) dopamine r | 108 | 4 | 42 | 0 | 0.046296296 | 0.023148148 |
| O95866 | 1243 | MPIG6B | Megakaryocyte ar | 124 | 1 | 124 | 0.040322581 | 0.048387097 | 0.044354839 |
| Q92847 | 1244 | GHSR | Growth hormone s | 102 | 4 | 39 | 0.235294118 | 0.058823529 | 0.147058824 |
| I3L273 | 1245 | GFY | Golgi-associated | 389 | 1 | 389 | 0 | 0.228791774 | 0.114395887 |
| O60706 | 1246 | ABCC9 | ATP-binding cass | 222 | 8 | 96 | 0 | 0.018018018 | 0.009009009 |
| Q9HAR2 | 1247 | ADGRL3 | Adhesion G protei | 885 | 4 | 846 | 0.059887006 | 0.044067797 | 0.051977401 |
| P20594 | 1248 | NPR2 | Atrial natriuretic p | 435 | 1 | 435 | 0.03908046 | 0.029885057 | 0.034482759 |
| Q6PL45 | 1249 | BRICD5 | BRICHOS domain | 182 | 1 | 182 | 0.038461538 | 0.027472527 | 0.032967033 |
| P16671 | 1250 | CD36 | Platelet glycoprot | 409 | 1 | 409 | 0.039119804 | 0.024449878 | 0.031784841 |

| | | | | | | | | | |
|---|---|---|---|---|---|---|---|---|---|
| P19022 | 1251 | CDH2 | Cadherin-2 (CDw: | 564 | 1 | 564 | 0.063829787 | 0.014184397 | 0.039007092 |
| Q4KMG0 | 1252 | CDON | Cell adhesion mol | 937 | 1 | 937 | 0.131270011 | 0.032017076 | 0.081643543 |
| P32246 | 1253 | CCR1 | C-C chemokine re | 89 | 4 | 33 | 0 | 0.078651685 | 0.039325843 |
| Q5KU26 | 1254 | COLEC12 | Collectin-12 (Coll | 683 | 1 | 683 | 0.043923865 | 0.068814056 | 0.05636896 |
| Q16832 | 1255 | DDR2 | Discoidin domain- | 377 | 1 | 377 | 0.039787798 | 0.034482759 | 0.037135279 |
| Q9NPB9 | 1256 | ACKR4 | Atypical chemokin | 97 | 4 | 41 | 0.06185567 | 0.041237113 | 0.051546392 |
| Q13639 | 1257 | HTR4 | 5-hydroxytryptami | 76 | 4 | 33 | 0 | 0.026315789 | 0.013157895 |
| P17787 | 1258 | CHRNB2 | Neuronal acetylch | 221 | 2 | 207 | 0.009049774 | 0.009049774 | 0.009049774 |
| P50052 | 1259 | AGTR2 | Type-2 angiotens | 106 | 4 | 44 | 0.103773585 | 0.122641509 | 0.113207547 |
| Q86WK6 | 1260 | AMIGO1 | Amphoterin-induc | 344 | 1 | 344 | 0.01744186 | 0.020348837 | 0.018895349 |
| O94910 | 1261 | ADGRL1 | Adhesion G protei | 857 | 4 | 833 | 0.043173862 | 0.037339557 | 0.040256709 |
| O43184 | 1262 | ADAM12 | Disintegrin and m | 500 | 1 | 500 | 0.014 | 0.038 | 0.026 |
| Q9BY67 | 1263 | CADM1 | Cell adhesion mol | 329 | 1 | 329 | 0.389057751 | 0.066869301 | 0.227963526 |
| P09564 | 1264 | CD7 | T-cell antigen CD | 154 | 1 | 154 | 0 | 0.058441558 | 0.029220779 |
| P40259 | 1265 | CD79B | B-cell antigen rec | 130 | 1 | 130 | 0 | 0.030769231 | 0.015384615 |
| P29016 | 1266 | CD1B | T-cell surface glyc | 285 | 1 | 285 | 0 | 0.01754386 | 0.00877193 |
| A6H8M9 | 1267 | CDHR4 | Cadherin-related | 669 | 1 | 669 | 0.068759342 | 0.020926756 | 0.044843049 |
| Q8TDQ1 | 1268 | CD300LF | CMRF35-like mole | 136 | 1 | 136 | 0.051470588 | 0.066176471 | 0.058823529 |
| Q9UGN4 | 1269 | CD300A | CMRF35-like mole | 162 | 1 | 162 | 0.12962963 | 0.12345679 | 0.12654321 |
| Q8IZS7 | 1270 | CLECL1 | C-type lectin-like | 78 | 1 | 78 | 0 | 0.012820513 | 0.006410256 |
| Q8WY07 | 1271 | SLC7A3 | Cationic amino ac | 102 | 7 | 33 | 0.019607843 | 0.019607843 | 0.019607843 |
| P56937 | 1272 | HSD17B7 | 3-keto-steroid red | 228 | 1 | 228 | 0 | 0.030701754 | 0.015350877 |
| O95477 | 1273 | ABCA1 | Phospholipid-tran | 880 | 2 | 596 | 0.032954545 | 0.026136364 | 0.029545455 |
| Q8N2K0 | 1274 | ABHD12 | Lysophosphatidyl | 302 | 1 | 302 | 0.013245033 | 0.006622517 | 0.009933775 |
| P08195 | 1275 | SLC3A2 | 4F2 cell-surface a | 424 | 1 | 424 | 0.030660377 | 0.016509434 | 0.023584906 |
| Q86WK7 | 1276 | AMIGO3 | Amphoterin-induc | 363 | 1 | 363 | 0.016528926 | 0.03030303 | 0.023415978 |
| Q9P0K1 | 1277 | ADAM22 | Disintegrin and m | 513 | 1 | 513 | 0.015594542 | 0.037037037 | 0.026315789 |
| P35348 | 1278 | ADRA1A | Alpha-1A adrener | 56 | 4 | 26 | 0.214285714 | 0.125 | 0.169642857 |
| Q7Z6A9 | 1279 | BTLA | B- and T-lymphoc | 126 | 1 | 126 | 0 | 0.03968254 | 0.01984127 |
| Q8IU99 | 1280 | CALHM1 | Calcium homeosta | 62 | 2 | 51 | 0.016129032 | 0.016129032 | 0.016129032 |
| Q9Y271 | 1281 | CYSLTR1 | Cysteinyl leukotrie | 108 | 4 | 30 | 0.064814815 | 0.074074074 | 0.069444444 |
| O14843 | 1282 | FFAR3 | Free fatty acid rec | 75 | 4 | 24 | 0.026666667 | 0.026666667 | 0.026666667 |
| P12319 | 1283 | FCER1A | High affinity immu | 179 | 1 | 179 | 0.05027933 | 0.044692737 | 0.047486034 |
| Q14517 | 1284 | FAT1 | Protocadherin Fat | 4159 | 1 | 4159 | 0.03582592 | 0.006251503 | 0.021038711 |
| Q02643 | 1285 | GHRHR | Growth hormone- | 160 | 4 | 109 | 0.0375 | 0.0125 | 0.025 |
| P48169 | 1286 | GABRA4 | Gamma-aminobut | 222 | 1 | 222 | 0.040540541 | 0.018018018 | 0.029279279 |
| O14764 | 1287 | GABRD | Gamma-aminobut | 231 | 1 | 231 | 0.017316017 | 0.008658009 | 0.012987013 |
| Q9NS66 | 1288 | GPR173 | Probable G-protei | 81 | 4 | 27 | 0.061728395 | 0.061728395 | 0.061728395 |
| Q96P66 | 1289 | GPR101 | Probable G-protei | 87 | 4 | 34 | 0.137931034 | 0.137931034 | 0.137931034 |
| P29274 | 1290 | ADORA2A | Adenosine recept | 52 | 4 | 29 | 0.038461538 | 0 | 0.019230769 |
| P36896 | 1291 | ACVR1B | Activin receptor ty | 102 | 1 | 102 | 0.009803922 | 0.029411765 | 0.019607843 |
| P08913 | 1292 | ADRA2A | Alpha-2A adrener | 90 | 4 | 47 | 0.044444444 | 0.066666667 | 0.055555556 |
| Q9BZ11 | 1293 | ADAM33 | Disintegrin and m | 671 | 1 | 671 | 0.020864382 | 0.035767511 | 0.028315946 |
| Q9UKJ8 | 1294 | ADAM21 | Disintegrin and m | 484 | 1 | 484 | 0.024793388 | 0.035123967 | 0.029958678 |
| P07306 | 1295 | ASGR1 | Asialoglycoprotein | 229 | 1 | 229 | 0.017467249 | 0.043668122 | 0.030567686 |
| P56817 | 1296 | BACE1 | Beta-secretase 1 | 435 | 1 | 435 | 0.027586207 | 0.020689655 | 0.024137931 |
| Q06432 | 1297 | CACNG1 | Voltage-depende | 101 | 2 | 78 | 0.03960396 | 0.01980198 | 0.02970297 |
| Q9H159 | 1298 | CDH19 | Cadherin-19 | 552 | 1 | 552 | 0.048913043 | 0.012681159 | 0.030797101 |
| P16070 | 1299 | CD44 | CD44 antigen (CD | 628 | 1 | 628 | 0.152866242 | 0.24522293 | 0.199044586 |
| Q9ULB4 | 1300 | CDH9 | Cadherin-9 | 561 | 1 | 561 | 0.040998217 | 0.007130125 | 0.024064171 |
| Q9NQW8 | 1301 | CNGB3 | Cyclic nucleotide- | 112 | 3 | 65 | 0.008928571 | 0.008928571 | 0.008928571 |
| Q96F05 | 1302 | C11orf24 | Uncharacterized p | 379 | 1 | 379 | 0.308707124 | 0.340369393 | 0.324538259 |
| Q7Z408 | 1303 | CSMD2 | CUB and sushi do | 3407 | 1 | 3407 | 0.044027003 | 0.060757264 | 0.052392134 |
| Q8NFT8 | 1304 | DNER | Delta and Notch-l | 605 | 1 | 605 | 0.059504132 | 0.110743802 | 0.085123967 |
| P04440 | 1305 | HLA-DPB1 | HLA class II histo | 195 | 1 | 195 | 0.01025641 | 0.015384615 | 0.012820513 |
| Q6P995 | 1306 | FAM171B | Protein FAM171B | 320 | 1 | 320 | 0.040625 | 0.0125 | 0.0265625 |
| P22732 | 1307 | SLC2A5 | Solute carrier fam | 69 | 6 | 28 | 0.014492754 | 0.014492754 | 0.014492754 |
| Q9UGQ3 | 1308 | SLC2A6 | Solute carrier fam | 75 | 6 | 34 | 0.026666667 | 0.066666667 | 0.046666667 |
| P02730 | 1309 | SLC4A1 | Band 3 anion tran | 82 | 4 | 39 | 0.012195122 | 0.048780488 | 0.030487805 |
| Q9NPF0 | 1310 | CD320 | CD320 antigen (8 | 193 | 1 | 193 | 0.139896373 | 0.186528497 | 0.163212435 |
| Q53GD3 | 1311 | SLC44A4 | Choline transporte | 339 | 5 | 173 | 0.04719764 | 0.056047198 | 0.051622419 |
| P04839 | 1312 | CYBB | Cytochrome b-245 | 102 | 3 | 45 | 0.039215686 | 0.029411765 | 0.034313725 |
| Q9UKF2 | 1313 | ADAM30 | Disintegrin and m | 488 | 1 | 488 | 0.016393443 | 0.032786885 | 0.024590164 |
| Q6UY09 | 1314 | CEACAM20 | Carcinoembryonic | 419 | 1 | 419 | 0.045346062 | 0.026252983 | 0.035799523 |
| Q96JP9 | 1315 | CDHR1 | Cadherin-related | 680 | 1 | 680 | 0.047058824 | 0.005882353 | 0.026470588 |
| P01911 | 1316 | HLA-DRB1 | HLA class II histo | 197 | 1 | 197 | 0.040609137 | 0.010152284 | 0.025380711 |
| Q9Y6X5 | 1317 | ENPP4 | Bis(5'-adenosyl)- | 391 | 1 | 391 | 0 | 0.025575448 | 0.012787724 |
| P37023 | 1318 | ACVRL1 | Serine/threonine- | 96 | 1 | 96 | 0.0625 | 0.083333333 | 0.072916667 |
| Q8N350 | 1319 | CBARP | Voltage-depende | 44 | 1 | 44 | 0.386363636 | 0.022727273 | 0.204545455 |
| P55287 | 1320 | CDH11 | Cadherin-11 (OSF | 563 | 1 | 563 | 0.035523979 | 0.003552398 | 0.019538188 |
| Q13634 | 1321 | CDH18 | Cadherin-18 (Cad | 554 | 1 | 554 | 0.041516245 | 0.005415162 | 0.023465704 |
| Q9H251 | 1322 | CDH23 | Cadherin-23 (Oto | 3040 | 1 | 3040 | 0.043092105 | 0.011513158 | 0.027302632 |
| P43005 | 1323 | SLC1A1 | Excitatory amino a | 155 | 5 | 90 | 0.070967742 | 0.090322581 | 0.080645161 |
| P06340 | 1324 | HLA-DOA | HLA class II histo | 191 | 1 | 191 | 0.020942408 | 0.015706806 | 0.018324607 |
| O14842 | 1325 | FFAR1 | Free fatty acid rec | 63 | 4 | 35 | 0.047619048 | 0 | 0.023809524 |
| Q96RD9 | 1326 | FCRL5 | Fc receptor-like pr | 835 | 1 | 835 | 0.050299401 | 0.016766467 | 0.033532934 |
| O95342 | 1327 | ABCB11 | Bile salt export pu | 105 | 5 | 63 | 0.047619048 | 0.038095238 | 0.042857143 |
| Q9BYF1 | 1328 | ACE2 | Angiotensin-conv | 722 | 1 | 722 | 0.05401662 | 0.009695291 | 0.031855956 |
| O95264 | 1329 | HTR3B | 5-hydroxytryptami | 237 | 3 | 216 | 0 | 0.025316456 | 0.012658228 |
| Q96K78 | 1330 | ADGRG7 | Adhesion G-prote | 492 | 4 | 411 | 0.052845528 | 0.06504065 | 0.058943089 |
| P41597 | 1331 | CCR2 | C-C chemokine re | 97 | 4 | 41 | 0 | 0.092783505 | 0.046391753 |
| Q08708 | 1332 | CD300C | CMRF35-like mole | 162 | 1 | 162 | 0.111111111 | 0.111111111 | 0.111111111 |
| Q9NS75 | 1333 | CYSLTR2 | Cysteinyl leukotrie | 118 | 4 | 41 | 0.059322034 | 0.06779661 | 0.063559322 |
| Q9H4D0 | 1334 | CLSTN2 | Calsyntenin-2 (Al | 810 | 1 | 810 | 0.027160494 | 0.012345679 | 0.019753086 |
| O75355 | 1335 | ENTPD3 | Ectonucleoside tri | 441 | 1 | 441 | 0.038548753 | 0.015873016 | 0.027210884 |
| Q9N2K0 | 1336 | 0 | HERV-H_2q24.3 p | 487 | 1 | 487 | 0 | 0.024640657 | 0.012320329 |
| P08908 | 1337 | HTR1A | 5-hydroxytryptami | 68 | 4 | 35 | 0 | 0.132352941 | 0.066176471 |
| Q96PS8 | 1338 | AQP10 | Aquaporin-10 (AQ | 54 | 3 | 33 | 0.12962963 | 0.055555556 | 0.092592593 |
| O75882 | 1339 | ATRN | Attractin (DPPT-L | 1195 | 1 | 1195 | 0.034309623 | 0.060251046 | 0.047280335 |
| P21554 | 1340 | CNR1 | Cannabinoid rece | 154 | 4 | 115 | 0.038961039 | 0.051948052 | 0.045454545 |
| P35070 | 1341 | BTC | Probetacellulin [C | 86 | 1 | 86 | 0.046511628 | 0.093023256 | 0.069767442 |
| Q96Q91 | 1342 | SLC4A9 | Anion exchange p | 72 | 1 | 72 | 0.027777778 | 0.027777778 | 0.027777778 |
| P43681 | 1343 | CHRNA4 | Neuronal acetylch | 213 | 1 | 213 | 0.023474178 | 0.014084507 | 0.018779343 |
| Q04771 | 1344 | ACVR1 | Activin receptor ty | 102 | 1 | 102 | 0.078431373 | 0.058823529 | 0.068627451 |
| Q8NC01 | 1345 | CLEC1A | C-type lectin dom | 206 | 1 | 206 | 0 | 0.024271845 | 0.012135922 |
| Q92478 | 1346 | CLEC2B | C-type lectin dom | 123 | 1 | 123 | 0 | 0.024390244 | 0.012195122 |
| P51164 | 1347 | ATP4B | Potassium-transp | 233 | 1 | 233 | 0.042918455 | 0.051502146 | 0.0472103 |
| P54289 | 1348 | CACNA2D1 | Voltage-depende | 1048 | 1 | 1048 | 0.026717557 | 0.02480916 | 0.025763359 |
| P82279 | 1349 | CRB1 | Protein crumbs ho | 1321 | 1 | 1321 | 0.04012112 | 0.090840273 | 0.065480696 |
| Q08554 | 1350 | DSC1 | Desmocollin-1 (C | 556 | 1 | 556 | 0.043165468 | 0.005395683 | 0.024280576 |
| P32926 | 1351 | DSG3 | Desmoglein-3 (13 | 565 | 1 | 565 | 0.037168142 | 0.019469027 | 0.028318584 |
| P13765 | 1352 | HLA-DOB | HLA class II histo | 197 | 1 | 197 | 0.020304569 | 0.015228426 | 0.017766497 |
| Q04844 | 1353 | CHRNE | Acetylcholine rece | 244 | 3 | 218 | 0.028688525 | 0.008196721 | 0.018442623 |
| P36544 | 1354 | CHRNA7 | Neuronal acetylch | 207 | 1 | 207 | 0.028985507 | 0.014492754 | 0.02173913 |
| Q9HBW9 | 1355 | ADGRL4 | Adhesion G protei | 454 | 4 | 412 | 0.066079295 | 0.066079295 | 0.066079295 |
| P16066 | 1356 | NPR1 | Atrial natriuretic p | 440 | 1 | 440 | 0.036363636 | 0.015909091 | 0.026136364 |
| Q9P126 | 1357 | CLEC1B | C-type lectin dom | 174 | 1 | 174 | 0 | 0.017241379 | 0.00862069 |
| O00478 | 1358 | BTN3A3 | Butyrophilin subfa | 218 | 1 | 218 | 0.018348624 | 0.004587156 | 0.01146789 |
| Q14574 | 1359 | DSC3 | Desmocollin-3 (C | 554 | 1 | 554 | 0.079422383 | 0.009025271 | 0.044223827 |
| P34969 | 1360 | HTR7 | 5-hydroxytryptami | 133 | 4 | 82 | 0 | 0.067669173 | 0.033834586 |
| P25106 | 1361 | ACKR3 | Atypical chemokin | 105 | 4 | 39 | 0.066666667 | 0.038095238 | 0.052380952 |
| P30556 | 1362 | AGTR1 | Type-1 angiotens | 81 | 4 | 29 | 0.148148148 | 0.061728395 | 0.104938272 |
| P13569 | 1363 | CFTR | Cystic fibrosis tran | 99 | 6 | 38 | 0.03030303 | 0.01010101 | 0.02020202 |
| Q5VTT2 | 1364 | C9orf135 | Protein C9orf135 | 122 | 1 | 122 | 0.016393443 | 0.049180328 | 0.032786885 |

| | | | | | | | | |
|---|---|---|---|---|---|---|---|---|
| Q96HD1 | 1365 | CRELD1 | Protein disulfide is | 346 | 2 | 332 | 0.023121387 | 0.043352601 | 0.033236994 |
| Q8TD84 | 1366 | DSCAML1 | Down syndrome c | 1572 | 1 | 1572 | 0.052798982 | 0.017811705 | 0.035305344 |
| O14514 | 1367 | ADGRB1 | Adhesion G protei | 950 | 4 | 917 | 0.045263158 | 0.063157895 | 0.054210526 |
| Q99965 | 1368 | ADAM2 | Disintegrin and m | 511 | 1 | 511 | 0.009784736 | 0.035225049 | 0.022504892 |
| Q9UKF5 | 1369 | ADAM29 | Disintegrin and m | 480 | 1 | 480 | 0.033333333 | 0.035416667 | 0.034375 |
| O00481 | 1370 | BTN3A1 | Butyrophilin subfa | 224 | 1 | 224 | 0.022321429 | 0.004464286 | 0.013392857 |
| Q7KYR7 | 1371 | BTN2A1 | Butyrophilin subfa | 219 | 1 | 219 | 0.0456621 | 0.01826484 | 0.03196347 |
| Q9Y5Z0 | 1372 | BACE2 | Beta-secretase 2 | 452 | 1 | 452 | 0.050884956 | 0.030973451 | 0.040929204 |
| P32238 | 1373 | CCKAR | Cholecystokinin re | 95 | 4 | 40 | 0.052631579 | 0.052631579 | 0.052631579 |
| P13688 | 1374 | CEACAM1 | Carcinoembryonic | 393 | 1 | 393 | 0.10178117 | 0.048346056 | 0.075063613 |
| Q9NYQ7 | 1375 | CELSR3 | Cadherin EGF LA( | 2536 | 4 | 2507 | 0.071766562 | 0.038249211 | 0.055007886 |
| P51681 | 1376 | CCR5 | C-C chemokine re | 88 | 4 | 31 | 0 | 0.034090909 | 0.017045455 |
| Q1HG43 | 1377 | DUOXA1 | Dual oxidase matu | 154 | 3 | 110 | 0.155844156 | 0.032467532 | 0.094155844 |
| P21917 | 1378 | DRD4 | D(4) dopamine re | 77 | 4 | 28 | 0 | 0.051948052 | 0.025974026 |
| P47898 | 1379 | HTR5A | 5-hydroxytryptami | 86 | 4 | 39 | 0 | 0.104651163 | 0.052325581 |
| P55291 | 1380 | CDH15 | Cadherin-15 (Cad | 545 | 1 | 545 | 0.031192661 | 0.00733945 | 0.019266055 |
| Q07108 | 1381 | CD69 | Early activation ar | 137 | 1 | 137 | 0.01459854 | 0.00729927 | 0.010948905 |
| P40198 | 1382 | CEACAM3 | Carcinoembryonic | 120 | 1 | 120 | 0.058333333 | 0.016666667 | 0.0375 |
| Q99467 | 1383 | CD180 | CD180 antigen (L | 602 | 1 | 602 | 0.033222591 | 0.026578073 | 0.029900332 |
| Q15762 | 1384 | CD226 | CD226 antigen (D | 235 | 1 | 235 | 0.153191489 | 0.042553191 | 0.09787234 |
| Q6UXZ3 | 1385 | CD300LD | CMRF35-like mole | 146 | 1 | 146 | 0.150684932 | 0.123287671 | 0.136986301 |
| P61073 | 1386 | CXCR4 | C-X-C chemokine | 87 | 4 | 37 | 0 | 0.057471264 | 0.028735632 |
| P25025 | 1387 | CXCR2 | C-X-C chemokine | 105 | 4 | 47 | 0 | 0.038095238 | 0.019047619 |
| Q6UY11 | 1388 | DLK2 | Protein delta hom | 279 | 1 | 279 | 0.032258065 | 0.053763441 | 0.043010753 |
| Q9Y5L3 | 1389 | ENTPD2 | Ectonucleoside tri | 433 | 1 | 433 | 0.03926097 | 0.025404157 | 0.032332564 |
| P34910 | 1390 | EVI2B | Protein EVI2B (Ec | 180 | 1 | 180 | 0.422222222 | 0.227777778 | 0.325 |
| P46098 | 1391 | HTR3A | 5-hydroxytryptami | 228 | 3 | 217 | 0 | 0.030701754 | 0.015350877 |
| O60840 | 1392 | CACNA1F | Voltage-depende | 405 | 12 | 89 | 0.019753086 | 0.004938272 | 0.012345679 |
| Q86UP0 | 1393 | CDH24 | Cadherin-24 | 596 | 1 | 596 | 0.035234899 | 0.005033557 | 0.020134228 |
| P55289 | 1394 | CDH12 | Cadherin-12 (Brai | 554 | 1 | 554 | 0.025270758 | 0.005415162 | 0.01534296 |
| P34810 | 1395 | CD68 | Macrosialin (Gp11 | 297 | 1 | 297 | 0.235690236 | 0.239057239 | 0.237373737 |
| P15509 | 1396 | CSF2RA | Granulocyte-macr | 297 | 1 | 297 | 0.043771044 | 0.037037037 | 0.04040404 |
| O15431 | 1397 | SLC31A1 | High affinity copp | 62 | 2 | 60 | 0.14516129 | 0.064516129 | 0.10483871 |
| P43146 | 1398 | DCC | Netrin receptor D( | 1071 | 1 | 1071 | 0.049486461 | 0.025210084 | 0.037348273 |
| P25101 | 1399 | EDNRA | Endothelin-1 rece | 129 | 4 | 59 | 0.046511628 | 0.093023256 | 0.069767442 |
| P30926 | 1400 | CHRNB4 | Neuronal acetylch | 241 | 3 | 214 | 0.066390041 | 0.020746888 | 0.043568465 |
| Q70Z44 | 1401 | HTR3D | 5-hydroxytryptami | 228 | 3 | 207 | 0 | 0.00877193 | 0.004385965 |
| P18825 | 1402 | ADRA2C | Alpha-2C adrener | 86 | 4 | 50 | 0.081395349 | 0.081395349 | 0.081395349 |
| Q86SQ6 | 1403 | ADGRA1 | Adhesion G protei | 51 | 4 | 19 | 0.019607843 | 0.019607843 | 0.019607843 |
| Q86Y34 | 1404 | ADGRG3 | Adhesion G protei | 310 | 4 | 251 | 0.041935484 | 0.022580645 | 0.032258065 |
| Q16853 | 1405 | AOC3 | Membrane primar | 736 | 1 | 736 | 0.032608696 | 0.024456522 | 0.028532609 |
| Q9UHC3 | 1406 | ASIC3 | Acid-sensing ion ( | 379 | 1 | 379 | 0.034300792 | 0.023746702 | 0.029023747 |
| P32247 | 1407 | BRS3 | Bombesin recepto | 111 | 4 | 40 | 0.081081081 | 0.117117117 | 0.099099099 |
| Q9UMD9 | 1408 | COL17A1 | Collagen alpha-1( | 1008 | 1 | 1008 | 0.049603175 | 0.113095238 | 0.081349206 |
| Q13324 | 1409 | CRHR2 | Corticotropin-rele | 141 | 4 | 107 | 0.04964539 | 0.035460993 | 0.042553191 |
| P08172 | 1410 | CHRM2 | Muscarinic acetyl | 65 | 4 | 21 | 0.123076923 | 0.153846154 | 0.138461538 |
| Q16671 | 1411 | AMHR2 | Anti-Muellerian h | 131 | 1 | 131 | 0.083969466 | 0.099236641 | 0.091603053 |
| Q13443 | 1412 | ADAM9 | Disintegrin and m | 668 | 1 | 668 | 0.017964072 | 0.025449102 | 0.021706587 |
| O60241 | 1413 | ADGRB2 | Adhesion G protei | 940 | 4 | 903 | 0.056382979 | 0.074468085 | 0.065425532 |
| Q5XXA6 | 1414 | ANO1 | Anoctamin-1 (Dis | 192 | 4 | 86 | 0.046875 | 0.020833333 | 0.033854167 |
| Q8J025 | 1415 | APCDD1 | Protein APCDD1 ( | 465 | 1 | 465 | 0.019354839 | 0.03655914 | 0.027956989 |
| Q9HDC9 | 1416 | APMAP | Adipocyte plasma | 354 | 1 | 354 | 0.019774011 | 0.002824859 | 0.011299435 |
| Q13936 | 1417 | CACNA1C | Voltage-depende | 335 | 16 | 59 | 0.041791045 | 0.011940299 | 0.026865672 |
| P32970 | 1418 | CD70 | CD70 antigen (CD | 154 | 1 | 154 | 0.071428571 | 0.038961039 | 0.055194805 |
| Q6UVK1 | 1419 | CSPG4 | Chondroitin sulfat | 2194 | 1 | 2194 | 0.031905196 | 0.027347311 | 0.029626253 |
| Q02487 | 1420 | DSC2 | Desmocollin-2 (Ca | 558 | 1 | 558 | 0.055555556 | 0.008960573 | 0.032258065 |
| P42892 | 1421 | ECE1 | Endothelin-conve | 680 | 1 | 680 | 0.030882353 | 0.020588235 | 0.025735294 |
| Q8IZF2 | 1422 | ADGRF5 | Adhesion G protei | 1024 | 4 | 984 | 0.059570313 | 0.033203125 | 0.046386719 |
| Q13410 | 1423 | BTN1A1 | Butyrophilin subfa | 215 | 1 | 215 | 0.013953488 | 0.013953488 | 0.013953488 |
| Q86VB7 | 1424 | CD163 | Scavenger recept | 1008 | 1 | 1008 | 0.025793651 | 0.023809524 | 0.024801587 |
| Q9ULB5 | 1425 | CDH7 | Cadherin-7 | 579 | 1 | 579 | 0.039723661 | 0.006908463 | 0.023316062 |
| Q9HCU4 | 1426 | CELSR2 | Cadherin EGF LA( | 2475 | 3 | 2348 | 0.033939394 | 0.021010101 | 0.027474747 |
| Q9H6B4 | 1427 | CLMP | CXADR-like memb | 216 | 1 | 216 | 0.013888889 | 0.009259259 | 0.011574074 |
| P78310 | 1428 | CXADR | Coxsackievirus an | 217 | 1 | 217 | 0.059907834 | 0.004608295 | 0.032258065 |
| Q86XM0 | 1429 | CATSPERD | Cation channel sp | 697 | 1 | 697 | 0.037302726 | 0.012912482 | 0.025107604 |
| Q5T1A1 | 1430 | DCST2 | DC-STAMP doma | 132 | 3 | 67 | 0 | 0.022727273 | 0.011363636 |
| Q6UWV6 | 1431 | ENPP7 | Ectonucleotide py | 411 | 1 | 411 | 0.04136253 | 0.03163017 | 0.03649635 |
| P60507 | 1432 | ERVFC1 | Endogenous retro | 495 | 1 | 495 | 0.066666667 | 0.058585859 | 0.062626263 |
| Q9ULW2 | 1433 | FZD10 | Frizzled-10 (Fz-1( | 288 | 4 | 204 | 0.027777778 | 0.03125 | 0.029513889 |
| Q6ZNI0 | 1434 | GCNT7 | Beta-1,3-galactos | 404 | 1 | 404 | 0.012376238 | 0.022277228 | 0.017326733 |
| Q9NYM4 | 1435 | GPR83 | Probable G-protei | 108 | 4 | 53 | 0.092592593 | 0.083333333 | 0.087962963 |
| Q16538 | 1436 | GPR162 | Probable G-protei | 61 | 4 | 20 | 0.016393443 | 0.016393443 | 0.016393443 |
| P41594 | 1437 | GRM5 | Metabotropic glut | 589 | 4 | 558 | 0.020373514 | 0.013582343 | 0.016977929 |
| O75054 | 1438 | IGSF3 | Immunoglobulin s | 1104 | 1 | 1104 | 0.025362319 | 0.025362319 | 0.025362319 |
| Q8N6P7 | 1439 | IL22RA1 | Interleukin-22 rec | 212 | 1 | 212 | 0.08490566 | 0.014150943 | 0.049528302 |
| P06213 | 1440 | INSR | Insulin receptor (I | 923 | 2 | 730 | 0.086673889 | 0.054171181 | 0.070422535 |
| Q8NHK3 | 1441 | KIR2DL5B | Killer cell immuno | 216 | 1 | 216 | 0.074074074 | 0.064814815 | 0.069444444 |
| P48048 | 1442 | KCNJ1 | ATP-sensitive inw | 32 | 2 | 24 | 0 | 0 | 0 |
| Q96L42 | 1443 | KCNH8 | Potassium voltage | 62 | 4 | 40 | 0.064516129 | 0.032258065 | 0.048387097 |
| Q6UWJ8 | 1444 | CD164L2 | CD164 sialomucin | 111 | 1 | 111 | 0.063063063 | 0.09009009 | 0.076576577 |
| Q15878 | 1445 | CACNA1E | Voltage-depende | 397 | 12 | 86 | 0.020151134 | 0.002518892 | 0.011335013 |
| Q9ULX7 | 1446 | CA14 | Carbonic anhydra | 274 | 1 | 274 | 0.018248175 | 0.01459854 | 0.016423358 |
| Q902F8 | 1447 | ERVK-8 | Endogenous retro | 542 | 1 | 542 | 0.036900369 | 0.016605166 | 0.026752768 |
| P22413 | 1448 | ENPP1 | Ectonucleotide py | 827 | 1 | 827 | 0.026602177 | 0.020556227 | 0.023579202 |
| A1KXE4 | 1449 | FAM168B | Myelin-associated | 99 | 1 | 99 | 0.171717172 | 0.191919192 | 0.181818182 |
| Q3ZCQ3 | 1450 | FAM174B | Membrane protein | 63 | 1 | 63 | 0.142857143 | 0.253968254 | 0.198412698 |
| P04626 | 1451 | ERBB2 | Receptor tyrosine | 629 | 1 | 629 | 0.031796502 | 0.025437202 | 0.028616852 |
| Q9UNN8 | 1452 | PROCR | Endothelial protei | 192 | 1 | 192 | 0.046875 | 0.041666667 | 0.044270833 |
| Q9NZU0 | 1453 | FLRT3 | Leucine-rich repe | 499 | 1 | 499 | 0.06012024 | 0.052104208 | 0.056112224 |
| Q8WWV6 | 1454 | FCAMR | High affinity immu | 433 | 1 | 433 | 0.115473441 | 0.159353349 | 0.137413395 |
| P12318 | 1455 | FCGR2A | Low affinity immu | 183 | 1 | 183 | 0.049180328 | 0.005464481 | 0.027322404 |
| Q96P67 | 1456 | GPR82 | Probable G-protei | 107 | 4 | 63 | 0.046728972 | 0.018691589 | 0.03271028 |
| Q8TDV0 | 1457 | GPR151 | G-protein coupled | 101 | 4 | 40 | 0 | 0.059405941 | 0.02970297 |
| P23416 | 1458 | GLRA2 | Glycine receptor s | 248 | 3 | 228 | 0.02016129 | 0.032258065 | 0.026209677 |
| Q96D42 | 1459 | HAVCR1 | Hepatitis A virus c | 274 | 1 | 274 | 0.255474453 | 0.259124088 | 0.25729927 |
| O00180 | 1460 | KCNK1 | Potassium channe | 89 | 4 | 61 | 0.011235955 | 0 | 0.005617978 |
| Q9UEF7 | 1461 | KL | Klotho (EC 3.2.1.3 | 947 | 1 | 947 | 0.017951426 | 0.012671595 | 0.01531151 |
| Q8N126 | 1462 | CADM3 | Cell adhesion mol | 305 | 1 | 305 | 0.072131148 | 0.006557377 | 0.039344262 |
| Q9HBB8 | 1463 | CDHR5 | Cadherin-related | 643 | 1 | 643 | 0.138413686 | 0.105754277 | 0.122083981 |
| Q15303 | 1464 | ERBB4 | Receptor tyrosine | 625 | 1 | 625 | 0.0448 | 0.0464 | 0.0456 |
| O60755 | 1465 | GALR3 | Galanin receptor t | 61 | 4 | 24 | 0.032786885 | 0.016393443 | 0.024590164 |
| Q99795 | 1466 | GPA33 | Cell surface A33 a | 213 | 1 | 213 | 0.051643192 | 0.009389671 | 0.030516432 |
| Q9NQS5 | 1467 | GPR84 | G-protein coupled | 64 | 4 | 25 | 0 | 0.0625 | 0.03125 |
| P49771 | 1468 | FLT3LG | Fms-related tyros | 157 | 1 | 157 | 0 | 0.044585987 | 0.022292994 |
| Q30201 | 1469 | HFE | Hereditary hemoc | 283 | 1 | 283 | 0.021201413 | 0.017667845 | 0.019434629 |
| P18505 | 1470 | GABRB1 | Gamma-aminobut | 219 | 1 | 219 | 0.02739726 | 0.01369863 | 0.020547945 |
| Q2M3T9 | 1471 | HYAL4 | Hyaluronidase-4 ( | 423 | 1 | 423 | 0.028368794 | 0.01891253 | 0.023640662 |
| O00219 | 1472 | HAS3 | Hyaluronan synth | 43 | 4 | 16 | 0 | 0 | 0 |
| Q13478 | 1473 | IL18R1 | Interleukin-18 rec | 307 | 1 | 307 | 0.055374593 | 0.03257329 | 0.043973941 |
| Q9NPH3 | 1474 | IL1RAP | Interleukin-1 rece | 346 | 1 | 346 | 0.046242775 | 0.01734104 | 0.031791908 |
| P43626 | 1475 | KIR2DL1 | Killer cell immuno | 223 | 1 | 223 | 0.071748879 | 0.049327354 | 0.060538117 |
| Q9H013 | 1476 | ADAM19 | Disintegrin and m | 496 | 1 | 496 | 0.024193548 | 0.02016129 | 0.022177419 |
| Q7Z7M1 | 1477 | ADGRD2 | Adhesion G-prote | 685 | 4 | 661 | 0.023357664 | 0.030656934 | 0.027007299 |
| Q7Z3S7 | 1478 | CACNA2D4 | Voltage-depende | 1095 | 1 | 1095 | 0.065753425 | 0.016438356 | 0.04109589 |

| | | | | | | | | | |
|---|---|---|---|---|---|---|---|---|---|
| P21854 | 1479 | CD72 | B-cell differentiati | 242 | 1 | 242 | 0 | 0.049586777 | 0.024793388 |
| P47870 | 1480 | GABRB2 | Gamma-aminobut | 218 | 1 | 218 | 0.018348624 | 0.013761468 | 0.016055046 |
| P14770 | 1481 | GP9 | Platelet glycoprot | 130 | 1 | 130 | 0.030769231 | 0.023076923 | 0.026923077 |
| Q16099 | 1482 | GRIK4 | Glutamate recepto | 683 | 2 | 524 | 0.026354319 | 0.020497804 | 0.023426061 |
| Q6UVM3 | 1483 | KCNT2 | Potassium channe | 35 | 4 | 16 | 0.028571429 | 0.057142857 | 0.042857143 |
| Q8N539 | 1484 | FIBCD1 | Fibrinogen C dom | 406 | 1 | 406 | 0.02955665 | 0.039408867 | 0.034482759 |
| Q96LA6 | 1485 | FCRL1 | Fc receptor-like pr | 290 | 1 | 290 | 0.075862069 | 0.010344828 | 0.043103448 |
| P21462 | 1486 | FPR1 | fMet-Leu-Phe rec | 102 | 4 | 42 | 0 | 0.098039216 | 0.049019608 |
| P32239 | 1487 | CCKBR | Gastrin/cholecyst | 123 | 4 | 56 | 0.048780488 | 0.081300813 | 0.06504065 |
| Q8IWB1 | 1488 | ITPRIP | Inositol 1,4,5-trisp | 67 | 1 | 67 | 0 | 0.029850746 | 0.014925373 |
| Q13304 | 1489 | GPR17 | Uracil nucleotide/ | 134 | 4 | 63 | 0.02238806 | 0.097014925 | 0.059701493 |
| Q8N6U8 | 1490 | GPR161 | G-protein coupled | 84 | 4 | 29 | 0 | 0.071428571 | 0.035714286 |
| P23415 | 1491 | GLRA1 | Glycine receptor s | 241 | 3 | 221 | 0.008298755 | 0.004149378 | 0.006224066 |
| A8MUP6 | 1492 | GSG1L2 | Germ cell-specific | 112 | 2 | 90 | 0.017857143 | 0.017857143 | 0.017857143 |
| P04439 | 1493 | HLA-A | HLA class I histoc | 283 | 1 | 283 | 0.024734982 | 0.014134276 | 0.019434629 |
| Q96F46 | 1494 | IL17RA | Interleukin-17 rec | 287 | 1 | 287 | 0.055749129 | 0.017421603 | 0.036585366 |
| P40189 | 1495 | IL6ST | Interleukin-6 rece | 596 | 1 | 596 | 0.120805369 | 0.02852349 | 0.07466443 |
| O14525 | 1496 | ASTN1 | Astrotactin-1 | 1030 | 2 | 899 | 0.019417476 | 0.021359223 | 0.02038835 |
| P06729 | 1497 | CD2 | T-cell surface ant | 184 | 1 | 184 | 0 | 0.02173913 | 0.010869565 |
| P28906 | 1498 | CD34 | Hematopoietic pro | 258 | 1 | 258 | 0.220930233 | 0.26744186 | 0.244186047 |
| O14638 | 1499 | ENPP3 | Ectonucleotide py | 844 | 1 | 844 | 0.039099526 | 0.026066351 | 0.032582938 |
| Q14332 | 1500 | FZD2 | Frizzled-2 (Fz-2) ( | 307 | 4 | 223 | 0.029315961 | 0.016286645 | 0.022801303 |
| Q9ULV1 | 1501 | FZD4 | Frizzled-4 (Fz-4) ( | 227 | 4 | 175 | 0.030837004 | 0.008810573 | 0.019823789 |
| Q9NSI5 | 1502 | IGSF5 | Immunoglobulin s | 265 | 1 | 265 | 0.037735849 | 0.022641509 | 0.030188679 |
| O71037 | 1503 | ERVK-19 | Endogenous retro | 542 | 1 | 542 | 0.040590406 | 0.016605166 | 0.028597786 |
| P06734 | 1504 | FCER2 | Low affinity immu | 273 | 1 | 273 | 0.036630037 | 0.036630037 | 0.036630037 |
| P25090 | 1505 | FPR2 | N-formyl peptide r | 103 | 4 | 42 | 0 | 0.077669903 | 0.038834951 |
| P24046 | 1506 | GABRR1 | Gamma-aminobut | 259 | 1 | 259 | 0.030888031 | 0.030888031 | 0.030888031 |
| Q95460 | 1507 | MR1 | Major histocompa | 279 | 1 | 279 | 0.021505376 | 0.007168459 | 0.014336918 |
| P16871 | 1508 | IL7R | Interleukin-7 rece | 218 | 1 | 218 | 0.041284404 | 0.02293578 | 0.032110092 |
| Q9NRM6 | 1509 | IL17RB | Interleukin-17 rec | 274 | 1 | 274 | 0.080291971 | 0.04379562 | 0.062043796 |
| P08648 | 1510 | ITGA5 | Integrin alpha-5 ( | 953 | 1 | 953 | 0.039874082 | 0.025183631 | 0.032528856 |
| Q8NC54 | 1511 | KCT2 | Keratinocyte-asso | 146 | 1 | 146 | 0.150684932 | 0.184931507 | 0.167808219 |
| Q96E93 | 1512 | KLRG1 | Killer cell lectin-lik | 135 | 1 | 135 | 0.02962963 | 0.022222222 | 0.025925926 |
| Q8TDW0 | 1513 | LRRC8C | Volume-regulated | 113 | 2 | 81 | 0.07079646 | 0.07079646 | 0.07079646 |
| P46095 | 1514 | GPR6 | G-protein coupled | 112 | 4 | 73 | 0 | 0.071428571 | 0.035714286 |
| Q14832 | 1515 | GRM3 | Metabotropic glut | 596 | 4 | 553 | 0.021812081 | 0.015100671 | 0.018456376 |
| P01893 | 1516 | HLA-H | Putative HLA clas | 283 | 1 | 283 | 0.028268551 | 0.007067138 | 0.017667845 |
| P30511 | 1517 | HLA-F | HLA class I histoc | 283 | 1 | 283 | 0.021201413 | 0.007067138 | 0.014134276 |
| Q9UMF0 | 1518 | ICAM5 | Intercellular adhe | 803 | 1 | 803 | 0.061021171 | 0.01618929 | 0.03860523 |
| P05556 | 1519 | ITGB1 | Integrin beta-1 (F | 707 | 1 | 707 | 0.025459689 | 0.043847242 | 0.034653465 |
| P20702 | 1520 | ITGAX | Integrin alpha-X ( | 1087 | 1 | 1087 | 0.032198712 | 0.022079117 | 0.027138914 |
| Q5VZ72 | 1521 | IZUMO3 | Izumo sperm-egg | 153 | 1 | 153 | 0.013071895 | 0.026143791 | 0.019607843 |
| P14616 | 1522 | INSRR | Insulin receptor-re | 174 | 1 | 174 | 0.034482759 | 0.022988506 | 0.028735632 |
| P43628 | 1523 | KIR2DL3 | Killer cell immuno | 223 | 1 | 223 | 0.071748879 | 0.040358744 | 0.056053812 |
| Q9NYG8 | 1524 | KCNK4 | Potassium channe | 86 | 4 | 62 | 0.046511628 | 0.023255814 | 0.034883721 |
| P19256 | 1525 | CD58 | Lymphocyte funct | 186 | 1 | 186 | 0.080645161 | 0.032258065 | 0.056451613 |
| Q9Y5Q5 | 1526 | CORIN | Atrial natriuretic p | 975 | 1 | 975 | 0.107692308 | 0.063589744 | 0.085641026 |
| P25024 | 1527 | CXCR1 | C-X-C chemokine | 96 | 4 | 38 | 0 | 0.072916667 | 0.036458333 |
| P32302 | 1528 | CXCR5 | C-X-C chemokine | 121 | 4 | 54 | 0 | 0.041322314 | 0.020661157 |
| Q6UWB1 | 1529 | IL27RA | Interleukin-27 rec | 483 | 1 | 483 | 0.051759834 | 0.020703934 | 0.036231884 |
| P08514 | 1530 | ITGA2B | Integrin alpha-IIb | 961 | 1 | 961 | 0.018730489 | 0.009365245 | 0.014047867 |
| Q9NPA1 | 1531 | KCNMB3 | Calcium-activated | 125 | 1 | 125 | 0.024 | 0.008 | 0.016 |
| Q8ND94 | 1532 | LRRN4CL | LRRN4 C-termina | 171 | 1 | 171 | 0.01754386 | 0.029239766 | 0.023391813 |
| Q15722 | 1533 | LTB4R | Leukotriene B4 re | 76 | 4 | 25 | 0.092105263 | 0.092105263 | 0.092105263 |
| Q96QE4 | 1534 | LRRC37B | Leucine-rich repe | 877 | 1 | 877 | 0.038768529 | 0.101482326 | 0.070125428 |
| Q6UXK5 | 1535 | LRRN1 | Leucine-rich repe | 605 | 1 | 605 | 0.024793388 | 0.034710744 | 0.029752066 |
| Q8N423 | 1536 | LILRB2 | Leukocyte immun | 439 | 1 | 439 | 0.061503417 | 0.043280182 | 0.0523918 |
| P01920 | 1537 | HLA-DQB1 | HLA class II histo | 197 | 1 | 197 | 0.035532995 | 0.010152284 | 0.02284264 |
| Q96AP7 | 1538 | ESAM | Endothelial cell-se | 218 | 1 | 218 | 0.041284404 | 0.02293578 | 0.032110092 |
| O75078 | 1539 | ADAM11 | Disintegrin and m | 508 | 1 | 508 | 0.023622047 | 0.037401575 | 0.030511811 |
| Q16581 | 1540 | C3AR1 | C3a anaphylatoxi | 232 | 4 | 179 | 0.047413793 | 0.060344828 | 0.05387931 |
| P22223 | 1541 | CDH3 | Cadherin-3 (Place | 546 | 1 | 546 | 0.049450549 | 0.003663004 | 0.026556777 |
| P27701 | 1542 | CD82 | CD82 antigen (C3 | 137 | 2 | 117 | 0.03649635 | 0.058394161 | 0.047445255 |
| P26842 | 1543 | CD27 | CD27 antigen (CD | 171 | 1 | 171 | 0.058479532 | 0.111111111 | 0.084795322 |
| O96005 | 1544 | CLPTM1 | Cleft lip and palat | 359 | 3 | 352 | 0.033426184 | 0.04735376 | 0.040389972 |
| Q99788 | 1545 | CMKLR1 | Chemokine-like re | 120 | 4 | 47 | 0.075 | 0.05 | 0.0625 |
| Q9BQT9 | 1546 | CLSTN3 | Calsyntenin-3 (Al | 827 | 1 | 827 | 0.019347037 | 0.019347037 | 0.019347037 |
| Q8N8Z6 | 1547 | DCBLD1 | Discoidin, CUB an | 424 | 1 | 424 | 0.054245283 | 0.058962264 | 0.056603774 |
| Q08345 | 1548 | DDR1 | Epithelial discoidi | 396 | 1 | 396 | 0.025252525 | 0.032828283 | 0.029040404 |
| O43155 | 1549 | FLRT2 | Leucine-rich repe | 505 | 1 | 505 | 0.069306931 | 0.077227723 | 0.073267327 |
| O00591 | 1550 | GABRP | Gamma-aminobut | 225 | 1 | 225 | 0.044444444 | 0.022222222 | 0.033333333 |
| Q9GZN0 | 1551 | GPR88 | Probable G-protei | 73 | 4 | 34 | 0.164383562 | 0.109589041 | 0.136986301 |
| Q96CH1 | 1552 | GPR146 | Probable G-protei | 89 | 4 | 29 | 0.033707865 | 0.02247191 | 0.028089888 |
| P47871 | 1553 | GCGR | Glucagon recepto | 164 | 4 | 110 | 0.054878049 | 0.024390244 | 0.039634146 |
| Q8NCS7 | 1554 | SLC44A5 | Choline transporte | 271 | 5 | 182 | 0.025830258 | 0.025830258 | 0.025830258 |
| P20036 | 1555 | HLA-DPA1 | HLA class II histo | 193 | 1 | 193 | 0.041450777 | 0.010362694 | 0.025906736 |
| P22794 | 1556 | EVI2A | Protein EVI2A (Ec | 102 | 1 | 102 | 0.176470588 | 0.058823529 | 0.117647059 |
| P36888 | 1557 | FLT3 | Receptor-type tyr | 516 | 1 | 516 | 0.040697674 | 0.025193798 | 0.032945736 |
| Q9P2B2 | 1558 | PTGFRN | Prostaglandin F2 | 806 | 1 | 806 | 0.02853598 | 0.014888337 | 0.021712159 |
| Q5SZK8 | 1559 | FREM2 | FRAS1-related ex | 3066 | 1 | 3066 | 0.027723418 | 0.023157208 | 0.025440313 |
| P48167 | 1560 | GLRB | Glycine receptor s | 255 | 2 | 245 | 0.015686275 | 0.019607843 | 0.017647059 |
| O43424 | 1561 | GRID2 | Glutamate recepto | 715 | 2 | 542 | 0.018181818 | 0.015384615 | 0.016783217 |
| O14672 | 1562 | ADAM10 | Disintegrin and m | 652 | 1 | 652 | 0.024539877 | 0.02607362 | 0.025306748 |
| A8MVZ5 | 1563 | BTNL10 | Butyrophilin-like p | 227 | 1 | 227 | 0.039647577 | 0.026431718 | 0.033039648 |
| P30988 | 1564 | CALCR | Calcitonin recepto | 179 | 4 | 127 | 0.039106145 | 0.033519553 | 0.036312849 |
| Q5JRM2 | 1565 | CXorf66 | Uncharacterized p | 27 | 1 | 27 | 0.148148148 | 0.074074074 | 0.111111111 |
| P24530 | 1566 | EDNRB | Endothelin recept | 123 | 4 | 74 | 0.146341463 | 0.105691057 | 0.12601626 |
| P14416 | 1567 | DRD2 | D(2) dopamine re | 78 | 4 | 36 | 0 | 0.064102564 | 0.032051282 |
| Q5MY95 | 1568 | ENTPD8 | Ectonucleoside tri | 441 | 1 | 441 | 0.045351474 | 0.031746032 | 0.038548753 |
| Q9UKQ2 | 1569 | ADAM28 | Disintegrin and m | 466 | 1 | 466 | 0.030042918 | 0.02360515 | 0.026824034 |
| P41180 | 1570 | CASR | Extracellular calci | 632 | 4 | 592 | 0.030063291 | 0.030063291 | 0.030063291 |
| O75976 | 1571 | CPD | Carboxypeptidase | 1267 | 1 | 1267 | 0.113654301 | 0.029992107 | 0.071823204 |
| P09693 | 1572 | CD3G | T-cell surface glyc | 93 | 1 | 93 | 0 | 0.021505376 | 0.010752688 |
| Q9H2A7 | 1573 | CXCL16 | C-X-C motif chem | 175 | 1 | 175 | 0 | 0.114285714 | 0.057142857 |
| B6SEH9 | 1574 | ERVV-2 | Endogenous retro | 356 | 2 | 299 | 0.146067416 | 0.025280899 | 0.085674157 |
| P29322 | 1575 | EPHA8 | Ephrin type-A rece | 514 | 1 | 514 | 0.031128405 | 0.027237354 | 0.029182879 |
| P12314 | 1576 | FCGR1A | High affinity immu | 276 | 1 | 276 | 0.061594203 | 0.028985507 | 0.045289855 |
| Q86XX4 | 1577 | FRAS1 | Extracellular matri | 3874 | 1 | 3874 | 0.030975736 | 0.027620031 | 0.029297883 |
| P25089 | 1578 | FPR3 | N-formyl peptide r | 103 | 4 | 42 | 0 | 0.058252427 | 0.029126214 |
| P31644 | 1579 | GABRA5 | Gamma-aminobut | 227 | 1 | 227 | 0.057268722 | 0.022026432 | 0.039647577 |
| A6NGU5 | 1580 | GGT3P | Putative glutathio | 541 | 1 | 541 | 0.042513863 | 0.014787431 | 0.028650647 |
| Q99678 | 1581 | GPR20 | G-protein coupled | 85 | 4 | 47 | 0 | 0.117647059 | 0.058823529 |
| Q14439 | 1582 | GPR176 | G-protein coupled | 90 | 4 | 41 | 0 | 0.088888889 | 0.044444444 |
| Q8IWK6 | 1583 | ADGRA3 | Adhesion G protei | 762 | 4 | 727 | 0.038057743 | 0.045931759 | 0.041994751 |
| Q8IZF4 | 1584 | ADGRG5 | Adhesion G-prote | 279 | 4 | 228 | 0.035842294 | 0.039426523 | 0.037634409 |
| Q5T601 | 1585 | ADGRF1 | Adhesion G-prote | 614 | 4 | 570 | 0.053745928 | 0.043973941 | 0.048859935 |
| P55064 | 1586 | AQP5 | Aquaporin-5 (AQF | 33 | 3 | 17 | 0.060606061 | 0.060606061 | 0.060606061 |
| Q9NY37 | 1587 | ASIC5 | Acid-sensing ion c | 376 | 1 | 376 | 0.034574468 | 0.039893617 | 0.037234043 |
| Q96FT7 | 1588 | ASIC4 | Acid-sensing ion c | 329 | 1 | 329 | 0.039513678 | 0.024316109 | 0.031914894 |
| Q6UXG8 | 1589 | BTNL9 | Butyrophilin-like p | 221 | 1 | 221 | 0.018099548 | 0.013574661 | 0.015837104 |
| Q9NPY3 | 1590 | CD93 | Complement comp | 556 | 1 | 556 | 0.059352518 | 0.091726619 | 0.075539568 |
| Q9HBT6 | 1591 | CDH20 | Cadherin-20 | 559 | 1 | 559 | 0.039355993 | 0.007155635 | 0.023255814 |
| P19397 | 1592 | CD53 | Leukocyte surface | 95 | 2 | 74 | 0.073684211 | 0.084210526 | 0.078947368 |

| | | | | | | | | | |
|---|---|---|---|---|---|---|---|---|---|
| Q13740 | 1593 | ALCAM | CD166 antigen (A | 499 | 1 | 499 | 0.054108216 | 0.016032064 | 0.03507014 |
| Q9NNX6 | 1594 | CD209 | CD209 antigen (C | 345 | 1 | 345 | 0.005797101 | 0.026086957 | 0.015942029 |
| O00548 | 1595 | DLL1 | Delta-like protein | 527 | 1 | 527 | 0.049335863 | 0.062618596 | 0.05597723 |
| P79483 | 1596 | HLA-DRB3 | HLA class II histo | 197 | 1 | 197 | 0.020304569 | 0.015228426 | 0.017766497 |
| P39086 | 1597 | GRIK1 | Glutamate recepto | 704 | 2 | 545 | 0.026988636 | 0.019886364 | 0.0234375 |
| P78536 | 1598 | ADAM17 | Disintegrin and m | 456 | 1 | 456 | 0.026315789 | 0.019736842 | 0.023026316 |
| Q9BYT9 | 1599 | ANO3 | Anoctamin-3 (Tra | 245 | 4 | 99 | 0.024489796 | 0.020408163 | 0.02244898 |
| P17342 | 1600 | NPR3 | Atrial natriuretic p | 454 | 1 | 454 | 0.022026432 | 0.013215859 | 0.017621145 |
| Q4KMQ2 | 1601 | ANO6 | Anoctamin-6 (Sm | 205 | 4 | 92 | 0.058536585 | 0.024390244 | 0.041463415 |
| Q01151 | 1602 | CD83 | CD83 antigen (hC | 124 | 1 | 124 | 0.072580645 | 0.024193548 | 0.048387097 |
| P15813 | 1603 | CD1D | Antigen-presentin | 281 | 1 | 281 | 0.049822064 | 0.017793594 | 0.033807829 |
| Q5QGZ9 | 1604 | CLEC12A | C-type lectin dom | 200 | 1 | 200 | 0 | 0.025 | 0.0125 |
| O00341 | 1605 | SLC1A7 | Excitatory amino a | 101 | 1 | 101 | 0.03960396 | 0.079207921 | 0.059405941 |
| P13762 | 1606 | HLA-DRB4 | HLA class II histo | 197 | 1 | 197 | 0.015228426 | 0.015228426 | 0.015228426 |
| Q9N2J8 | 1607 | 0 | HERV-H_2q24.1 p | 479 | 1 | 479 | 0 | 0.096033403 | 0.048016701 |
| P21860 | 1608 | ERBB3 | Receptor tyrosine | 623 | 1 | 623 | 0.028892456 | 0.027287319 | 0.028089888 |
| P21802 | 1609 | FGFR2 | Fibroblast growth | 355 | 1 | 355 | 0.064788732 | 0.030985915 | 0.047887324 |
| P31994 | 1610 | FCGR2B | Low affinity immu | 174 | 1 | 174 | 0.045977011 | 0.011494253 | 0.028735632 |
| P55899 | 1611 | FCGRT | IgG receptor FcRn | 273 | 1 | 273 | 0.032967033 | 0.003663004 | 0.018315018 |
| Q96P31 | 1612 | FCRL3 | Fc receptor-like pr | 555 | 1 | 555 | 0.043243243 | 0.016216216 | 0.02972973 |
| P28476 | 1613 | GABRR2 | Gamma-aminobut | 239 | 1 | 239 | 0.029288703 | 0.020920502 | 0.025104603 |
| P78363 | 1614 | ABCA4 | Retinal-specific pl | 932 | 2 | 603 | 0.032188841 | 0.017167382 | 0.024678112 |
| P05067 | 1615 | APP | Amyloid-beta prec | 683 | 1 | 683 | 0.036603221 | 0.061493411 | 0.049048316 |
| Q8IZF7 | 1616 | ADGRF2 | Adhesion G-prote | 500 | 4 | 450 | 0.026 | 0.038 | 0.032 |
| Q6IWH7 | 1617 | ANO7 | Anoctamin-7 (Dre | 234 | 4 | 104 | 0.008547009 | 0.02991453 | 0.019230769 |
| P35613 | 1618 | BSG | Basigin (5F7) (Co | 185 | 1 | 185 | 0.037837838 | 0.016216216 | 0.027027027 |
| P30411 | 1619 | BDKRB2 | B2 bradykinin rece | 111 | 4 | 59 | 0.063063063 | 0.117117117 | 0.09009009 |
| Q8IX05 | 1620 | CD302 | CD302 antigen (C | 145 | 1 | 145 | 0.013793103 | 0.006896552 | 0.010344828 |
| A0A0K2S4Q6 | 1621 | CD300H | Protein CD300H ( | 143 | 1 | 143 | 0 | 0 | 0 |
| Q96KA5 | 1622 | CLPTM1L | Cleft lip and palat | 259 | 3 | 252 | 0.038610039 | 0.011583012 | 0.025096525 |
| P34998 | 1623 | CRHR1 | Corticotropin-rele | 122 | 4 | 87 | 0.040983607 | 0.040983607 | 0.040983607 |
| P80370 | 1624 | DLK1 | Protein delta hom | 279 | 1 | 279 | 0.093189964 | 0.068100358 | 0.080645161 |
| P28222 | 1625 | HTR1B | 5-hydroxytryptam | 89 | 4 | 48 | 0 | 0.08988764 | 0.04494382 |
| P32297 | 1626 | CHRNA3 | Neuronal acetylch | 208 | 1 | 208 | 0.024038462 | 0.014423077 | 0.019230769 |
| Q53RT3 | 1627 | ASPRV1 | Retroviral-like asp | 266 | 1 | 266 | 0.011278195 | 0.011278195 | 0.011278195 |
| Q9NY47 | 1628 | CACNA2D2 | Voltage-depende | 1094 | 1 | 1094 | 0.02833638 | 0.02559415 | 0.026965265 |
| Q9Y6N8 | 1629 | CDH10 | Cadherin-10 (T2- | 558 | 1 | 558 | 0.043010753 | 0.007168459 | 0.025089606 |
| Q5ZPR3 | 1630 | CD276 | CD276 antigen (4 | 437 | 1 | 437 | 0.057208238 | 0.025171625 | 0.041189931 |
| Q9HCU0 | 1631 | CD248 | Endosialin (Tumo | 669 | 1 | 669 | 0.071748879 | 0.098654709 | 0.085201794 |
| P51679 | 1632 | CCR4 | C-C chemokine re | 96 | 4 | 38 | 0 | 0.0625 | 0.03125 |
| P51684 | 1633 | CCR6 | C-C chemokine re | 113 | 4 | 46 | 0 | 0.03539823 | 0.017699115 |
| O95196 | 1634 | CSPG5 | Chondroitin sulfat | 392 | 1 | 392 | 0.066326531 | 0.125 | 0.095663265 |
| P00533 | 1635 | EGFR | Epidermal growth | 620 | 1 | 620 | 0.032258065 | 0.017741935 | 0.025 |
| Q9H6D8 | 1636 | FNDC4 | Fibronectin type II | 122 | 1 | 122 | 0.06557377 | 0.081967213 | 0.073770492 |
| O75084 | 1637 | FZD7 | Frizzled-7 (Fz-7) ( | 307 | 4 | 223 | 0.029315961 | 0.013029316 | 0.021172638 |
| Q13467 | 1638 | FZD5 | Frizzled-5 (Fz-5) ( | 285 | 4 | 211 | 0.042105263 | 0.010526316 | 0.026315789 |
| O75899 | 1639 | GABBR2 | Gamma-aminobut | 490 | 4 | 441 | 0.053061224 | 0.016326531 | 0.034693878 |
| P08910 | 1640 | ABHD2 | Monoacylglycerol | 394 | 1 | 394 | 0.015228426 | 0.005076142 | 0.010152284 |
| O00400 | 1641 | SLC33A1 | Acetyl-coenzyme | 92 | 6 | 22 | 0.02173913 | 0 | 0.010869565 |
| Q05901 | 1642 | CHRNB3 | Neuronal acetylch | 207 | 1 | 207 | 0.048309179 | 0 | 0.024154589 |
| P07510 | 1643 | CHRNG | Acetylcholine rece | 217 | 1 | 217 | 0.013824885 | 0 | 0.006912442 |
| Q9Y653 | 1644 | ADGRG1 | Adhesion G-prote | 423 | 4 | 376 | 0.052009456 | 0.030732861 | 0.041371158 |
| Q8TC27 | 1645 | ADAM32 | Disintegrin and m | 507 | 1 | 507 | 0.009861933 | 0.029585799 | 0.019723866 |
| Q8IZF3 | 1646 | ADGRF4 | Adhesion G protei | 437 | 4 | 384 | 0.04576659 | 0.054919908 | 0.050343249 |
| Q13145 | 1647 | BAMBI | BMP and activin m | 131 | 1 | 131 | 0.091603053 | 0.076335878 | 0.083969466 |
| P10747 | 1648 | CD28 | T-cell-specific sur | 133 | 1 | 133 | 0 | 0.007518797 | 0.003759398 |
| Q9BYE9 | 1649 | CDHR2 | Cadherin-related | 1133 | 1 | 1133 | 0.044130627 | 0.000882613 | 0.02250662 |
| Q6P4Q7 | 1650 | CNNM4 | Metal transporter | 184 | 2 | 177 | 0.032608696 | 0.005434783 | 0.019021739 |
| Q9H8M5 | 1651 | CNNM2 | Metal transporter | 257 | 2 | 249 | 0.046692607 | 0.023346304 | 0.035019455 |
| P16422 | 1652 | EPCAM | Epithelial cell adh | 241 | 1 | 241 | 0.024896266 | 0.008298755 | 0.01659751 |
| P29317 | 1653 | EPHA2 | Ephrin type-A rece | 513 | 1 | 513 | 0.040935673 | 0.033138402 | 0.037037037 |
| P36269 | 1654 | GGT5 | Glutathione hydro | 556 | 1 | 556 | 0.037769784 | 0.032374101 | 0.035071942 |
| Q6ZMI3 | 1655 | GLDN | Gliomedin [Cleave | 511 | 1 | 511 | 0.029354207 | 0.054794521 | 0.042074364 |
| P48058 | 1656 | GRIA4 | Glutamate recepto | 696 | 2 | 522 | 0.034482759 | 0.017241379 | 0.025862069 |
| Q8TBP5 | 1657 | FAM174A | Membrane protei | 93 | 1 | 93 | 0.043010753 | 0.086021505 | 0.064516129 |
| P19235 | 1658 | EPOR | Erythropoietin rec | 225 | 1 | 225 | 0.022222222 | 0.013333333 | 0.017777778 |
| P24071 | 1659 | FCAR | Immunoglobulin a | 205 | 1 | 205 | 0.034146341 | 0.024390244 | 0.029268293 |
| Q2I0M4 | 1660 | LRRC26 | Leucine-rich repe | 234 | 1 | 234 | 0.025641026 | 0.042735043 | 0.034188034 |
| Q99705 | 1661 | MCHR1 | Melanin-concentr | 165 | 4 | 112 | 0.16969697 | 0.109090909 | 0.139393939 |
| P08183 | 1662 | ABCB1 | ATP-dependent tr | 105 | 5 | 48 | 0 | 0.028571429 | 0.014285714 |
| Q07001 | 1663 | CHRND | Acetylcholine rece | 223 | 1 | 223 | 0.053811659 | 0.013452915 | 0.033632287 |
| A5X5Y0 | 1664 | HTR3E | 5-hydroxytryptami | 226 | 3 | 222 | 0 | 0.017699115 | 0.008849558 |
| P30532 | 1665 | CHRNA5 | Neuronal acetylch | 247 | 2 | 231 | 0.052631579 | 0.024291498 | 0.038461538 |
| A1A5B4 | 1666 | ANO9 | Anoctamin-9 (Tra | 248 | 4 | 107 | 0.02016129 | 0.044354839 | 0.032258065 |
| P11049 | 1667 | CD37 | Leukocyte antiger | 149 | 2 | 129 | 0.013422819 | 0.067114094 | 0.040268456 |
| Q08722 | 1668 | CD47 | Leukocyte surface | 142 | 3 | 122 | 0.154929577 | 0.042253521 | 0.098591549 |
| P06126 | 1669 | CD1A | T-cell surface glyc | 283 | 1 | 283 | 0 | 0.010600707 | 0.005300353 |
| Q9H5V8 | 1670 | CDCP1 | CUB domain-cont | 637 | 1 | 637 | 0.050235479 | 0.021978022 | 0.03610675 |
| Q9H9P2 | 1671 | CHODL | Chondrolectin (Tr | 194 | 1 | 194 | 0.025773196 | 0.020618557 | 0.023195876 |
| P43627 | 1672 | KIR2DL2 | Killer cell immuno | 223 | 1 | 223 | 0.058295964 | 0.040358744 | 0.049327354 |
| Q5JUK3 | 1673 | KCNT1 | Potassium channe | 49 | 4 | 36 | 0.06122449 | 0.06122449 | 0.06122449 |
| P22459 | 1674 | KCNA4 | Potassium voltage | 76 | 4 | 43 | 0.065789474 | 0.013157895 | 0.039473684 |
| Q16558 | 1675 | KCNMB1 | Calcium-activated | 117 | 1 | 117 | 0.025641026 | 0.025641026 | 0.025641026 |
| Q6UWH4 | 1676 | GASK1B | Golgi-associated | 463 | 1 | 463 | 0.017278618 | 0.036717063 | 0.02699784 |
| O95800 | 1677 | GPR75 | Probable G-protei | 90 | 4 | 45 | 0.088888889 | 0.111111111 | 0.1 |
| Q5T6X5 | 1678 | GPRC6A | G-protein coupled | 619 | 4 | 575 | 0 | 0.027463651 | 0.013731826 |
| Q5VW38 | 1679 | GPR107 | Protein GPR107 ( | 261 | 4 | 223 | 0.030651341 | 0.053639847 | 0.042145594 |
| P25021 | 1680 | HRH2 | Histamine H2 rece | 59 | 4 | 21 | 0.101694915 | 0.050847458 | 0.076271186 |
| P13598 | 1681 | ICAM2 | Intercellular adhe | 198 | 1 | 198 | 0.065656566 | 0.035353535 | 0.050505051 |
| O75578 | 1682 | ITGA10 | Integrin alpha-10 | 1099 | 1 | 1099 | 0.023657871 | 0.01910828 | 0.021383076 |
| Q86YT9 | 1683 | JAML | Junctional adhesio | 255 | 1 | 255 | 0.015686275 | 0.007843137 | 0.011764706 |
| Q9Y624 | 1684 | F11R | Junctional adhesio | 210 | 1 | 210 | 0.066666667 | 0.004761905 | 0.035714286 |
| Q14954 | 1685 | KIR2DS1 | Killer cell immuno | 223 | 1 | 223 | 0.080717489 | 0.040358744 | 0.060538117 |
| O75019 | 1686 | LILRA1 | Leukocyte immun | 444 | 1 | 444 | 0.042792793 | 0.040540541 | 0.041666667 |
| Q5NUL3 | 1687 | FFAR4 | Free fatty acid rec | 88 | 4 | 44 | 0.045454545 | 0.056818182 | 0.051136364 |
| Q6DN72 | 1688 | FCRL6 | Fc receptor-like pr | 287 | 1 | 287 | 0.031358885 | 0.010452962 | 0.020905923 |
| Q8TDY8 | 1689 | IGDCC4 | Immunoglobulin s | 932 | 1 | 932 | 0.0472103 | 0.025751073 | 0.036480687 |
| Q99665 | 1690 | IL12RB2 | Interleukin-12 rec | 598 | 1 | 598 | 0.040133779 | 0.015050167 | 0.027591973 |
| Q9UHF4 | 1691 | IL20RA | Interleukin-20 rec | 220 | 1 | 220 | 0.045454545 | 0.022727273 | 0.034090909 |
| Q14773 | 1692 | ICAM4 | Intercellular adhe | 217 | 1 | 217 | 0.064516129 | 0.055299539 | 0.059907834 |
| P98164 | 1693 | LRP2 | Low-density lipop | 4397 | 1 | 4397 | 0 | 0.040254719 | 0.02012736 |
| Q07954 | 1694 | LRP1 | Prolow-density lip | 4399 | 1 | 4399 | 0.028870198 | 0.042055013 | 0.035462605 |
| Q8N967 | 1695 | LRTM2 | Leucine-rich repe | 274 | 1 | 274 | 0.04379562 | 0.032846715 | 0.038321168 |
| P29320 | 1696 | EPHA3 | Ephrin type-A rece | 520 | 1 | 520 | 0.030769231 | 0.025 | 0.027884615 |
| P11362 | 1697 | FGFR1 | Fibroblast growth | 354 | 1 | 354 | 0.141242938 | 0.042372881 | 0.09180791 |
| Q9HCN6 | 1698 | GP6 | Platelet glycoprot | 246 | 1 | 246 | 0.203252033 | 0.052845528 | 0.12804878 |
| P06028 | 1699 | GYPB | Glycophorin-B (P/ | 39 | 1 | 39 | 0.307692308 | 0.179487179 | 0.243589744 |
| O75473 | 1700 | LGR5 | Leucine-rich repe | 593 | 4 | 539 | 0.02698145 | 0.016863406 | 0.021922428 |
| Q12918 | 1701 | KLRB1 | Killer cell lectin-lik | 158 | 1 | 158 | 0.044303797 | 0.018987342 | 0.03164557 |
| Q9H8J5 | 1702 | MANSC1 | MANSC domain-c | 358 | 1 | 358 | 0.131284916 | 0.223463687 | 0.177374302 |
| P25929 | 1703 | NPY1R | Neuropeptide Y re | 113 | 4 | 43 | 0.053097345 | 0.053097345 | 0.053097345 |
| Q16653 | 1704 | MOG | Myelin-oligodend | 139 | 2 | 124 | 0.014388489 | 0 | 0.007194245 |
| Q01113 | 1705 | IL9R | Interleukin-9 rece | 229 | 1 | 229 | 0.034934498 | 0.017467249 | 0.026200873 |
| O75144 | 1706 | ICOSLG | ICOS ligand (B7 h | 237 | 1 | 237 | 0.050632911 | 0.021097046 | 0.035864979 |

| | | | | | | | | | |
|---|---|---|---|---|---|---|---|---|---|
| P17301 | 1707 | ITGA2 | Integrin alpha-2 ( | 1102 | 1 | 1102 | 0.033575318 | 0.020871143 | 0.02722323 |
| P43629 | 1708 | KIR3DL1 | Killer cell immuno | 318 | 1 | 318 | 0.072327044 | 0.034591195 | 0.053459119 |
| Q86YD5 | 1709 | LDLRAD3 | Low-density lipop | 155 | 1 | 155 | 0 | 0.070967742 | 0.035483871 |
| Q86VH5 | 1710 | LRRTM3 | Leucine-rich repe | 388 | 1 | 388 | 0.015463918 | 0.012886598 | 0.014175258 |
| Q9NT99 | 1711 | LRRC4B | Leucine-rich repe | 540 | 1 | 540 | 0.088888889 | 0.064814815 | 0.076851852 |
| Q9ULH4 | 1712 | LRFN2 | Leucine-rich repe | 513 | 1 | 513 | 0.038986355 | 0.046783626 | 0.04288499 |
| P33032 | 1713 | MC5R | Melanocortin rece | 65 | 4 | 36 | 0.061538462 | 0.092307692 | 0.076923077 |
| P49146 | 1714 | NPY2R | Neuropeptide Y re | 109 | 4 | 50 | 0.036697248 | 0.036697248 | 0.036697248 |
| P16109 | 1715 | SELP | P-selectin (CD62 | 729 | 1 | 729 | 0 | 0.061728395 | 0.030864198 |
| Q6ZRH7 | 1716 | CATSPERG | Cation channel sp | 1030 | 1 | 1030 | 0.021359223 | 0.008737864 | 0.015048544 |
| Q96PD2 | 1717 | DCBLD2 | Discoidin, CUB an | 461 | 1 | 461 | 0.060737527 | 0.052060738 | 0.056399132 |
| O60469 | 1718 | DSCAM | Down syndrome c | 1577 | 1 | 1577 | 0.051363348 | 0.017121116 | 0.034242232 |
| P0C7U0 | 1719 | ELFN1 | Protein ELFN1 (E | 390 | 1 | 390 | 0.056410256 | 0.056410256 | 0.056410256 |
| Q9ULD8 | 1720 | KCNH3 | Potassium voltage | 90 | 4 | 70 | 0.055555556 | 0.033333333 | 0.044444444 |
| Q92633 | 1721 | LPAR1 | Lysophosphatidic | 94 | 4 | 49 | 0.106382979 | 0.095744681 | 0.10106383 |
| Q7L985 | 1722 | LINGO2 | Leucine-rich repe | 517 | 1 | 517 | 0.046421663 | 0.042553191 | 0.044487427 |
| Q96FE5 | 1723 | LINGO1 | Leucine-rich repe | 519 | 1 | 519 | 0.028901734 | 0.034682081 | 0.031791908 |
| Q29980 | 1724 | MICB | MHC class I polyp | 286 | 1 | 286 | 0.097902098 | 0.031468531 | 0.064685315 |
| O00574 | 1725 | CXCR6 | C-X-C chemokine | 81 | 4 | 31 | 0 | 0.037037037 | 0.018518519 |
| O15303 | 1726 | GRM6 | Metabotropic glut | 611 | 4 | 560 | 0.019639935 | 0.024549918 | 0.022094926 |
| Q9BXC0 | 1727 | HCAR1 | Hydroxycarboxylic | 87 | 4 | 30 | 0.011494253 | 0.011494253 | 0.011494253 |
| Q29983 | 1728 | MICA | MHC class I polyp | 283 | 1 | 283 | 0.049469965 | 0.038869258 | 0.044169611 |
| P33527 | 1729 | ABCC1 | Multidrug resistar | 251 | 8 | 114 | 0.03187251 | 0.027888446 | 0.029880478 |
| Q5T197 | 1730 | DCST1 | E3 ubiquitin-prote | 312 | 3 | 235 | 0.028846154 | 0.016025641 | 0.022435897 |
| Q9Y2G8 | 1731 | DNAJC16 | DnaJ homolog sub | 225 | 1 | 225 | 0.022222222 | 0.053333333 | 0.037777778 |
| P43004 | 1732 | SLC1A2 | Excitatory amino a | 158 | 5 | 92 | 0.025316456 | 0.037974684 | 0.03164557 |
| Q9UNE0 | 1733 | EDAR | Tumor necrosis fa | 160 | 1 | 160 | 0.05625 | 0.0375 | 0.046875 |
| Q86SJ6 | 1734 | DSG4 | Desmoglein-4 (Ca | 581 | 1 | 581 | 0.030981067 | 0.003442341 | 0.017211704 |
| Q902F9 | 1735 | HERVK_113 | Endogenous retro | 542 | 1 | 542 | 0.044280443 | 0.018450185 | 0.031365314 |
| P01589 | 1736 | IL2RA | Interleukin-2 rece | 218 | 1 | 218 | 0.077981651 | 0.114678899 | 0.096330275 |
| P38570 | 1737 | ITGAE | Integrin alpha-E ( | 1105 | 1 | 1105 | 0.026244344 | 0.030769231 | 0.028506787 |
| D3W0D1 | 1738 | KLRF2 | Killer cell lectin-lik | 155 | 1 | 155 | 0.038709677 | 0.019354839 | 0.029032258 |
| Q96PL5 | 1739 | ERMAP | Erythroid membra | 125 | 1 | 125 | 0.016 | 0.024 | 0.02 |
| P54760 | 1740 | EPHB4 | Ephrin type-B rece | 523 | 1 | 523 | 0.04206501 | 0.028680688 | 0.035372849 |
| P22455 | 1741 | FGFR4 | Fibroblast growth | 347 | 1 | 347 | 0.040345821 | 0.031700288 | 0.036023055 |
| O60353 | 1742 | FZD6 | Frizzled-6 (Fz-6) | 270 | 4 | 182 | 0.025925926 | 0.02962963 | 0.027777778 |
| O43603 | 1743 | GALR2 | Galanin receptor t | 69 | 4 | 27 | 0.072463768 | 0.072463768 | 0.072463768 |
| P28472 | 1744 | GABRB3 | Gamma-aminobut | 229 | 2 | 219 | 0.017467249 | 0.013100437 | 0.015283843 |
| P78334 | 1745 | GABRE | Gamma-aminobut | 230 | 1 | 230 | 0.017391304 | 0.043478261 | 0.030434783 |
| P48546 | 1746 | GIPR | Gastric inhibitory | 171 | 4 | 116 | 0.035087719 | 0.01754386 | 0.026315789 |
| P51810 | 1747 | GPR143 | G-protein coupled | 93 | 4 | 27 | 0 | 0.010752688 | 0.005376344 |
| P42262 | 1748 | GRIA2 | Glutamate recept | 692 | 2 | 518 | 0.030346821 | 0.01734104 | 0.023843931 |
| P05981 | 1749 | HPN | Serine protease h | 372 | 1 | 372 | 0.016129032 | 0.01344086 | 0.014784946 |
| P48549 | 1750 | KCNJ3 | G protein-activate | 31 | 2 | 23 | 0.032258065 | 0 | 0.016129032 |
| Q9HBW0 | 1751 | LPAR2 | Lysophosphatidic | 79 | 4 | 32 | 0.025316456 | 0.025316456 | 0.025316456 |
| Q6PI73 | 1752 | LILRA6 | Leukocyte immun | 423 | 1 | 423 | 0.052009456 | 0.028368794 | 0.040189125 |
| Q9GZU1 | 1753 | MCOLN1 | Mucolipin-1 (ML1 | 241 | 4 | 211 | 0.01659751 | 0.020746888 | 0.018672199 |
| Q14831 | 1754 | GRM7 | Metabotropic glut | 603 | 4 | 555 | 0.018242123 | 0.018242123 | 0.018242123 |
| O95528 | 1755 | SLC2A10 | Solute carrier fam | 145 | 6 | 94 | 0.055172414 | 0.062068966 | 0.05862069 |
| Q8TD20 | 1756 | SLC2A12 | Solute carrier fam | 141 | 5 | 99 | 0.035460993 | 0.042553191 | 0.039007092 |
| P78552 | 1757 | IL13RA1 | Interleukin-13 rec | 321 | 1 | 321 | 0.056074766 | 0.037383178 | 0.046728972 |
| Q5VWK5 | 1758 | IL23R | Interleukin-23 rec | 331 | 1 | 331 | 0.048338369 | 0.024169184 | 0.036253776 |
| Q8IZA0 | 1759 | KIAA0319L | Dyslexia-associat | 881 | 1 | 881 | 0.052213394 | 0.085130533 | 0.068671964 |
| P56199 | 1760 | ITGA1 | Integrin alpha-1 ( | 1112 | 1 | 1112 | 0.048561151 | 0.033273381 | 0.040917266 |
| P78504 | 1761 | JAG1 | Protein jagged-1 | 1033 | 1 | 1033 | 0.031945789 | 0.070667957 | 0.051306873 |
| Q96J84 | 1762 | KIRREL1 | Kin of IRRE-like pr | 482 | 1 | 482 | 0.049792531 | 0.010373444 | 0.030082988 |
| Q99706 | 1763 | KIR2DL4 | Killer cell immuno | 220 | 1 | 220 | 0.072727273 | 0.036363636 | 0.054545455 |
| Q13241 | 1764 | KLRD1 | Natural killer cells | 147 | 1 | 147 | 0.020408163 | 0.020408163 | 0.020408163 |
| Q8N149 | 1765 | LILRA2 | Leukocyte immun | 425 | 1 | 425 | 0.117647059 | 0.051764706 | 0.084705882 |
| Q969V1 | 1766 | MCHR2 | Melanin-concentr | 89 | 4 | 38 | 0.033707865 | 0.06741573 | 0.050561798 |
| P35410 | 1767 | MAS1L | Mas-related G-pr | 116 | 4 | 76 | 0 | 0.034482759 | 0.017241379 |
| Q02297 | 1768 | NRG1 | Pro-neuregulin-1, | 222 | 1 | 222 | 0 | 0.103603604 | 0.051801802 |
| P35367 | 1769 | HRH1 | Histamine H1 rece | 80 | 4 | 28 | 0.0375 | 0.025 | 0.03125 |
| Q8NFR9 | 1770 | IL17RE | Interleukin-17 rec | 430 | 1 | 430 | 0.025581395 | 0.048837209 | 0.037209302 |
| P14784 | 1771 | IL2RB | Interleukin-2 rece | 213 | 1 | 213 | 0.023474178 | 0.018779343 | 0.021126761 |
| P08887 | 1772 | IL6R | Interleukin-6 rece | 345 | 1 | 345 | 0.069565217 | 0.037681159 | 0.053623188 |
| Q9Y6W8 | 1773 | ICOS | Inducible T-cell c | 119 | 1 | 119 | 0.033613445 | 0.025210084 | 0.029411765 |
| Q9BX67 | 1774 | JAM3 | Junctional adhesi | 209 | 1 | 209 | 0.052631579 | 0.004784689 | 0.028708134 |
| Q96JA1 | 1775 | LRIG1 | Leucine-rich repe | 759 | 1 | 759 | 0.048748353 | 0.007905138 | 0.028326746 |
| P51512 | 1776 | MMP16 | Matrix metallopro | 444 | 1 | 444 | 0.022522523 | 0.015765766 | 0.019144144 |
| O00533 | 1777 | CHL1 | Neural cell adhesi | 1057 | 1 | 1057 | 0.046357616 | 0.022705771 | 0.034531693 |
| Q9Y5N1 | 1778 | HRH3 | Histamine H3 rece | 86 | 4 | 38 | 0.023255814 | 0.011627907 | 0.01744186 |
| P10321 | 1779 | HLA-C | HLA class I histoc | 283 | 1 | 283 | 0.028268551 | 0.010600707 | 0.019434629 |
| P98153 | 1780 | DGCR2 | Integral membran | 328 | 1 | 328 | 0.012195122 | 0.076219512 | 0.044207317 |
| Q13683 | 1781 | ITGA7 | Integrin alpha-7 [ | 1048 | 1 | 1048 | 0.016221374 | 0.015267176 | 0.015744275 |
| P16144 | 1782 | ITGB4 | Integrin beta-4 (G | 682 | 1 | 682 | 0.030791789 | 0.026392962 | 0.028592375 |
| Q8N109 | 1783 | KIR2DL5A | Killer cell immuno | 216 | 1 | 216 | 0.078703704 | 0.069444444 | 0.074074074 |
| A6NHS7 | 1784 | MANSC4 | MANSC domain-c | 259 | 1 | 259 | 0.1003861 | 0.146718147 | 0.123552124 |
| Q96KJ4 | 1785 | MSLNL | Mesothelin-like pr | 602 | 1 | 602 | 0.049833887 | 0.066445183 | 0.058139535 |
| Q9HC58 | 1786 | SLC24A3 | Sodium/potassiur | 91 | 6 | 62 | 0.032967033 | 0.054945055 | 0.043956044 |
| P31995 | 1787 | FCGR2C | Low affinity immu | 180 | 1 | 180 | 0.055555556 | 0.011111111 | 0.033333333 |
| Q9H461 | 1788 | FZD8 | Frizzled-8 (Fz-8) | 361 | 4 | 247 | 0.024930748 | 0.033240997 | 0.029085873 |
| Q04609 | 1789 | FOLH1 | Glutamate carbox | 706 | 1 | 706 | 0.028328612 | 0.016997167 | 0.02266289 |
| P18507 | 1790 | GABRG2 | Gamma-aminobut | 233 | 1 | 233 | 0.030042918 | 0.021459227 | 0.025751073 |
| Q99928 | 1791 | GABRG3 | Gamma-aminobut | 236 | 1 | 236 | 0.029661017 | 0.012711864 | 0.021186441 |
| Q99680 | 1792 | GPR22 | G-protein coupled | 94 | 4 | 44 | 0 | 0.042553191 | 0.021276596 |
| Q9ULK0 | 1793 | GRID1 | Glutamate recept | 712 | 2 | 541 | 0.019662921 | 0.018258427 | 0.018960674 |
| Q9P1Z3 | 1794 | HCN3 | Potassium/sodiur | 44 | 4 | 22 | 0.022727273 | 0 | 0.011363636 |
| Q9H252 | 1795 | KCNH6 | Potassium voltage | 71 | 4 | 43 | 0.056338028 | 0.014084507 | 0.035211268 |
| Q8TDN2 | 1796 | KCNV2 | Potassium voltage | 117 | 4 | 84 | 0.034188034 | 0 | 0.017094017 |
| P32004 | 1797 | L1CAM | Neural cell adhesi | 1100 | 1 | 1100 | 0.040909091 | 0.026363636 | 0.033636364 |
| Q5SZI1 | 1798 | LDLRAD2 | Low-density lipop | 207 | 1 | 207 | 0 | 0.072463768 | 0.036231884 |
| Q9NUN5 | 1799 | LMBRD1 | Lysosomal cobala | 152 | 5 | 56 | 0.059210526 | 0.065789474 | 0.0625 |
| Q9P244 | 1800 | LRFN1 | Leucine-rich repe | 504 | 1 | 504 | 0.041666667 | 0.029761905 | 0.035714286 |
| A6NI73 | 1801 | LILRA5 | Leukocyte immun | 226 | 1 | 226 | 0.061946903 | 0.017699115 | 0.039823009 |
| Q8TDD5 | 1802 | MCOLN3 | Mucolipin-3 (Trar | 224 | 4 | 199 | 0.017857143 | 0.013392857 | 0.015625 |
| O14944 | 1803 | EREG | Proepiregulin [Cle | 59 | 1 | 59 | 0 | 0.016949153 | 0.008474576 |
| F2Z333 | 1804 | FNDC10 | Fibronectin type II | 161 | 1 | 161 | 0.031055901 | 0.031055901 | 0.031055901 |
| O15197 | 1805 | EPHB6 | Ephrin type-B rece | 562 | 1 | 562 | 0.056939502 | 0.049822064 | 0.053380783 |
| P22607 | 1806 | FGFR3 | Fibroblast growth | 352 | 1 | 352 | 0.045454545 | 0.03125 | 0.038352273 |
| P51674 | 1807 | GPM6A | Neuronal membra | 104 | 2 | 64 | 0.028846154 | 0.019230769 | 0.024038462 |
| P30968 | 1808 | GNRHR | Gonadotropin-rele | 86 | 4 | 37 | 0.081395349 | 0.058139535 | 0.069767442 |
| Q7Z602 | 1809 | GPR141 | Probable G-protei | 86 | 4 | 30 | 0.11627907 | 0.034883721 | 0.075581395 |
| Q5T848 | 1810 | GPR158 | Probable G-protei | 440 | 4 | 393 | 0.034090909 | 0.047727273 | 0.040909091 |
| P16150 | 1811 | SPN | Leukosialin (GPL1 | 233 | 1 | 233 | 0.326180258 | 0.39055794 | 0.358369099 |
| Q86VH4 | 1812 | LRRTM4 | Leucine-rich repe | 393 | 1 | 393 | 0.027989822 | 0.03562341 | 0.031806616 |
| Q9H9K5 | 1813 | ERVMER34-1 | Endogenous retro | 461 | 1 | 461 | 0.069414317 | 0.065075922 | 0.067245119 |
| Q16478 | 1814 | GRIK5 | Glutamate recept | 688 | 2 | 529 | 0.039244186 | 0.024709302 | 0.031976744 |
| Q6MZM0 | 1815 | HEPHL1 | Ferroxidase HEPH | 1089 | 1 | 1089 | 0.029384757 | 0.012855831 | 0.021120294 |
| P57087 | 1816 | JAM2 | Junctional adhesi | 209 | 1 | 209 | 0.04784689 | 0.014354067 | 0.031100478 |
| Q12809 | 1817 | KCNH2 | Potassium voltage | 75 | 4 | 42 | 0.026666667 | 0.013333333 | 0.02 |
| O75197 | 1818 | LRP5 | Low-density lipop | 1352 | 1 | 1352 | 0 | 0.014792899 | 0.00739645 |
| Q96MC6 | 1819 | MFSD14A | Hippocampus abu | 159 | 7 | 41 | 0.037735849 | 0.037735849 | 0.037735849 |
| A8MWY0 | 1820 | ELAPOR2 | Endosome/lysoso | 881 | 1 | 881 | 0.05107832 | 0.082860386 | 0.066969353 |

| | | | | | | | | |
|---|---|---|---|---|---|---|---|---|
| A8MVW0 | 1821 | FAM171A2 | Protein FAM171A | 285 | 1 | 285 | 0.077192982 | 0.021052632 | 0.049122807 |
| Q5VUB5 | 1822 | FAM171A1 | Protein FAM171A | 281 | 1 | 281 | 0.060498221 | 0.010676157 | 0.035587189 |
| Q9Y5Y0 | 1823 | FLVCR1 | Feline leukemia vi | 63 | 6 | 19 | 0.031746032 | 0.015873016 | 0.023809524 |
| Q9UF33 | 1824 | EPHA6 | Ephrin type-A rece | 527 | 1 | 527 | 0.028462998 | 0.020872865 | 0.024667932 |
| P11166 | 1825 | SLC2A1 | Solute carrier fam | 74 | 6 | 32 | 0.054054054 | 0.027027027 | 0.040540541 |
| Q6P531 | 1826 | GGT6 | Glutathione hydro | 417 | 1 | 417 | 0.055155875 | 0.033573141 | 0.044364508 |
| Q8TDV2 | 1827 | GPR148 | Probable G-protei | 116 | 4 | 50 | 0.051724138 | 0.043103448 | 0.047413793 |
| Q9BYW1 | 1828 | SLC2A11 | Solute carrier fam | 70 | 6 | 28 | 0.042857143 | 0.028571429 | 0.035714286 |
| P04233 | 1829 | CD74 | HLA class II histoc | 223 | 1 | 223 | 0.031390135 | 0.080717489 | 0.056053812 |
| Q9BQS7 | 1830 | HEPH | Hephaestin (EC 1. | 1086 | 1 | 1086 | 0.057090239 | 0.011970534 | 0.034530387 |
| Q6UWM7 | 1831 | LCTL | Lactase-like prote | 518 | 1 | 518 | 0.017374517 | 0.011583012 | 0.014478764 |
| Q7Z4F1 | 1832 | LRP10 | Low-density lipop | 423 | 1 | 423 | 0 | 0.085106383 | 0.042553191 |
| Q6UY18 | 1833 | LINGO4 | Leucine-rich repe | 504 | 1 | 504 | 0.031746032 | 0.023809524 | 0.027777778 |
| Q16819 | 1834 | MEP1A | Meprin A subunit | 646 | 1 | 646 | 0.037151703 | 0.041795666 | 0.039473684 |
| Q9BY79 | 1835 | MFRP | Membrane frizzled | 488 | 1 | 488 | 0.06352459 | 0.049180328 | 0.056352459 |
| P17813 | 1836 | ENG | Endoglin (CD anti | 560 | 1 | 560 | 0.042857143 | 0.033928571 | 0.038392857 |
| Q14416 | 1837 | GRM2 | Metabotropic glut | 591 | 4 | 548 | 0.035532995 | 0.025380711 | 0.030456853 |
| P02724 | 1838 | GYPA | Glycophorin-A (M | 71 | 1 | 71 | 0.267605634 | 0.225352113 | 0.246478873 |
| Q9NZS2 | 1839 | KLRF1 | Killer cell lectin-lik | 171 | 1 | 171 | 0.035087719 | 0.046783626 | 0.040935673 |
| P46531 | 1840 | NOTCH1 | Neurogenic locus | 1716 | 1 | 1716 | 0.032051282 | 0.044871795 | 0.038461538 |
| P30550 | 1841 | GRPR | Gastrin-releasing | 99 | 4 | 37 | 0.02020202 | 0.050505051 | 0.035353535 |
| P08069 | 1842 | IGF1R | Insulin-like growth | 194 | 1 | 194 | 0.072164948 | 0.06185567 | 0.067010309 |
| P27930 | 1843 | IL1R2 | Interleukin-1 rece | 329 | 1 | 329 | 0.048632219 | 0.018237082 | 0.03343465 |
| P23229 | 1844 | ITGA6 | Integrin alpha-6 ( | 1026 | 1 | 1026 | 0.023391813 | 0.022417154 | 0.022904483 |
| O75581 | 1845 | LRP6 | Low-density lipop | 1350 | 1 | 1350 | 0 | 0.013333333 | 0.006666667 |
| Q8N387 | 1846 | MUC15 | Mucin-15 (MUC-1 | 212 | 1 | 212 | 0.183962264 | 0.216981132 | 0.200471698 |
| Q14626 | 1847 | IL11RA | Interleukin-11 rec | 346 | 1 | 346 | 0.049132948 | 0.028901734 | 0.039017341 |
| P05362 | 1848 | ICAM1 | Intercellular adhe | 452 | 1 | 452 | 0.061946903 | 0.017699115 | 0.039823009 |
| Q6UXL0 | 1849 | IL20RB | Interleukin-20 rec | 203 | 1 | 203 | 0.039408867 | 0.014778325 | 0.027093596 |
| P31785 | 1850 | IL2RG | Cytokine receptor | 239 | 1 | 239 | 0.150627615 | 0.033472803 | 0.092050209 |
| P04201 | 1851 | MAS1 | Proto-oncogene M | 81 | 4 | 35 | 0 | 0.111111111 | 0.055555556 |
| Q8IYS2 | 1852 | KIAA2013 | Uncharacterized p | 548 | 1 | 548 | 0.02919708 | 0.012773723 | 0.020985401 |
| Q9NZN1 | 1853 | IL1RAPL1 | Interleukin-1 rece | 338 | 1 | 338 | 0.035502959 | 0.020710059 | 0.028106509 |
| Q04900 | 1854 | CD164 | Sialomucin core p | 138 | 1 | 138 | 0.31884058 | 0.311594203 | 0.315217391 |
| O95944 | 1855 | NCR2 | Natural cytotoxicit | 170 | 1 | 170 | 0.094117647 | 0.117647059 | 0.105882353 |
| Q58DX5 | 1856 | NAALADL2 | Inactive N-acetyla | 652 | 1 | 652 | 0.030674847 | 0.035276074 | 0.03297546 |
| Q9GZQ6 | 1857 | NPFFR1 | Neuropeptide FF r | 105 | 4 | 42 | 0.161904762 | 0.152380952 | 0.157142857 |
| P69849 | 1858 | NOMO3 | Nodal modulator 3 | 1123 | 1 | 1123 | 0.039180766 | 0.011576135 | 0.025378451 |
| Q8NFZ4 | 1859 | NLGN2 | Neuroligin-2 | 662 | 1 | 662 | 0.031722054 | 0.024169184 | 0.027945619 |
| Q9UKJ1 | 1860 | PILRA | Paired immunoglo | 177 | 1 | 177 | 0.129943503 | 0.15819209 | 0.144067797 |
| Q9UKJ0 | 1861 | PILRB | Paired immunoglo | 171 | 1 | 171 | 0.099415205 | 0.122807018 | 0.111111111 |
| Q99463 | 1862 | NPY6R | Putative neuropep | 88 | 4 | 38 | 0.090909091 | 0.113636364 | 0.102272727 |
| Q8NGT7 | 1863 | OR2A12 | Olfactory receptor | 91 | 4 | 36 | 0.032967033 | 0.010989011 | 0.021978022 |
| Q9Y5G8 | 1864 | PCDHGA5 | Protocadherin gar | 662 | 1 | 662 | 0.033232628 | 0.003021148 | 0.018126888 |
| P14867 | 1865 | GABRA1 | Gamma-aminobut | 223 | 1 | 223 | 0.040358744 | 0.013452915 | 0.02690583 |
| Q6DWJ6 | 1866 | GPR139 | Probable G-protei | 72 | 4 | 28 | 0.027777778 | 0.055555556 | 0.041666667 |
| P47775 | 1867 | GPR12 | G-protein coupled | 88 | 4 | 47 | 0 | 0.068181818 | 0.034090909 |
| P46091 | 1868 | GPR1 | G-protein coupled | 108 | 4 | 40 | 0 | 0.009259259 | 0.00462963 |
| P17693 | 1869 | HLA-G | HLA class I histoc | 283 | 1 | 283 | 0.010600707 | 0.003533569 | 0.007067138 |
| Q01638 | 1870 | IL1RL1 | Interleukin-1 rece | 309 | 1 | 309 | 0.045307443 | 0.029126214 | 0.037216828 |
| P32927 | 1871 | CSF2RB | Cytokine receptor | 426 | 1 | 426 | 0.037558685 | 0.011737089 | 0.024647887 |
| Q01344 | 1872 | IL5RA | Interleukin-5 rece | 321 | 1 | 321 | 0.046728972 | 0.037383178 | 0.042056075 |
| A4D2G3 | 1873 | OR2A25 | Olfactory receptor | 92 | 4 | 37 | 0.032608696 | 0.010869565 | 0.02173913 |
| Q8NGZ0 | 1874 | OR2AJ1 | Olfactory receptor | 92 | 4 | 36 | 0.02173913 | 0.010869565 | 0.016304348 |
| Q8NGL0 | 1875 | OR5L2 | Olfactory receptor | 94 | 4 | 35 | 0.031914894 | 0.021276596 | 0.026595745 |
| Q8NGG6 | 1876 | OR8B12 | Olfactory receptor | 94 | 4 | 36 | 0.053191489 | 0.031914894 | 0.042553191 |
| Q8NGX8 | 1877 | OR6Y1 | Olfactory receptor | 100 | 4 | 36 | 0.05 | 0.02 | 0.035 |
| Q9GZK4 | 1878 | OR2H1 | Olfactory receptor | 90 | 4 | 36 | 0.011111111 | 0.011111111 | 0.011111111 |
| Q8NG95 | 1879 | OR7G3 | Olfactory receptor | 95 | 4 | 36 | 0.031578947 | 0.021052632 | 0.026315789 |
| Q8NGL4 | 1880 | OR5D13 | Olfactory receptor | 96 | 4 | 35 | 0.052083333 | 0.010416667 | 0.03125 |
| Q8NGL3 | 1881 | OR5D14 | Olfactory receptor | 96 | 4 | 35 | 0.020833333 | 0.020833333 | 0.020833333 |
| Q15116 | 1882 | PDCD1 | Programmed cell | 146 | 1 | 146 | 0.082191781 | 0.061643836 | 0.071917808 |
| Q8IU57 | 1883 | IFNLR1 | Interferon lambda | 207 | 1 | 207 | 0.057971014 | 0.019323671 | 0.038647343 |
| Q92953 | 1884 | KCNB2 | Potassium voltage | 49 | 4 | 19 | 0.020408163 | 0.020408163 | 0.020408163 |
| Q9UIQ6 | 1885 | LNPEP | Leucyl-cystinyl an | 893 | 1 | 893 | 0.031354983 | 0.022396417 | 0.0268757 |
| Q6NSJ5 | 1886 | LRRC8E | Volume-regulated | 98 | 2 | 72 | 0.030612245 | 0.020408163 | 0.025510204 |
| Q99677 | 1887 | LPAR4 | Lysophosphatidic | 103 | 4 | 42 | 0.058252427 | 0.077669903 | 0.067961165 |
| P32245 | 1888 | MC4R | Melanocortin rece | 70 | 4 | 42 | 0.1 | 0.114285714 | 0.107142857 |
| Q8IX19 | 1889 | MCEMP1 | Mast cell-express | 80 | 1 | 80 | 0.0125 | 0.025 | 0.01875 |
| P08473 | 1890 | MME | Neprilysin (EC 3.4 | 698 | 1 | 698 | 0.015759312 | 0.011461318 | 0.013610315 |
| P15529 | 1891 | CD46 | Membrane cofact | 308 | 1 | 308 | 0.081168831 | 0.068181818 | 0.074675325 |
| P0C617 | 1892 | OR5AL1 | Olfactory receptor | 114 | 4 | 43 | 0.00877193 | 0 | 0.004385965 |
| Q8NHA4 | 1893 | OR2AE1 | Olfactory receptor | 92 | 4 | 36 | 0.02173913 | 0.010869565 | 0.016304348 |
| P0DN82 | 1894 | OR12D1 | Olfactory receptor | 86 | 4 | 34 | 0.069767442 | 0.011627907 | 0.040697674 |
| Q8NGS4 | 1895 | OR13F1 | Olfactory receptor | 95 | 4 | 36 | 0.021052632 | 0.010526316 | 0.015789474 |
| O95221 | 1896 | OR5F1 | Olfactory receptor | 94 | 4 | 35 | 0.042553191 | 0.010638298 | 0.026595745 |
| Q8NGG2 | 1897 | OR5T2 | Olfactory receptor | 133 | 4 | 63 | 0.037593985 | 0.015037594 | 0.026315789 |
| P0C7N5 | 1898 | OR8U9 | Olfactory receptor | 96 | 4 | 39 | 0.041666667 | 0.010416667 | 0.026041667 |
| Q8NGQ5 | 1899 | OR9Q1 | Olfactory receptor | 95 | 4 | 36 | 0.031578947 | 0.010526316 | 0.021052632 |
| Q8NH03 | 1900 | OR2T3 | Olfactory receptor | 97 | 4 | 36 | 0.072164948 | 0.030927835 | 0.051546392 |
| Q8NGH3 | 1901 | OR2D3 | Olfactory receptor | 108 | 4 | 40 | 0.027777778 | 0.009259259 | 0.018518519 |
| Q9Y585 | 1902 | OR1A2 | Olfactory receptor | 91 | 4 | 36 | 0.032967033 | 0.010989011 | 0.021978022 |
| A6NH00 | 1903 | OR2T8 | Olfactory receptor | 93 | 4 | 36 | 0.032258065 | 0.021505376 | 0.02688172 |
| Q6UWI2 | 1904 | PARM1 | Prostate androger | 237 | 1 | 237 | 0.278481013 | 0.362869198 | 0.320675105 |
| Q96RD6 | 1905 | PANX2 | Pannexin-2 | 94 | 2 | 52 | 0.031914894 | 0.010638298 | 0.021276596 |
| Q9Y5E1 | 1906 | PCDHB9 | Protocadherin bet | 663 | 1 | 663 | 0.036199095 | 0.004524887 | 0.020361991 |
| Q9NRJ7 | 1907 | PCDHB16 | Protocadherin bet | 661 | 1 | 661 | 0.040847201 | 0.004538578 | 0.02269289 |
| Q6GTX8 | 1908 | LAIR1 | Leukocyte-associ | 143 | 1 | 143 | 0.076923077 | 0.076923077 | 0.076923077 |
| Q9Y639 | 1909 | NPTN | Neuroplastin (Str | 310 | 1 | 310 | 0.061290323 | 0.029032258 | 0.04516129 |
| O60500 | 1910 | NPHS1 | Nephrin (Renal gl | 1032 | 1 | 1032 | 0.04748062 | 0.016472868 | 0.031976744 |
| P20916 | 1911 | MAG | Myelin-associatec | 496 | 1 | 496 | 0.038306452 | 0.016129032 | 0.027217742 |
| Q16820 | 1912 | MEP1B | Meprin A subunit | 629 | 1 | 629 | 0.066772655 | 0.036565978 | 0.051669316 |
| Q92887 | 1913 | ABCC2 | ATP-binding cass | 248 | 8 | 114 | 0 | 0.012096774 | 0.006048387 |
| Q0D2K0 | 1914 | NIPAL4 | Magnesium transp | 161 | 4 | 116 | 0.049689441 | 0.043478261 | 0.046583851 |
| Q9UM47 | 1915 | NOTCH3 | Neurogenic locus | 1603 | 1 | 1603 | 0.028072364 | 0.051154086 | 0.039613225 |
| O60391 | 1916 | GRIN3B | Glutamate recepto | 701 | 2 | 541 | 0.031383738 | 0.008559201 | 0.019971469 |
| Q9NPH5 | 1917 | NOX4 | NADPH oxidase 4 | 267 | 3 | 214 | 0.033707865 | 0.014981273 | 0.024344569 |
| Q8NH59 | 1918 | OR51Q1 | Olfactory receptor | 98 | 4 | 35 | 0.040816327 | 0.010204082 | 0.025510204 |
| Q8NGI0 | 1919 | OR52N2 | Olfactory receptor | 99 | 4 | 35 | 0.04040404 | 0.01010101 | 0.025252525 |
| Q8NGE5 | 1920 | OR10A7 | Olfactory receptor | 95 | 4 | 36 | 0.042105263 | 0.010526316 | 0.026315789 |
| O14917 | 1921 | PCDH17 | Protocadherin-17 | 689 | 1 | 689 | 0.015965167 | 0.013062409 | 0.014513788 |
| Q9Y5H7 | 1922 | PCDHA5 | Protocadherin alp | 667 | 1 | 667 | 0.020989505 | 0.007496252 | 0.014242879 |
| Q9UN74 | 1923 | PCDHA4 | Protocadherin alp | 667 | 1 | 667 | 0.026986507 | 0.007496252 | 0.017241379 |
| Q9Y5I4 | 1924 | PCDHAC2 | Protocadherin alp | 665 | 1 | 665 | 0.02556391 | 0.007518797 | 0.016541353 |
| Q9Y5H4 | 1925 | PCDHGA1 | Protocadherin gar | 663 | 1 | 663 | 0.031674208 | 0.007541478 | 0.019607843 |
| Q9Y5I1 | 1926 | PCDHA11 | Protocadherin alp | 666 | 1 | 666 | 0.028528529 | 0.007507508 | 0.018018018 |
| Q9Y5G1 | 1927 | PCDHGB3 | Protocadherin gar | 660 | 1 | 660 | 0.025757576 | 0.009090909 | 0.017424242 |
| P43088 | 1928 | PTGFR | Prostaglandin F2- | 87 | 4 | 30 | 0.068965517 | 0.045977011 | 0.057471264 |
| A8MVS5 | 1929 | HIDE1 | Protein HIDE1 | 97 | 1 | 97 | 0.06185567 | 0.041237113 | 0.051546392 |
| P46695 | 1930 | IER3 | Radiation-inducib | 56 | 1 | 56 | 0.053571429 | 0.071428571 | 0.0625 |
| P15260 | 1931 | IFNGR1 | Interferon gamma | 227 | 1 | 227 | 0.052863436 | 0.022026432 | 0.037444934 |
| Q5PT55 | 1932 | SLC10A5 | Sodium/bile acid | 160 | 5 | 122 | 0.0375 | 0.01875 | 0.028125 |
| P49281 | 1933 | SLC11A2 | Natural resistance | 82 | 6 | 37 | 0.036585366 | 0.048780488 | 0.042682927 |
| P34903 | 1934 | GABRA3 | Gamma-aminobut | 247 | 1 | 247 | 0.04048583 | 0.024291498 | 0.032388664 |

| | | | | | | | | | |
|---|---|---|---|---|---|---|---|---|---|
| Q9NU53 | 1935 | GINM1 | Glycoprotein integ | 244 | 1 | 244 | 0.036885246 | 0.057377049 | 0.047131148 |
| A8MPY1 | 1936 | GABRR3 | Gamma-aminobut | 252 | 2 | 242 | 0.01984127 | 0.03968254 | 0.029761905 |
| Q8NFN8 | 1937 | GPR156 | Probable G-protei | 101 | 4 | 46 | 0.089108911 | 0.108910891 | 0.099009901 |
| Q86Z14 | 1938 | KLB | Beta-klotho (BKL) | 995 | 1 | 995 | 0.046231156 | 0.023115578 | 0.034673367 |
| Q4KMZ8 | 1939 | NKAIN1 | Sodium/potassiur | 75 | 2 | 63 | 0.04 | 0.013333333 | 0.026666667 |
| O14786 | 1940 | NRP1 | Neuropilin-1 (Vas | 834 | 1 | 834 | 0.027577938 | 0.03117506 | 0.029376499 |
| P58401 | 1941 | NRXN2 | Neurexin-2-beta ( | 539 | 1 | 539 | 0.087198516 | 0.113172542 | 0.100185529 |
| Q9UN75 | 1942 | PCDHA12 | Protocadherin alp | 667 | 1 | 667 | 0.014992504 | 0.007496252 | 0.011244378 |
| Q9Y5E8 | 1943 | PCDHB15 | Protocadherin bet | 663 | 1 | 663 | 0.031674208 | 0.003016591 | 0.0173454 |
| P59901 | 1944 | LILRA4 | Leukocyte immun | 422 | 1 | 422 | 0.052132701 | 0.030805687 | 0.041469194 |
| P43657 | 1945 | LPAR6 | Lysophosphatidic | 74 | 4 | 26 | 0.108108108 | 0.040540541 | 0.074324324 |
| Q8N0W4 | 1946 | NLGN4X | Neuroligin-4, X-lir | 634 | 1 | 634 | 0.041009464 | 0.039432177 | 0.04022082 |
| P28336 | 1947 | NMBR | Neuromedin-B rec | 101 | 4 | 40 | 0.128712871 | 0.118811881 | 0.123762376 |
| Q9NZM6 | 1948 | PKD2L2 | Polycystic kidney | 288 | 3 | 223 | 0.041666667 | 0.027777778 | 0.034722222 |
| Q9P2S2 | 1949 | NRXN2 | Neurexin-2 (Neur | 1607 | 1 | 1607 | 0.04044804 | 0.048537648 | 0.044492844 |
| Q8NGF0 | 1950 | OR52B6 | Olfactory receptor | 117 | 4 | 44 | 0.025641026 | 0 | 0.012820513 |
| Q9Y5E4 | 1951 | PCDHB5 | Protocadherin bet | 658 | 1 | 658 | 0.044072948 | 0.006079027 | 0.025075988 |
| O95206 | 1952 | PCDH8 | Protocadherin-8 ( | 719 | 1 | 719 | 0.083449235 | 0.015299026 | 0.049374131 |
| Q9UBS5 | 1953 | GABBR1 | Gamma-aminobut | 633 | 4 | 576 | 0.034755134 | 0.028436019 | 0.031595577 |
| Q9UN88 | 1954 | GABRQ | Gamma-aminobut | 246 | 1 | 246 | 0.032520325 | 0.004065041 | 0.018292683 |
| Q86XS8 | 1955 | RNF130 | E3 ubiquitin-prote | 166 | 1 | 166 | 0.054216867 | 0.030120482 | 0.042168675 |
| Q99679 | 1956 | GPR21 | Probable G-protei | 69 | 4 | 31 | 0.057971014 | 0.086956522 | 0.072463768 |
| O15218 | 1957 | GPR182 | G-protein couplec | 110 | 4 | 56 | 0 | 0.072727273 | 0.036363636 |
| Q9Y691 | 1958 | KCNMB2 | Calcium-activatec | 126 | 1 | 126 | 0.031746032 | 0.023809524 | 0.027777778 |
| O75023 | 1959 | LILRB5 | Leukocyte immun | 434 | 1 | 434 | 0.039170507 | 0.029953917 | 0.034562212 |
| Q7Z3D4 | 1960 | LYSMD3 | LysM and putative | 216 | 1 | 216 | 0.027777778 | 0.097222222 | 0.0625 |
| P41968 | 1961 | MC3R | Melanocortin rece | 65 | 4 | 36 | 0.092307692 | 0.076923077 | 0.084615385 |
| P08100 | 1962 | RHO | Rhodopsin (Opsin | 85 | 4 | 35 | 0.094117647 | 0.070588235 | 0.082352941 |
| Q8NH55 | 1963 | OR52E5 | Olfactory receptor | 93 | 4 | 29 | 0.043010753 | 0.010752688 | 0.02688172 |
| A6NHA9 | 1964 | OR4C46 | Olfactory receptor | 104 | 4 | 39 | 0.048076923 | 0.009615385 | 0.028846154 |
| Q13003 | 1965 | GRIK3 | Glutamate recept | 693 | 2 | 531 | 0.03030303 | 0.024531025 | 0.027417027 |
| P51787 | 1966 | KCNQ1 | Potassium voltage | 33 | 4 | 16 | 0.03030303 | 0.03030303 | 0.03030303 |
| Q9H756 | 1967 | LRRC19 | Leucine-rich repe | 245 | 1 | 245 | 0.053061224 | 0.07755102 | 0.065306122 |
| P29376 | 1968 | LTK | Leukocyte tyrosin | 407 | 1 | 407 | 0.054054054 | 0.054054054 | 0.054054054 |
| P42702 | 1969 | LIFR | Leukemia inhibito | 788 | 1 | 788 | 0.049492386 | 0.035532995 | 0.04251269 |
| A0A1B0GTW7 | 1970 | LMLN2 | Leishmanolysin-li | 714 | 1 | 714 | 0 | 0 | 0 |
| Q5XG99 | 1971 | LYSMD4 | LysM and putative | 216 | 1 | 216 | 0.027777778 | 0.055555556 | 0.041666667 |
| Q9Y5Y7 | 1972 | LYVE1 | Lymphatic vessel | 218 | 1 | 218 | 0.123853211 | 0.128440367 | 0.126146789 |
| P43121 | 1973 | MCAM | Cell surface glyco | 535 | 1 | 535 | 0.057943925 | 0.039252336 | 0.048598131 |
| Q9UI40 | 1974 | SLC24A2 | Sodium/potassiur | 110 | 5 | 72 | 0.045454545 | 0.090909091 | 0.068181818 |
| O15394 | 1975 | NCAM2 | Neural cell adhesi | 677 | 1 | 677 | 0.028064993 | 0.013293944 | 0.020679468 |
| Q99466 | 1976 | NOTCH4 | Neurogenic locus | 1423 | 1 | 1423 | 0.04075896 | 0.057624736 | 0.049191848 |
| Q07444 | 1977 | KLRC3 | NKG2-E type II int | 146 | 1 | 146 | 0.034246575 | 0.006849315 | 0.020547945 |
| Q9Y4C0 | 1978 | NRXN3 | Neurexin-3 (Neur | 1540 | 1 | 1540 | 0.093506494 | 0.063636364 | 0.078571429 |
| P35372 | 1979 | OPRM1 | Mu-type opioid re | 106 | 4 | 67 | 0 | 0.160377358 | 0.080188679 |
| Q8NGZ3 | 1980 | OR13G1 | Olfactory receptor | 92 | 4 | 36 | 0.032608696 | 0.010869565 | 0.02173913 |
| Q8NGR1 | 1981 | OR13A1 | Olfactory receptor | 113 | 4 | 42 | 0.017699115 | 0.017699115 | 0.017699115 |
| P58180 | 1982 | OR4D2 | Olfactory receptor | 90 | 4 | 36 | 0.033333333 | 0.011111111 | 0.022222222 |
| Q8NG99 | 1983 | OR7G2 | Olfactory receptor | 95 | 4 | 36 | 0.031578947 | 0.021052632 | 0.026315789 |
| Q8NHB8 | 1984 | OR5K2 | Olfactory receptor | 94 | 4 | 35 | 0.031914894 | 0.010638298 | 0.021276596 |
| Q8NGG4 | 1985 | OR8H1 | Olfactory receptor | 94 | 4 | 36 | 0.042553191 | 0.010638298 | 0.026595745 |
| Q8NGD3 | 1986 | OR4K5 | Olfactory receptor | 90 | 4 | 36 | 0.044444444 | 0.011111111 | 0.027777778 |
| Q15622 | 1987 | OR7A5 | Olfactory receptor | 95 | 4 | 36 | 0.042105263 | 0.010526316 | 0.026315789 |
| A6NET4 | 1988 | OR5K3 | Olfactory receptor | 86 | 4 | 31 | 0.023255814 | 0.011627907 | 0.01744186 |
| Q8NGU2 | 1989 | OR9A4 | Olfactory receptor | 95 | 4 | 36 | 0.031578947 | 0.021052632 | 0.026315789 |
| P56373 | 1990 | P2RX3 | P2X purinoceptor | 278 | 1 | 278 | 0.035971223 | 0.021582734 | 0.028776978 |
| O95256 | 1991 | IL18RAP | Interleukin-18 rec | 336 | 1 | 336 | 0.023809524 | 0.014880952 | 0.019345238 |
| P32942 | 1992 | ICAM3 | Intercellular adhe | 455 | 1 | 455 | 0.072527473 | 0.037362637 | 0.054945055 |
| P13612 | 1993 | ITGA4 | Integrin alpha-4 ( | 942 | 1 | 942 | 0.025477707 | 0.020169851 | 0.022823779 |
| Q6UX15 | 1994 | LAYN | Layilin | 213 | 1 | 213 | 0.018779343 | 0.023474178 | 0.021126761 |
| P55344 | 1995 | LIM2 | Lens fiber membra | 61 | 2 | 41 | 0.032786885 | 0.016393443 | 0.024590164 |
| Q8TD46 | 1996 | CD200R1 | Cell surface glyco | 214 | 1 | 214 | 0.070093458 | 0.037383178 | 0.053738318 |
| Q9BPV8 | 1997 | P2RY13 | P2Y purinoceptor | 120 | 4 | 48 | 0.05 | 0.033333333 | 0.041666667 |
| Q9NPC2 | 1998 | KCNK9 | Potassium channe | 87 | 4 | 58 | 0.045977011 | 0.011494253 | 0.028735632 |
| Q9NZR2 | 1999 | LRP1B | Low-density lipop | 4419 | 1 | 4419 | 0 | 0.038470242 | 0.019235121 |
| Q86YC3 | 2000 | NRROS | Transforming grov | 631 | 1 | 631 | 0.031695721 | 0.034865293 | 0.033280507 |
| Q96NI6 | 2001 | LRFN5 | Leucine-rich repe | 511 | 1 | 511 | 0.109589041 | 0.046966732 | 0.078277886 |
| O95297 | 2002 | MPZL1 | Myelin protein zer | 126 | 1 | 126 | 0.19047619 | 0.015873016 | 0.103174603 |
| Q9Y5G4 | 2003 | PCDHGA9 | Protocadherin gar | 663 | 1 | 663 | 0.037707391 | 0.006033183 | 0.021870287 |
| Q9Y5I2 | 2004 | PCDHA10 | Protocadherin alp | 666 | 1 | 666 | 0.025525526 | 0.009009009 | 0.017267267 |
| Q9NQS3 | 2005 | NECTIN3 | Nectin-3 (CDw11 | 346 | 1 | 346 | 0.089595376 | 0.026011561 | 0.057803468 |
| Q9H2C8 | 2006 | OR51V1 | Olfactory receptor | 105 | 4 | 35 | 0.104761905 | 0 | 0.052380952 |
| Q8NGY1 | 2007 | OR10Z1 | Olfactory receptor | 95 | 4 | 36 | 0.031578947 | 0.010526316 | 0.021052632 |
| Q96RA2 | 2008 | OR7D2 | Olfactory receptor | 95 | 4 | 36 | 0.042105263 | 0 | 0.021052632 |
| Q8NGC5 | 2009 | OR6J1 | Olfactory receptor | 94 | 4 | 36 | 0.042553191 | 0.010638298 | 0.026595745 |
| Q9P1Q5 | 2010 | OR1A1 | Olfactory receptor | 91 | 4 | 36 | 0.021978022 | 0.021978022 | 0.021978022 |
| Q8NH06 | 2011 | OR1P1 | Olfactory receptor | 113 | 4 | 50 | 0.03539823 | 0 | 0.017699115 |
| O95838 | 2012 | GLP2R | Glucagon-like pep | 225 | 4 | 172 | 0.044444444 | 0.053333333 | 0.048888889 |
| Q9UQ05 | 2013 | KCNH4 | Potassium voltage | 94 | 4 | 44 | 0.010638298 | 0.031914894 | 0.021276596 |
| Q14392 | 2014 | LRRC32 | Transforming grov | 607 | 1 | 607 | 0.014827018 | 0.02306425 | 0.018945634 |
| P08581 | 2015 | MET | Hepatocyte growt | 907 | 1 | 907 | 0.030871003 | 0.020948181 | 0.025909592 |
| P04629 | 2016 | NTRK1 | High affinity nerve | 390 | 1 | 390 | 0.046153846 | 0.051282051 | 0.048717949 |
| P25189 | 2017 | MPZ | Myelin protein P0 | 123 | 1 | 123 | 0.008130081 | 0.008130081 | 0.008130081 |
| Q9UPR5 | 2018 | SLC8A2 | Sodium/calcium e | 112 | 6 | 47 | 0.071428571 | 0.044642857 | 0.058035714 |
| O15146 | 2019 | MUSK | Muscle, skeletal r | 471 | 1 | 471 | 0.057324841 | 0.036093418 | 0.04670913 |
| Q8N130 | 2020 | SLC34A3 | Sodium-depender | 184 | 4 | 112 | 0.086956522 | 0.032608696 | 0.059782609 |
| Q13563 | 2021 | PKD2 | Polycystin-2 (PC2 | 269 | 4 | 226 | 0.05204461 | 0.018587361 | 0.035315985 |
| Q9NTG1 | 2022 | PKDREJ | Polycystic kidney | 1481 | 6 | 1164 | 0.034436192 | 0.020931803 | 0.027683997 |
| Q15761 | 2023 | NPY5R | Neuropeptide Y re | 105 | 4 | 41 | 0.057142857 | 0.066666667 | 0.061904762 |
| P08F94 | 2024 | PKHD1 | Fibrocystin (Polyc | 3834 | 1 | 3834 | 0.039645279 | 0.03103808 | 0.03534168 |
| P01133 | 2025 | EGF | Pro-epidermal grc | 1009 | 1 | 1009 | 0 | 0.065411298 | 0.032705649 |
| Q14627 | 2026 | IL13RA2 | Interleukin-13 rec | 316 | 1 | 316 | 0.028481013 | 0.025316456 | 0.026898734 |
| Q13349 | 2027 | ITGAD | Integrin alpha-D ( | 1081 | 1 | 1081 | 0.025901943 | 0.03052729 | 0.028214616 |
| Q13797 | 2028 | ITGA9 | Integrin alpha-9 ( | 951 | 1 | 951 | 0.027339642 | 0.014721346 | 0.021030494 |
| P17181 | 2029 | IFNAR1 | Interferon alpha/t | 408 | 1 | 408 | 0.039215686 | 0.024509804 | 0.031862745 |
| P18564 | 2030 | ITGB6 | Integrin beta-6 | 687 | 1 | 687 | 0.026200873 | 0.045123726 | 0.0356623 |
| Q96MU8 | 2031 | KREMEN1 | Kremen protein 1 | 371 | 1 | 371 | 0.061994609 | 0.04851752 | 0.055256065 |
| Q8TDS7 | 2032 | MRGPRD | Mas-related G-pr | 100 | 4 | 32 | 0 | 0.07 | 0.035 |
| Q8N2Q7 | 2033 | NLGN1 | Neuroligin-1 | 671 | 1 | 671 | 0.035767511 | 0.04023845 | 0.038002981 |
| O15399 | 2034 | GRIN2D | Glutamate recept | 726 | 2 | 556 | 0.023415978 | 0.012396694 | 0.017906336 |
| Q06495 | 2035 | SLC34A1 | Sodium-depender | 213 | 4 | 161 | 0.061032864 | 0.014084507 | 0.037558685 |
| Q15223 | 2036 | NECTIN1 | Nectin-1 (Herpes | 324 | 1 | 324 | 0.061728395 | 0.030864198 | 0.046296296 |
| P14778 | 2037 | IL1R1 | Interleukin-1 rece | 318 | 1 | 318 | 0.031446541 | 0.01572327 | 0.023584906 |
| Q6UXK2 | 2038 | ISLR2 | Immunoglobulin s | 570 | 1 | 570 | 0.040350877 | 0.036842105 | 0.038596491 |
| P06756 | 2039 | ITGAV | Integrin alpha-V ( | 961 | 1 | 961 | 0.027055151 | 0.018730489 | 0.02289282 |
| P26012 | 2040 | ITGB8 | Integrin beta-8 | 641 | 1 | 641 | 0.017160686 | 0.037441498 | 0.027301092 |
| P43631 | 2041 | KIR2DS2 | Killer cell immuno | 223 | 1 | 223 | 0.067264574 | 0.035874439 | 0.051569507 |
| Q96KG7 | 2042 | MEGF10 | Multiple epiderma | 831 | 1 | 831 | 0.08423586 | 0.087845969 | 0.086040915 |
| Q16288 | 2043 | NTRK3 | NT-3 growth facto | 397 | 1 | 397 | 0 | 0.037783375 | 0.018891688 |
| Q96RD3 | 2044 | OR52E6 | Olfactory receptor | 98 | 4 | 35 | 0.020408163 | 0.010204082 | 0.015306122 |
| Q96R54 | 2045 | OR14A2 | Olfactory receptor | 82 | 4 | 35 | 0.073170732 | 0.036585366 | 0.054878049 |
| Q8NH81 | 2046 | OR10G6 | Olfactory receptor | 114 | 4 | 45 | 0.01754386 | 0.00877193 | 0.013157895 |
| Q8NGV5 | 2047 | OR13D1 | Olfactory receptor | 127 | 4 | 56 | 0.031496063 | 0.031496063 | 0.031496063 |
| Q8NH42 | 2048 | OR4K13 | Olfactory receptor | 90 | 4 | 36 | 0.011111111 | 0.011111111 | 0.011111111 |

| | | | | | | | | | |
|---|---|---|---|---|---|---|---|---|---|
| Q8NH41 | 2049 | OR4K15 | Olfactory receptor | 114 | 4 | 48 | 0.052631579 | 0.01754386 | 0.035087719 |
| P0DMU2 | 2050 | OR8G3P | Putative olfactory | 95 | 4 | 36 | 0.031578947 | 0.010526316 | 0.021052632 |
| Q8NGQ6 | 2051 | OR9I1 | Olfactory receptor | 95 | 4 | 36 | 0.052631579 | 0.010526316 | 0.031578947 |
| Q6UXG2 | 2052 | ELAPOR1 | Endosome/lysoso | 868 | 1 | 868 | 0.129032258 | 0.125576037 | 0.127304147 |
| P40197 | 2053 | GP5 | Platelet glycoprot | 506 | 1 | 506 | 0.033596838 | 0.035573123 | 0.03458498 |
| Q99527 | 2054 | GPER1 | G-protein coupled | 122 | 4 | 61 | 0 | 0.106557377 | 0.053278689 |
| P43220 | 2055 | GLP1R | Glucagon-like pep | 166 | 4 | 115 | 0.042168675 | 0.024096386 | 0.03313253 |
| Q8WUT4 | 2056 | LRRN4 | Leucine-rich repe | 660 | 1 | 660 | 0.071212121 | 0.096969697 | 0.084090909 |
| P51511 | 2057 | MMP15 | Matrix metallopro | 493 | 1 | 493 | 0.018255578 | 0.010141988 | 0.014198783 |
| Q9GZQ4 | 2058 | NMUR2 | Neuromedin-U rec | 108 | 4 | 48 | 0.064814815 | 0.037037037 | 0.050925926 |
| P21452 | 2059 | TACR2 | Substance-K rece | 89 | 4 | 31 | 0.078651685 | 0.101123596 | 0.08988764 |
| Q9UNW8 | 2060 | GPR132 | Probable G-protei | 93 | 4 | 44 | 0.075268817 | 0.075268817 | 0.075268817 |
| Q86SP6 | 2061 | GPR149 | Probable G-protei | 74 | 4 | 34 | 0.067567568 | 0.081081081 | 0.074324324 |
| P11169 | 2062 | SLC2A3 | Solute carrier fam | 76 | 6 | 31 | 0.026315789 | 0.013157895 | 0.019736842 |
| P04921 | 2063 | GYPC | Glycophorin-C (Gl | 56 | 1 | 56 | 0.321428571 | 0.267857143 | 0.294642857 |
| Q8WWG9 | 2064 | KCNE4 | Potassium voltage | 85 | 1 | 85 | 0.105882353 | 0.129411765 | 0.117647059 |
| Q9HBX8 | 2065 | LGR6 | Leucine-rich repe | 597 | 4 | 542 | 0.011725293 | 0.015075377 | 0.013400335 |
| A6NMS7 | 2066 | LRRC37A | Leucine-rich repe | 1546 | 1 | 1546 | 0.067917206 | 0.130659767 | 0.099288486 |
| Q86UE6 | 2067 | LRRTM1 | Leucine-rich repe | 392 | 1 | 392 | 0.035714286 | 0.045918367 | 0.040816327 |
| O76036 | 2068 | NCR1 | Natural cytotoxicit | 236 | 1 | 236 | 0.050847458 | 0.012711864 | 0.031779661 |
| O60721 | 2069 | SLC24A1 | Sodium/potassiur | 484 | 5 | 451 | 0.136363636 | 0.233471074 | 0.184917355 |
| Q8NGK4 | 2070 | OR52K1 | Olfactory receptor | 98 | 4 | 35 | 0.040816327 | 0.020408163 | 0.030612245 |
| Q96RD2 | 2071 | OR52B2 | Olfactory receptor | 98 | 4 | 35 | 0.040816327 | 0.010204082 | 0.025510204 |
| Q8NG84 | 2072 | OR2AK2 | Olfactory receptor | 107 | 4 | 39 | 0.028037383 | 0.018691589 | 0.023364486 |
| Q8NH95 | 2073 | OR13C6P | Putative olfactory | 43 | 2 | 26 | 0.11627907 | 0 | 0.058139535 |
| Q8NGA5 | 2074 | OR10H4 | Olfactory receptor | 96 | 4 | 36 | 0.041666667 | 0.020833333 | 0.03125 |
| Q8NG94 | 2075 | OR11H1 | Olfactory receptor | 103 | 4 | 43 | 0.029126214 | 0.019417476 | 0.024271845 |
| Q8NGM1 | 2076 | OR4C15 | Olfactory receptor | 88 | 4 | 36 | 0.022727273 | 0.011363636 | 0.017045455 |
| P0DN78 | 2077 | OPN1MW3 | Medium-wave-se | 96 | 4 | 51 | 0.21875 | 0.104166667 | 0.161458333 |
| Q8NGL9 | 2078 | OR4C16 | Olfactory receptor | 88 | 4 | 36 | 0.034090909 | 0.011363636 | 0.022727273 |
| P0C7N8 | 2079 | OR9G9 | Olfactory receptor | 86 | 4 | 40 | 0.069767442 | 0.011627907 | 0.040697674 |
| Q8NGF8 | 2080 | OR4B1 | Olfactory receptor | 88 | 4 | 36 | 0.079545455 | 0.011363636 | 0.045454545 |
| Q8NGA1 | 2081 | OR1M1 | Olfactory receptor | 93 | 4 | 37 | 0.043010753 | 0.010752688 | 0.02688172 |
| Q8NG81 | 2082 | OR2M7 | Olfactory receptor | 92 | 4 | 36 | 0.054347826 | 0.02173913 | 0.038043478 |
| Q8NGS0 | 2083 | OR1N1 | Olfactory receptor | 91 | 4 | 37 | 0.032967033 | 0.010989011 | 0.021978022 |
| Q8N628 | 2084 | OR2C3 | Olfactory receptor | 93 | 4 | 36 | 0.032258065 | 0.010752688 | 0.021505376 |
| P30953 | 2085 | OR1E1 | Olfactory receptor | 92 | 4 | 36 | 0.02173913 | 0.010869565 | 0.016304348 |
| Q13607 | 2086 | OR2F1 | Olfactory receptor | 92 | 4 | 39 | 0.032608696 | 0.010869565 | 0.02173913 |
| Q8NG97 | 2087 | OR2Z1 | Olfactory receptor | 92 | 4 | 36 | 0.02173913 | 0 | 0.010869565 |
| Q13002 | 2088 | GRIK2 | Glutamate recepto | 691 | 2 | 529 | 0.054992764 | 0.017366136 | 0.03617945 |
| Q9BZV3 | 2089 | IMPG2 | Interphotorecepto | 1076 | 1 | 1076 | 0.05204461 | 0.151486989 | 0.101765799 |
| P23276 | 2090 | KEL | Kell blood group g | 664 | 1 | 664 | 0.02560241 | 0.022590361 | 0.024096386 |
| Q12908 | 2091 | SLC10A2 | Ileal sodium/bile a | 103 | 4 | 38 | 0.029126214 | 0.009708738 | 0.019417476 |
| Q8NH54 | 2092 | OR56A3 | Olfactory receptor | 100 | 4 | 35 | 0.04 | 0.02 | 0.03 |
| Q8NGX1 | 2093 | OR2T34 | Olfactory receptor | 97 | 4 | 36 | 0.072164948 | 0.030927835 | 0.051546392 |
| Q9UKH3 | 2094 | ERVK-9 | Endogenous retro | 541 | 1 | 541 | 0.035120148 | 0.01663586 | 0.025878004 |
| Q17R55 | 2095 | FAM187B | Protein FAM187B | 317 | 1 | 317 | 0.018927445 | 0.012618297 | 0.015772871 |
| Q15375 | 2096 | EPHA7 | Ephrin type-A rece | 527 | 1 | 527 | 0.028462998 | 0.024667932 | 0.026565465 |
| Q92637 | 2097 | FCGR1B | High affinity immu | 182 | 1 | 182 | 0.076923077 | 0.027472527 | 0.052197802 |
| Q8NGA4 | 2098 | GPR32P1 | Putative G-proteir | 94 | 3 | 45 | 0.042553191 | 0.085106383 | 0.063829787 |
| Q9NYQ8 | 2099 | FAT2 | Protocadherin Fat | 4029 | 1 | 4029 | 0.038719285 | 0.010176222 | 0.024447754 |
| O14626 | 2100 | GPR171 | Probable G-protei | 88 | 4 | 27 | 0.056818182 | 0.022727273 | 0.039772727 |
| O75311 | 2101 | GLRA3 | Glycine receptor s | 243 | 3 | 221 | 0.016460905 | 0.024691358 | 0.020576132 |
| Q14943 | 2102 | KIR3DS1 | Killer cell immuno | 318 | 1 | 318 | 0.072327044 | 0.034591195 | 0.053459119 |
| P18627 | 2103 | LAG3 | Lymphocyte activa | 427 | 1 | 427 | 0.088992974 | 0.023419204 | 0.056206089 |
| P01130 | 2104 | LDLR | Low-density lipop | 766 | 1 | 766 | 0 | 0.05613577 | 0.028067885 |
| Q9ULI3 | 2105 | HEG1 | Protein HEG homo | 1218 | 1 | 1218 | 0.148604269 | 0.284893268 | 0.216748768 |
| Q5VV43 | 2106 | KIAA0319 | Dyslexia-associat | 934 | 1 | 934 | 0.059957173 | 0.085653105 | 0.072805139 |
| Q9Y219 | 2107 | JAG2 | Protein jagged-2 | 1053 | 1 | 1053 | 0.025641026 | 0.062678063 | 0.044159544 |
| P43632 | 2108 | KIR2DS4 | Killer cell immuno | 223 | 1 | 223 | 0.076233184 | 0.044843049 | 0.060538117 |
| Q09470 | 2109 | KCNA1 | Potassium voltage | 64 | 4 | 33 | 0.0625 | 0.015625 | 0.0390625 |
| Q8NCW0 | 2110 | KREMEN2 | Kremen protein 2 | 338 | 1 | 338 | 0.044378698 | 0.038461538 | 0.041420118 |
| Q9HBW1 | 2111 | LRRC4 | Leucine-rich repe | 488 | 1 | 488 | 0.06557377 | 0.06147541 | 0.06352459 |
| P55082 | 2112 | MFAP3 | Microfibril-associa | 128 | 1 | 128 | 0.046875 | 0.0234375 | 0.03515625 |
| Q9NP99 | 2113 | TREM1 | Triggering recepto | 184 | 1 | 184 | 0.097826087 | 0.048913043 | 0.073369565 |
| Q9UNG2 | 2114 | TNFSF18 | Tumor necrosis fa | 127 | 1 | 127 | 0.015748031 | 0.015748031 | 0.015748031 |
| Q9H1D0 | 2115 | TRPV6 | Transient recepto | 63 | 4 | 36 | 0.031746032 | 0.015873016 | 0.023809524 |
| Q96SJ8 | 2116 | TSPAN18 | Tetraspanin-18 (T | 132 | 2 | 118 | 0.037878788 | 0.03030303 | 0.034090909 |
| Q8IU80 | 2117 | TMPRSS6 | Transmembrane p | 734 | 1 | 734 | 0.019073569 | 0.014986376 | 0.017029973 |
| Q8NE00 | 2118 | TMEM104 | Transmembrane p | 93 | 6 | 36 | 0.053763441 | 0.010752688 | 0.032258065 |
| P19075 | 2119 | TSPAN8 | Tetraspanin-8 (Ts | 118 | 2 | 95 | 0.016949153 | 0.025423729 | 0.021186441 |
| Q96FV3 | 2120 | TSPAN17 | Tetraspanin-17 (T | 140 | 2 | 118 | 0.021428571 | 0.028571429 | 0.025 |
| Q8IZJ1 | 2121 | UNC5B | Netrin receptor UI | 350 | 1 | 350 | 0.025714286 | 0.04 | 0.032857143 |
| Q96RI9 | 2122 | TAAR9 | Trace amine-asso | 93 | 4 | 36 | 0.032258065 | 0.032258065 | 0.032258065 |
| Q9NYW0 | 2123 | TAS2R10 | Taste receptor typ | 80 | 4 | 36 | 0.025 | 0.025 | 0.025 |
| P59537 | 2124 | TAS2R43 | Taste receptor typ | 56 | 3 | 30 | 0.035714286 | 0.035714286 | 0.035714286 |
| Q5T4T1 | 2125 | TMEM170B | Transmembrane p | 50 | 2 | 36 | 0.04 | 0.04 | 0.04 |
| Q9NYV8 | 2126 | TAS2R14 | Taste receptor typ | 56 | 4 | 33 | 0.125 | 0.053571429 | 0.089285714 |
| O60637 | 2127 | TSPAN3 | Tetraspanin-3 (Ts | 122 | 2 | 105 | 0.032786885 | 0.057377049 | 0.045081967 |
| P59540 | 2128 | TAS2R46 | Taste receptor typ | 56 | 3 | 30 | 0.035714286 | 0.035714286 | 0.035714286 |
| A6NC51 | 2129 | TMEM150B | Modulator of mac | 40 | 3 | 22 | 0.025 | 0.025 | 0.025 |
| Q9NT68 | 2130 | TENM2 | Teneurin-2 (Ten- | 2373 | 1 | 2373 | 0.028234303 | 0.016434893 | 0.022334598 |
| Q8NHX9 | 2131 | TPCN2 | Two pore calcium | 132 | 8 | 33 | 0.045454545 | 0.037878788 | 0.041666667 |
| O00206 | 2132 | TLR4 | Toll-like receptor | 607 | 1 | 607 | 0 | 0.02306425 | 0.011532125 |
| P62079 | 2133 | TSPAN5 | Tetraspanin-5 (Ts | 140 | 2 | 118 | 0.028571429 | 0.042857143 | 0.035714286 |
| E5RIL1 | 2134 | UPK3BL2 | Uroplakin-3b-like | 170 | 1 | 170 | 0.058823529 | 0.058823529 | 0.058823529 |
| O15393 | 2135 | TMPRSS2 | Transmembrane p | 386 | 1 | 386 | 0.03626943 | 0.03626943 | 0.03626943 |
| Q24JP5 | 2136 | TMEM132A | Transmembrane p | 816 | 1 | 816 | 0.073529412 | 0.022058824 | 0.047794118 |
| B6A8C7 | 2137 | TARM1 | T-cell-interacting, | 219 | 1 | 219 | 0 | 0.02739726 | 0.01369863 |
| Q9P0T7 | 2138 | TMEM9 | Proton-transportir | 68 | 1 | 68 | 0.029411765 | 0.044117647 | 0.036764706 |
| Q92956 | 2139 | TNFRSF14 | Tumor necrosis fa | 163 | 1 | 163 | 0.055214724 | 0.085889571 | 0.070552147 |
| Q86WS5 | 2140 | TMPRSS12 | Transmembrane p | 303 | 1 | 303 | 0.01980198 | 0.01980198 | 0.01980198 |
| A6NFA1 | 2141 | TRABD2B | Metalloprotease T | 474 | 1 | 474 | 0.052742616 | 0.069620253 | 0.061181435 |
| Q69384 | 2142 | ERVK-6 | Endogenous retro | 542 | 1 | 542 | 0.040590406 | 0.020295203 | 0.030442804 |
| Q8NAU1 | 2143 | FNDC5 | Fibronectin type II | 120 | 1 | 120 | 0.025 | 0.025 | 0.025 |
| P08637 | 2144 | FCGR3A | Low affinity immu | 191 | 1 | 191 | 0.047120419 | 0.02617801 | 0.036649215 |
| Q8TDW7 | 2145 | FAT3 | Protocadherin Fat | 4121 | 1 | 4121 | 0.031060422 | 0.008493084 | 0.019776753 |
| Q9UP38 | 2146 | FZD1 | Frizzled-1 (Fz-1) | 340 | 4 | 249 | 0.029411765 | 0.041176471 | 0.035294118 |
| Q6PRD1 | 2147 | GPR179 | Probable G-protei | 402 | 4 | 355 | 0.019900498 | 0.017412935 | 0.018656716 |
| P14672 | 2148 | SLC2A4 | Solute carrier fam | 81 | 6 | 35 | 0.086419753 | 0.024691358 | 0.055555556 |
| P42701 | 2149 | IL12RB1 | Interleukin-12 rec | 521 | 1 | 521 | 0.044145873 | 0.015355086 | 0.02975048 |
| Q9HBE5 | 2150 | IL21R | Interleukin-21 rec | 212 | 1 | 212 | 0.04245283 | 0.004716981 | 0.023584906 |
| Q8NI17 | 2151 | IL31RA | Interleukin-31 rec | 499 | 1 | 499 | 0.078156313 | 0.004008016 | 0.041082164 |
| Q6UWL6 | 2152 | KIRREL2 | Kin of IRRE-like pr | 489 | 1 | 489 | 0.040899796 | 0 | 0.020449898 |
| P01135 | 2153 | TGFA | Protransforming g | 74 | 1 | 74 | 0.040540541 | 0.067567568 | 0.054054054 |
| Q9P273 | 2154 | TENM3 | Teneurin-3 (Ten- | 2367 | 1 | 2367 | 0.033375581 | 0.008449514 | 0.020912548 |
| Q86YD3 | 2155 | TMEM25 | Transmembrane p | 205 | 1 | 205 | 0.058536585 | 0.009756098 | 0.034146341 |
| Q6IEE7 | 2156 | TMEM132E | Transmembrane p | 867 | 1 | 867 | 0.056516724 | 0.031141869 | 0.043829296 |
| O43493 | 2157 | TGOLN2 | Trans-Golgi netw | 359 | 1 | 359 | 0.094707521 | 0.245125348 | 0.169916435 |
| P08138 | 2158 | NGFR | Tumor necrosis fa | 221 | 1 | 221 | 0.081447964 | 0.099547511 | 0.090497738 |
| Q9Y6Q6 | 2159 | TNFRSF11A | Tumor necrosis fa | 182 | 1 | 182 | 0.043956044 | 0 | 0.021978022 |
| P25445 | 2160 | FAS | Tumor necrosis fa | 147 | 1 | 147 | 0.034013605 | 0.074829932 | 0.054421769 |
| O14788 | 2161 | TNFSF11 | Tumor necrosis fa | 248 | 1 | 248 | 0.072580645 | 0.012096774 | 0.04233871 |
| Q6ZMR5 | 2162 | TMPRSS11A | Transmembrane p | 381 | 1 | 381 | 0.020997375 | 0.028871391 | 0.024934383 |

| | | | | | | | | | |
|---|---|---|---|---|---|---|---|---|---|
| P48023 | 2163 | FASLG | Tumor necrosis fa | 178 | 1 | 178 | 0.04494382 | 0.039325843 | 0.042134831 |
| O60603 | 2164 | TLR2 | Toll-like receptor | 567 | 1 | 567 | 0 | 0.014109347 | 0.007054674 |
| Q96J42 | 2165 | TXNDC15 | Thioredoxin doma | 288 | 1 | 288 | 0.038194444 | 0.076388889 | 0.057291667 |
| Q9UPZ6 | 2166 | THSD7A | Thrombospondin | 1559 | 1 | 1559 | 0.036561899 | 0.089801155 | 0.063181527 |
| Q07011 | 2167 | TNFRSF9 | Tumor necrosis fa | 162 | 1 | 162 | 0.055555556 | 0.092592593 | 0.074074074 |
| O95185 | 2168 | UNC5C | Netrin receptor UI | 339 | 1 | 339 | 0.029498525 | 0.002949853 | 0.016224189 |
| Q9GZP7 | 2169 | VN1R1 | Vomeronasal type | 123 | 4 | 55 | 0.06504065 | 0.008130081 | 0.036585366 |
| P35916 | 2170 | FLT4 | Vascular endothe | 750 | 1 | 750 | 0.029333333 | 0.004 | 0.016666667 |
| Q9Y6M5 | 2171 | SLC30A1 | Zinc transporter 1 | 54 | 3 | 38 | 0.037037037 | 0 | 0.018518519 |
| Q9NYW7 | 2172 | TAS2R1 | Taste receptor typ | 57 | 4 | 32 | 0.01754386 | 0.035087719 | 0.026315789 |
| Q03167 | 2173 | TGFBR3 | Transforming grov | 766 | 1 | 766 | 0.019582245 | 0.006527415 | 0.01305483 |
| Q6N022 | 2174 | TENM4 | Teneurin-4 (Ten- | 2402 | 1 | 2402 | 0.030391341 | 0.01623647 | 0.023313905 |
| Q86T26 | 2175 | TMPRSS11B | Transmembrane p | 377 | 1 | 377 | 0.034482759 | 0.039787798 | 0.037135279 |
| Q9ULQ1 | 2176 | TPCN1 | Two pore calcium | 84 | 7 | 34 | 0.071428571 | 0 | 0.035714286 |
| P01375 | 2177 | TNF | Tumor necrosis fa | 176 | 1 | 176 | 0.028409091 | 0.039772727 | 0.034090909 |
| Q13507 | 2178 | TRPC3 | Short transient re | 125 | 3 | 50 | 0.04 | 0.016 | 0.028 |
| O95857 | 2179 | TSPAN13 | Tetraspanin-13 (T | 76 | 2 | 73 | 0.026315789 | 0.039473684 | 0.032894737 |
| Q8NBL3 | 2180 | TMEM178A | Transmembrane p | 180 | 2 | 153 | 0.005555556 | 0.033333333 | 0.019444444 |
| Q9NYV7 | 2181 | TAS2R16 | Taste receptor typ | 63 | 3 | 35 | 0.031746032 | 0.031746032 | 0.031746032 |
| P0DPE3 | 2182 | TMDD1 | Transmembrane a | 190 | 1 | 190 | 0.021052632 | 0.042105263 | 0.031578947 |
| Q969Z4 | 2183 | RELT | Tumor necrosis fa | 136 | 1 | 136 | 0.066176471 | 0.080882353 | 0.073529412 |
| P19438 | 2184 | TNFRSF1A | Tumor necrosis fa | 181 | 1 | 181 | 0.038674033 | 0.016574586 | 0.027624309 |
| Q7Z4N2 | 2185 | TRPM1 | Transient recepto | 85 | 3 | 45 | 0 | 0 | 0 |
| Q8NFZ6 | 2186 | VN1R2 | Vomeronasal type | 118 | 4 | 55 | 0.042372881 | 0.016949153 | 0.029661017 |
| Q6UX27 | 2187 | VSTM1 | V-set and transme | 118 | 1 | 118 | 0 | 0.033898305 | 0.016949153 |
| Q86TG1 | 2188 | TMEM150A | Transmembrane p | 72 | 3 | 51 | 0.027777778 | 0.027777778 | 0.027777778 |
| P57738 | 2189 | TCTA | T-cell leukemia tra | 47 | 2 | 41 | 0 | 0.127659574 | 0.063829787 |
| Q9NR96 | 2190 | TLR9 | Toll-like receptor | 792 | 1 | 792 | 0 | 0.021464646 | 0.010732323 |
| Q9Y493 | 2191 | ZAN | Zonadhesin | 2739 | 1 | 2739 | 0.082876962 | 0.085067543 | 0.083972253 |
| Q401N2 | 2192 | ZACN | Zinc-activated liga | 240 | 3 | 207 | 0 | 0.029166667 | 0.014583333 |
| O00322 | 2193 | UPK1A | Uroplakin-1a (UP | 140 | 2 | 117 | 0.085714286 | 0.042857143 | 0.064285714 |
| O75445 | 2194 | USH2A | Usherin (Usher sy | 5010 | 1 | 5010 | 0.085828343 | 0.031137725 | 0.058483034 |
| Q6H3X3 | 2195 | RAET1G | UL-16 binding pro | 197 | 1 | 197 | 0 | 0.045685279 | 0.02284264 |
| Q05996 | 2196 | ZP2 | Zona pellucida sp | 677 | 1 | 677 | 0.042836041 | 0.031019202 | 0.036927622 |
| Q7Z5H4 | 2197 | VN1R5 | Vomeronasal type | 29 | 4 | 11 | 0.034482759 | 0 | 0.017241379 |
| P47901 | 2198 | AVPR1B | Vasopressin V1b r | 92 | 4 | 34 | 0.076086957 | 0.065217391 | 0.070652174 |
| O43657 | 2199 | TSPAN6 | Tetraspanin-6 (Ts | 111 | 2 | 93 | 0.018018018 | 0.036036036 | 0.027027027 |
| B0FP48 | 2200 | UPK3BL1 | Uroplakin-3b-like | 170 | 1 | 170 | 0.058823529 | 0.058823529 | 0.058823529 |
| Q06418 | 2201 | TYRO3 | Tyrosine-protein k | 388 | 1 | 388 | 0.06185567 | 0.033505155 | 0.047680412 |
| Q8NFF2 | 2202 | SLC24A4 | Sodium/potassiur | 91 | 5 | 58 | 0.054945055 | 0.054945055 | 0.054945055 |
| Q16620 | 2203 | NTRK2 | BDNF/NT-3 grow | 398 | 1 | 398 | 0.055276382 | 0.030150754 | 0.042713568 |
| Q9HDB5 | 2204 | NRXN3 | Neurexin-3-beta ( | 526 | 1 | 526 | 0.114068441 | 0.178707224 | 0.146387833 |
| Q96EP9 | 2205 | SLC10A4 | Sodium/bile acid | 196 | 4 | 102 | 0.096938776 | 0.107142857 | 0.102040816 |
| Q8WWG1 | 2206 | NRG4 | Pro-neuregulin-4, | 61 | 1 | 61 | 0 | 0.032786885 | 0.016393443 |
| Q7Z442 | 2207 | PKD1L2 | Polycystic kidney | 1643 | 7 | 1326 | 0.04686549 | 0.024345709 | 0.0356056 |
| Q9H310 | 2208 | RHBG | Ammonium transp | 84 | 5 | 26 | 0.023809524 | 0.035714286 | 0.029761905 |
| Q8IUW5 | 2209 | RELL1 | RELT-like protein | 33 | 1 | 33 | 0.333333333 | 0.333333333 | 0.333333333 |
| P49190 | 2210 | PTH2R | Parathyroid horm | 186 | 4 | 118 | 0.032258065 | 0.021505376 | 0.02688172 |
| P48146 | 2211 | NPBWR2 | Neuropeptides B/ | 92 | 4 | 44 | 0.108695652 | 0.097826087 | 0.10326087 |
| Q8NGL6 | 2212 | OR4A15 | Olfactory receptor | 118 | 4 | 52 | 0.050847458 | 0.033898305 | 0.042372881 |
| Q8NH08 | 2213 | OR10AC1 | Olfactory receptor | 70 | 4 | 25 | 0.071428571 | 0.014285714 | 0.042857143 |
| Q8NGX5 | 2214 | OR10K1 | Olfactory receptor | 95 | 4 | 36 | 0.021052632 | 0.021052632 | 0.021052632 |
| Q8NGC9 | 2215 | OR11H4 | Olfactory receptor | 105 | 4 | 36 | 0.047619048 | 0 | 0.023809524 |
| Q8NGY0 | 2216 | OR10X1 | Olfactory receptor | 108 | 4 | 40 | 0.018518519 | 0.018518519 | 0.018518519 |
| Q8NGC8 | 2217 | OR11H7 | Olfactory receptor | 83 | 4 | 37 | 0.060240964 | 0.012048193 | 0.036144578 |
| Q15615 | 2218 | OR4D1 | Olfactory receptor | 90 | 4 | 36 | 0.033333333 | 0.011111111 | 0.022222222 |
| O95371 | 2219 | OR2C1 | Olfactory receptor | 93 | 4 | 37 | 0.021505376 | 0.010752688 | 0.016129032 |
| Q8N0Y5 | 2220 | OR8I2 | Olfactory receptor | 94 | 4 | 35 | 0.074468085 | 0.010638298 | 0.042553191 |
| Q6IF42 | 2221 | OR2A2 | Olfactory receptor | 91 | 4 | 36 | 0.032967033 | 0.010989011 | 0.021978022 |
| Q8NG85 | 2222 | OR2L3 | Olfactory receptor | 91 | 4 | 36 | 0.054945055 | 0.021978022 | 0.038461538 |
| P55085 | 2223 | F2RL1 | Proteinase-activa | 73 | 4 | 34 | 0.054794521 | 0.109589041 | 0.082191781 |
| O60486 | 2224 | PLXNC1 | Plexin-C1 (Virus-e | 909 | 1 | 909 | 0.051705171 | 0.04730473 | 0.04950495 |
| Q6UWV2 | 2225 | MPZL3 | Myelin protein zer | 126 | 1 | 126 | 0.087301587 | 0.023809524 | 0.055555556 |
| A6NM76 | 2226 | OR6C76 | Olfactory receptor | 73 | 4 | 31 | 0.054794521 | 0.01369863 | 0.034246575 |
| Q8NH57 | 2227 | OR52P1P | Putative olfactory | 98 | 4 | 35 | 0.040816327 | 0.010204082 | 0.025510204 |
| Q8NH07 | 2228 | OR11H2 | Olfactory receptor | 103 | 4 | 43 | 0.038834951 | 0.019417476 | 0.029126214 |
| Q8NGI6 | 2229 | OR4D10 | Olfactory receptor | 90 | 4 | 36 | 0.022222222 | 0.011111111 | 0.016666667 |
| Q8NH10 | 2230 | OR8U1 | Olfactory receptor | 95 | 4 | 36 | 0.031578947 | 0.010526316 | 0.021052632 |
| P47884 | 2231 | OR1D4 | Olfactory receptor | 91 | 4 | 37 | 0.021978022 | 0.010989011 | 0.016483516 |
| Q8NGR9 | 2232 | OR1N2 | Olfactory receptor | 110 | 4 | 41 | 0.027272727 | 0.009090909 | 0.018181818 |
| Q9H210 | 2233 | OR2D2 | Olfactory receptor | 90 | 4 | 36 | 0.044444444 | 0.011111111 | 0.027777778 |
| Q8NGP2 | 2234 | OR8J1 | Olfactory receptor | 96 | 4 | 36 | 0.052083333 | 0.020833333 | 0.036458333 |
| Q8NGG5 | 2235 | OR8K1 | Olfactory receptor | 101 | 4 | 36 | 0.01980198 | 0.00990099 | 0.014851485 |
| Q8NHC8 | 2236 | OR2T6 | Olfactory receptor | 95 | 4 | 36 | 0.031578947 | 0.010526316 | 0.021052632 |
| Q96RI0 | 2237 | F2RL3 | Proteinase-activa | 102 | 4 | 34 | 0.019607843 | 0.029411765 | 0.024509804 |
| Q15391 | 2238 | P2RY14 | P2Y purinoceptor | 96 | 4 | 28 | 0.072916667 | 0.072916667 | 0.072916667 |
| P16389 | 2239 | KCNA2 | Potassium voltage | 70 | 4 | 38 | 0.142857143 | 0 | 0.071428571 |
| O60309 | 2240 | LRRC37A3 | Leucine-rich repe | 1545 | 1 | 1545 | 0.065372168 | 0.132038835 | 0.098705502 |
| O75096 | 2241 | LRP4 | Low-density lipop | 1704 | 1 | 1704 | 0 | 0.031690141 | 0.01584507 |
| Q9UBY5 | 2242 | LPAR3 | Lysophosphatidic | 74 | 4 | 30 | 0.027027027 | 0.013513514 | 0.02027027 |
| Q9H1C0 | 2243 | LPAR5 | Lysophosphatidic | 88 | 4 | 29 | 0.079545455 | 0.102272727 | 0.090909091 |
| P21439 | 2244 | ABCB4 | Phosphatidylcholi | 100 | 5 | 44 | 0.04 | 0 | 0.02 |
| Q6IFN5 | 2245 | OR7E24 | Olfactory receptor | 113 | 4 | 42 | 0.03539823 | 0.008849558 | 0.022123894 |
| Q9NZ53 | 2246 | PODXL2 | Podocalyxin-like p | 467 | 1 | 467 | 0.062098501 | 0.124197002 | 0.093147752 |
| P08575 | 2247 | PTPRC | Receptor-type tyr | 551 | 1 | 551 | 0.170598911 | 0.121597096 | 0.146098004 |
| Q15256 | 2248 | PTPRR | Receptor-type tyr | 205 | 1 | 205 | 0.009756098 | 0 | 0.004878049 |
| Q9NSD7 | 2249 | RXFP3 | Relaxin-3 recepto | 133 | 4 | 80 | 0.037593985 | 0.067669173 | 0.052631579 |
| Q9ULC0 | 2250 | EMCN | Endomucin (Endo | 171 | 1 | 171 | 0.251461988 | 0.362573099 | 0.307017544 |
| Q8NET5 | 2251 | NFAM1 | NFAT activation n | 120 | 1 | 120 | 0.083333333 | 0.016666667 | 0.05 |
| Q14957 | 2252 | GRIN2C | Glutamate recept | 704 | 2 | 534 | 0.03125 | 0.017045455 | 0.024147727 |
| Q8NGH5 | 2253 | OR56A1 | Olfactory receptor | 103 | 4 | 35 | 0.058252427 | 0.038834951 | 0.048543689 |
| Q8TCB6 | 2254 | OR51E1 | Olfactory receptor | 98 | 4 | 35 | 0.030612245 | 0.020408163 | 0.025510204 |
| Q8NGD5 | 2255 | OR4K14 | Olfactory receptor | 90 | 4 | 36 | 0.022222222 | 0.011111111 | 0.016666667 |
| O60412 | 2256 | OR7C2 | Olfactory receptor | 95 | 4 | 36 | 0.063157895 | 0.010526316 | 0.036842105 |
| Q8NG98 | 2257 | OR7D4 | Olfactory receptor | 95 | 4 | 36 | 0.031578947 | 0 | 0.015789474 |
| Q8NG75 | 2258 | OR5T1 | Olfactory receptor | 106 | 4 | 36 | 0.028301887 | 0.009433962 | 0.018867925 |
| Q8N162 | 2259 | OR8H2 | Olfactory receptor | 95 | 4 | 36 | 0.052631579 | 0.010526316 | 0.031578947 |
| O95007 | 2260 | OR6B1 | Olfactory receptor | 94 | 4 | 35 | 0.053191489 | 0.031914894 | 0.042553191 |
| P47883 | 2261 | OR3A4P | Putative olfactory | 96 | 4 | 37 | 0.03125 | 0.010416667 | 0.020833333 |
| Q8NH16 | 2262 | OR2L2 | Olfactory receptor | 91 | 4 | 36 | 0.054945055 | 0.021978022 | 0.038461538 |
| Q9BQ51 | 2263 | PDCD1LG2 | Programmed cell | 200 | 1 | 200 | 0.065 | 0.01 | 0.0375 |
| O94856 | 2264 | NFASC | Neurofascin | 1192 | 1 | 1192 | 0.071308725 | 0.037751678 | 0.054530201 |
| Q92692 | 2265 | NECTIN2 | Nectin-2 (Herpes | 328 | 1 | 328 | 0.082317073 | 0.018292683 | 0.050304878 |
| Q8NGI2 | 2266 | OR52N4 | Olfactory receptor | 99 | 4 | 35 | 0.02020202 | 0.01010101 | 0.015151515 |
| Q96R47 | 2267 | OR2A14 | Olfactory receptor | 91 | 4 | 36 | 0.054945055 | 0.010989011 | 0.032967033 |
| Q6IF99 | 2268 | OR10K2 | Olfactory receptor | 95 | 4 | 36 | 0.021052632 | 0.021052632 | 0.021052632 |
| Q96RB7 | 2269 | OR5M11 | Olfactory receptor | 93 | 4 | 35 | 0.086021505 | 0.010752688 | 0.048387097 |
| Q8NGV6 | 2270 | OR5H6 | Olfactory receptor | 110 | 4 | 40 | 0.036363636 | 0.009090909 | 0.022727273 |
| O43869 | 2271 | OR2T1 | Olfactory receptor | 141 | 4 | 75 | 0.021276596 | 0.007092199 | 0.014184397 |
| Q15612 | 2272 | OR1Q1 | Olfactory receptor | 92 | 4 | 36 | 0.054347826 | 0.010869565 | 0.032608696 |
| Q6IFH4 | 2273 | OR6B2 | Olfactory receptor | 94 | 4 | 35 | 0.042553191 | 0.010638298 | 0.026595745 |
| Q8NGG0 | 2274 | OR8J3 | Olfactory receptor | 95 | 4 | 36 | 0.052631579 | 0.010526316 | 0.031578947 |
| Q8NGQ3 | 2275 | OR1S2 | Olfactory receptor | 105 | 4 | 37 | 0.038095238 | 0.00952381 | 0.023809524 |
| Q96R45 | 2276 | OR2A7 | Olfactory receptor | 91 | 4 | 36 | 0.032967033 | 0.010989011 | 0.021978022 |

| | | | | | | | | | |
|---|---|---|---|---|---|---|---|---|---|
| Q8NG83 | 2277 | OR2M3 | Olfactory receptor | 92 | 4 | 36 | 0.076086957 | 0.010869565 | 0.043478261 |
| Q92508 | 2278 | PIEZO1 | Piezo-type mecha | 233 | 1 | 233 | 0.025751073 | 0.004291845 | 0.015021459 |
| Q8NFZ3 | 2279 | NLGN4Y | Neuroligin-4, Y-lir | 632 | 1 | 632 | 0.037974684 | 0.041139241 | 0.039556962 |
| Q6IF63 | 2280 | OR52W1 | Olfactory receptor | 102 | 4 | 35 | 0.039215686 | 0.009803922 | 0.024509804 |
| Q8NH19 | 2281 | OR10AG1 | Olfactory receptor | 86 | 4 | 36 | 0.011627907 | 0.011627907 | 0.011627907 |
| Q8NGN5 | 2282 | OR10G8 | Olfactory receptor | 92 | 4 | 36 | 0.065217391 | 0.010869565 | 0.038043478 |
| Q8NGN8 | 2283 | OR4A4P | Putative olfactory | 88 | 4 | 36 | 0.034090909 | 0.022727273 | 0.028409091 |
| P0C604 | 2284 | OR4A8 | Olfactory receptor | 78 | 4 | 32 | 0.012820513 | 0.012820513 | 0.012820513 |
| O76000 | 2285 | OR2B3 | Putative olfactory | 92 | 4 | 36 | 0.010869565 | 0.010869565 | 0.010869565 |
| P58173 | 2286 | OR2B6 | Olfactory receptor | 92 | 4 | 36 | 0.032608696 | 0.02173913 | 0.027173913 |
| Q8NGA0 | 2287 | OR7G1 | Olfactory receptor | 90 | 4 | 41 | 0.033333333 | 0.022222222 | 0.027777778 |
| Q8NGE7 | 2288 | OR9K2 | Olfactory receptor | 120 | 4 | 49 | 0.025 | 0.025 | 0.025 |
| Q8NGG3 | 2289 | OR5T3 | Olfactory receptor | 124 | 4 | 54 | 0.056451613 | 0.016129032 | 0.036290323 |
| Q8NGZ6 | 2290 | OR6F1 | Olfactory receptor | 95 | 4 | 36 | 0.031578947 | 0.010526316 | 0.021052632 |
| Q8NGD1 | 2291 | OR4N2 | Olfactory receptor | 90 | 4 | 36 | 0.022222222 | 0.011111111 | 0.016666667 |
| P47893 | 2292 | OR3A2 | Olfactory receptor | 101 | 4 | 37 | 0.03960396 | 0.00990099 | 0.024752475 |
| Q8NGI9 | 2293 | OR5A2 | Olfactory receptor | 101 | 4 | 41 | 0.02970297 | 0.00990099 | 0.01980198 |
| P04000 | 2294 | OPN1LW | Long-wave-sensit | 96 | 4 | 51 | 0 | 0.104166667 | 0.052083333 |
| Q6IEZ7 | 2295 | OR2T5 | Olfactory receptor | 96 | 4 | 36 | 0.052083333 | 0 | 0.026041667 |
| Q8NHB1 | 2296 | OR2V1 | Olfactory receptor | 88 | 4 | 30 | 0.011363636 | 0.011363636 | 0.011363636 |
| Q8NFJ6 | 2297 | PROKR2 | Prokineticin recep | 126 | 4 | 53 | 0.166666667 | 0.015873016 | 0.091269841 |
| P47804 | 2298 | RGR | RPE-retinal G prot | 60 | 4 | 23 | 0 | 0.05 | 0.025 |
| Q9HCL0 | 2299 | PCDH18 | Protocadherin-18 | 671 | 1 | 671 | 0.037257824 | 0.013412817 | 0.02533532 |
| Q9Y5H6 | 2300 | PCDHA8 | Protocadherin alp | 667 | 1 | 667 | 0.017991004 | 0.005997001 | 0.011994003 |
| Q9Y3Q0 | 2301 | NAALAD2 | N-acetylated-alph | 708 | 1 | 708 | 0 | 0.018361582 | 0.009180791 |
| Q8NGA6 | 2302 | OR10H5 | Olfactory receptor | 95 | 4 | 36 | 0.031578947 | 0.010526316 | 0.021052632 |
| Q8NGX6 | 2303 | OR10R2 | Olfactory receptor | 115 | 4 | 44 | 0.017391304 | 0 | 0.008695652 |
| Q8NGN7 | 2304 | OR10D4P | Putative olfactory | 91 | 4 | 36 | 0.054945055 | 0.021978022 | 0.038461538 |
| Q8NGJ1 | 2305 | OR4D6 | Olfactory receptor | 90 | 4 | 36 | 0.022222222 | 0 | 0.011111111 |
| P0C623 | 2306 | OR4Q2 | Olfactory receptor | 78 | 4 | 30 | 0.025641026 | 0.012820513 | 0.019230769 |
| Q8NH83 | 2307 | OR4A5 | Olfactory receptor | 87 | 4 | 35 | 0.034482759 | 0.011494253 | 0.022988506 |
| P58170 | 2308 | OR1D5 | Olfactory receptor | 91 | 4 | 37 | 0.021978022 | 0.010989011 | 0.016483516 |
| Q8NGJ0 | 2309 | OR5A1 | Olfactory receptor | 95 | 4 | 36 | 0.052631579 | 0.010526316 | 0.031578947 |
| Q8NGY2 | 2310 | OR6K2 | Olfactory receptor | 96 | 4 | 38 | 0.03125 | 0.010416667 | 0.020833333 |
| Q8NH92 | 2311 | OR1S1 | Olfactory receptor | 105 | 4 | 37 | 0.057142857 | 0.00952381 | 0.033333333 |
| Q8NGG8 | 2312 | OR8B3 | Olfactory receptor | 94 | 4 | 36 | 0.021276596 | 0 | 0.010638298 |
| Q8NGZ4 | 2313 | OR2G3 | Olfactory receptor | 92 | 4 | 36 | 0.043478261 | 0.010869565 | 0.027173913 |
| Q8NGY9 | 2314 | OR2L8 | Olfactory receptor | 91 | 4 | 36 | 0.10989011 | 0.021978022 | 0.065934066 |
| Q8NGN2 | 2315 | OR10S1 | Olfactory receptor | 107 | 4 | 37 | 0.074766355 | 0.009345794 | 0.042056075 |
| Q9Y5G2 | 2316 | PCDHGB2 | Protocadherin gar | 660 | 1 | 660 | 0.031818182 | 0.004545455 | 0.018181818 |
| Q9Y5E9 | 2317 | PCDHB14 | Protocadherin bet | 659 | 1 | 659 | 0.040971168 | 0.009104704 | 0.025037936 |
| Q9Y5I0 | 2318 | PCDHA13 | Protocadherin alp | 667 | 1 | 667 | 0.068965517 | 0.010494753 | 0.039730135 |
| P51805 | 2319 | PLXNA3 | Plexin-A3 (Plexin- | 1200 | 1 | 1200 | 0.039166667 | 0.0225 | 0.030833333 |
| Q9HBL6 | 2320 | LRTM1 | Leucine-rich repe | 260 | 1 | 260 | 0.034615385 | 0.038461538 | 0.036538462 |
| Q99650 | 2321 | OSMR | Oncostatin-M-spe | 712 | 1 | 712 | 0.051966292 | 0.037921348 | 0.04494382 |
| Q8TDS5 | 2322 | OXER1 | Oxoeicosanoid re | 156 | 4 | 96 | 0.102564103 | 0.128205128 | 0.115384615 |
| Q6NUS6 | 2323 | TCTN3 | Tectonic-3 | 558 | 1 | 558 | 0.062724014 | 0.05734767 | 0.060035842 |
| Q9NZC2 | 2324 | TREM2 | Triggering recepto | 155 | 1 | 155 | 0.04516129 | 0.032258065 | 0.038709677 |
| O60235 | 2325 | TMPRSS11D | Transmembrane p | 376 | 1 | 376 | 0.087765957 | 0.034574468 | 0.061170213 |
| Q15399 | 2326 | TLR1 | Toll-like receptor | 555 | 1 | 555 | 0 | 0.025225225 | 0.012612613 |
| Q9NYK1 | 2327 | TLR7 | Toll-like receptor | 812 | 1 | 812 | 0 | 0.030788177 | 0.015394089 |
| Q9NQA5 | 2328 | TRPV5 | Transient recepto | 41 | 2 | 36 | 0.024390244 | 0.024390244 | 0.024390244 |
| O95858 | 2329 | TSPAN15 | Tetraspanin-15 (T | 137 | 2 | 120 | 0.03649635 | 0.02189781 | 0.02919708 |
| A6NDX4 | 2330 | 0 | Putative transmer | 100 | 1 | 100 | 0.09 | 0.05 | 0.07 |
| Q9Y5U5 | 2331 | TNFRSF18 | Tumor necrosis fa | 136 | 1 | 136 | 0.036764706 | 0.066176471 | 0.051470588 |
| Q9UL62 | 2332 | TRPC5 | Short transient re | 126 | 3 | 69 | 0.015873016 | 0.007936508 | 0.011904762 |
| P07204 | 2333 | THBD | Thrombomodulin | 496 | 1 | 496 | 0.04233871 | 0.0625 | 0.052419355 |
| O43557 | 2334 | TNFSF14 | Tumor necrosis fa | 181 | 1 | 181 | 0.027624309 | 0.022099448 | 0.024861878 |
| Q9BSN7 | 2335 | TMEM204 | Transmembrane p | 88 | 2 | 76 | 0.011363636 | 0.022727273 | 0.017045455 |
| O60602 | 2336 | TLR5 | Toll-like receptor | 618 | 1 | 618 | 0 | 0.022653722 | 0.011326861 |
| Q5DID0 | 2337 | UMODL1 | Uromodulin-like 1 | 1250 | 1 | 1250 | 0.0592 | 0.0672 | 0.0632 |
| Q7Z5H5 | 2338 | VN1R4 | Vomeronasal type | 67 | 4 | 37 | 0.029850746 | 0.029850746 | 0.029850746 |
| P32241 | 2339 | VIPR1 | Vasoactive intesti | 158 | 4 | 111 | 0.044303797 | 0.03164557 | 0.037974684 |
| P35968 | 2340 | KDR | Vascular endothe | 744 | 1 | 744 | 0.056451613 | 0.02688172 | 0.041666667 |
| Q8TD43 | 2341 | TRPM4 | Transient recepto | 56 | 4 | 34 | 0.035714286 | 0.035714286 | 0.035714286 |
| P41587 | 2342 | VIPR2 | Vasoactive intesti | 153 | 4 | 102 | 0.052287582 | 0.032679739 | 0.04248366 |
| Q96BF3 | 2343 | TMIGD2 | Transmembrane a | 127 | 1 | 127 | 0.023622047 | 0.023622047 | 0.023622047 |
| O00220 | 2344 | TNFRSF10A | Tumor necrosis fa | 215 | 1 | 215 | 0.074418605 | 0.125581395 | 0.1 |
| P20333 | 2345 | TNFRSF1B | Tumor necrosis fa | 234 | 1 | 234 | 0.111111111 | 0.162393162 | 0.136752137 |
| Q93038 | 2346 | TNFRSF25 | Tumor necrosis fa | 174 | 1 | 174 | 0.040229885 | 0.011494253 | 0.025862069 |
| Q4V9L6 | 2347 | TMEM119 | Transmembrane p | 70 | 1 | 70 | 0.157142857 | 0.185714286 | 0.171428571 |
| P28908 | 2348 | TNFRSF8 | Tumor necrosis fa | 366 | 1 | 366 | 0.213114754 | 0.161202186 | 0.18715847 |
| Q9Y5S1 | 2349 | TRPV2 | Transient recepto | 22 | 1 | 22 | 0 | 0 | 0 |
| Q7RTX0 | 2350 | TAS1R3 | Taste receptor typ | 593 | 4 | 549 | 0.02529511 | 0.02698145 | 0.02613828 |
| Q86VR7 | 2351 | VSIG10L | V-set and immunc | 748 | 1 | 748 | 0 | 0.018716578 | 0.009358289 |
| P25942 | 2352 | CD40 | Tumor necrosis fa | 172 | 1 | 172 | 0.023255814 | 0.087209302 | 0.055232558 |
| Q9NV12 | 2353 | TMEM140 | Transmembrane p | 58 | 2 | 44 | 0.068965517 | 0.017241379 | 0.043103448 |
| P32971 | 2354 | TNFSF8 | Tumor necrosis fa | 171 | 1 | 171 | 0.040935673 | 0.029239766 | 0.035087719 |
| Q7Z404 | 2355 | TMC4 | Transmembrane c | 330 | 5 | 167 | 0.027272727 | 0.006060606 | 0.016666667 |
| P0DKB5 | 2356 | TPBGL | Trophoblast glyco | 280 | 1 | 280 | 0.010714286 | 0.017857143 | 0.014285714 |
| Q7Z7D3 | 2357 | VTCN1 | V-set domain-con | 234 | 1 | 234 | 0 | 0.025641026 | 0.012820513 |
| O14836 | 2358 | TNFRSF13B | Tumor necrosis fa | 164 | 1 | 164 | 0.012195122 | 0.036585366 | 0.024390244 |
| Q9BYE2 | 2359 | TMPRSS13 | Transmembrane p | 399 | 1 | 399 | 0.032581454 | 0.05764411 | 0.045112782 |
| O43508 | 2360 | TNFSF12 | Tumor necrosis fa | 206 | 1 | 206 | 0.014563107 | 0.029126214 | 0.02184466 |
| O60636 | 2361 | TSPAN2 | Tetraspanin-2 (Ts | 95 | 2 | 76 | 0.042105263 | 0.021052632 | 0.031578947 |
| Q8IW00 | 2362 | VSTM4 | V-set and transme | 156 | 1 | 156 | 0 | 0.038461538 | 0.019230769 |
| P98155 | 2363 | VLDLR | Very low-density l | 769 | 1 | 769 | 0.031209363 | 0.058517555 | 0.044863459 |
| P60852 | 2364 | ZP1 | Zona pellucida sp | 575 | 1 | 575 | 0.071304348 | 0.069565217 | 0.070434783 |
| P07550 | 2365 | ADRB2 | Beta-2 adrenergic | 70 | 4 | 33 | 0.057142857 | 0.071428571 | 0.064285714 |
| Q92482 | 2366 | AQP3 | Aquaporin-3 (AQF | 54 | 3 | 33 | 0.055555556 | 0.018518519 | 0.037037037 |
| P55087 | 2367 | AQP4 | Aquaporin-4 (AQF | 39 | 4 | 18 | 0.051282051 | 0.051282051 | 0.051282051 |
| P21730 | 2368 | C5AR1 | C5a anaphylatoxi | 93 | 4 | 36 | 0.043010753 | 0.075268817 | 0.059139785 |
| Q8IXH8 | 2369 | CDH26 | Cadherin-like prot | 586 | 1 | 586 | 0.04778157 | 0.005119454 | 0.026450512 |
| P04234 | 2370 | CD3D | T-cell surface glyc | 83 | 1 | 83 | 0 | 0.036144578 | 0.018072289 |
| P55285 | 2371 | CDH6 | Cadherin-6 (Kidne | 561 | 1 | 561 | 0.048128342 | 0.007130125 | 0.027629234 |
| P42658 | 2372 | DPP6 | Dipeptidyl aminop | 748 | 1 | 748 | 0.038770053 | 0.016042781 | 0.027406417 |
| Q15768 | 2373 | EFNB3 | Ephrin-B3 (EPH-re | 198 | 1 | 198 | 0.03030303 | 0.04040404 | 0.035353535 |
| Q7Z407 | 2374 | CSMD3 | CUB and sushi do | 3566 | 1 | 3566 | 0.049635446 | 0.068424004 | 0.059029725 |
| Q14126 | 2375 | DSG2 | Desmoglein-2 (Ca | 559 | 1 | 559 | 0.02862254 | 0.012522361 | 0.020572451 |
| Q30154 | 2376 | HLA-DRB5 | HLA class II histo | 197 | 1 | 197 | 0.020304569 | 0.010152284 | 0.015228426 |
| P01906 | 2377 | HLA-DQA2 | HLA class II histo | 193 | 1 | 193 | 0.046632124 | 0.03626943 | 0.041450777 |
| P01909 | 2378 | HLA-DQA1 | HLA class II histo | 192 | 1 | 192 | 0.041666667 | 0.041666667 | 0.041666667 |
| Q9HCC8 | 2379 | GDPD2 | Glycerophosphoin | 316 | 3 | 280 | 0.012658228 | 0.037974684 | 0.025316456 |
| Q6W3E5 | 2380 | GDPD4 | Glycerophosphodi | 302 | 2 | 284 | 0.006622517 | 0.016556291 | 0.011589404 |
| Q8WTR4 | 2381 | GDPD5 | Glycerophosphodi | 320 | 3 | 282 | 0.05 | 0.053125 | 0.0515625 |
| Q9NS67 | 2382 | GPR27 | Probable G-protei | 75 | 4 | 22 | 0.08 | 0.026666667 | 0.053333333 |
| O00270 | 2383 | GPR31 | 12-(S)-hydroxy-5, | 83 | 4 | 27 | 0 | 0.048192771 | 0.024096386 |
| P98172 | 2384 | EFNB1 | Ephrin-B1 (EFL-3) | 209 | 1 | 209 | 0.038277512 | 0.076555024 | 0.057416268 |
| Q49SQ1 | 2385 | GPR33 | Probable G-protei | 102 | 4 | 43 | 0.049019608 | 0.078431373 | 0.06372549 |
| P52799 | 2386 | EFNB2 | Ephrin-B2 (EPH-re | 201 | 1 | 201 | 0.089552239 | 0.089552239 | 0.089552239 |
| Q9BZJ7 | 2387 | GPR62 | G-protein coupled | 70 | 4 | 26 | 0 | 0.042857143 | 0.021428571 |
| Q9BZJ6 | 2388 | GPR63 | Probable G-protei | 134 | 4 | 80 | 0.156716418 | 0.104477612 | 0.130597015 |
| Q96B86 | 10000 | RGMA | Repulsive guidanc | 255 | 1 | 255 | 0.039215686 | 0.011764706 | 0.025490196 |
| P13987 | 10001 | CD59 | CD59 glycoprotein | 76 | 1 | 76 | 0.039473684 | 0.026315789 | 0.032894737 |

| | | | | | | | | | |
|---|---|---|---|---|---|---|---|---|---|
| P51654 | 10002 | GPC3 | Glypican-3 (GTR2 | 528 | 2 | 333 | 0.009469697 | 0.028409091 | 0.018939394 |
| P15328 | 10003 | FOLR1 | Folate receptor al | 209 | 1 | 209 | 0.028708134 | 0.023923445 | 0.026315789 |
| P13385 | 10004 | TDGF1 | Teratocarcinoma- | 119 | 1 | 119 | 0.016806723 | 0.042016807 | 0.029411765 |
| Q9BZM5 | 10005 | ULBP2 | UL16-binding pro | 190 | 1 | 190 | 0.031578947 | 0.026315789 | 0.028947368 |
| P16444 | 10006 | DPEP1 | Dipeptidase 1 (EC | 368 | 1 | 368 | 0.054347826 | 0.024456522 | 0.039402174 |
| P06731 | 10007 | CEACAM5 | Carcinoembryonic | 650 | 1 | 650 | 0.103076923 | 0.043076923 | 0.073076923 |
| P08582 | 10008 | MELTF | Melanotransferrin | 689 | 1 | 689 | 0.015965167 | 0.01161103 | 0.013788099 |
| P05187 | 10009 | ALPP | Alkaline phosphat | 483 | 1 | 483 | 0.039337474 | 0.026915114 | 0.033126294 |
| Q9ULZ9 | 10010 | MMP17 | Matrix metallopro | 439 | 1 | 439 | 0.011389522 | 0.027334852 | 0.019362187 |
| Q9NPA2 | 10011 | MMP25 | Matrix metallopro | 431 | 1 | 431 | 0.023201856 | 0.023201856 | 0.023201856 |
| P07911 | 10012 | UMOD | Uromodulin (Tam | 1151 | 2 | 589 | 0.050390964 | 0.051259774 | 0.050825369 |
| P22748 | 10013 | CA4 | Carbonic anhydra | 265 | 1 | 265 | 0.011320755 | 0.022641509 | 0.016981132 |
| O95868 | 10014 | LY6G6D | Lymphocyte antig | 84 | 1 | 84 | 0.035714286 | 0.023809524 | 0.029761905 |
| P0CG37 | 10015 | CFC1 | Cryptic protein (Cr | 132 | 1 | 132 | 0.022727273 | 0.075757576 | 0.049242424 |
| P40199 | 10016 | CEACAM6 | Carcinoembryonic | 285 | 1 | 285 | 0.09122807 | 0.045614035 | 0.068421053 |
| P14384 | 10017 | CPM | Carboxypeptidase | 405 | 1 | 405 | 0.022222222 | 0.014814815 | 0.018518519 |
| Q02246 | 10018 | CNTN2 | Contactin-2 (Axor | 981 | 1 | 981 | 0.060142712 | 0.02038736 | 0.040265036 |
| P08174 | 10019 | CD55 | Complement deca | 318 | 1 | 318 | 0.113207547 | 0.106918239 | 0.110062893 |
| P21589 | 10020 | NT5E | 5'-nucleotidase (5 | 522 | 1 | 522 | 0 | 0.009578544 | 0.004789272 |
| P31997 | 10021 | CEACAM8 | Carcinoembryonic | 285 | 1 | 285 | 0.098245614 | 0.038596491 | 0.068421053 |
| P25063 | 10022 | CD24 | Signal transducer | 32 | 1 | 32 | 0.53125 | 0.4375 | 0.484375 |
| P31358 | 10023 | CD52 | CAMPATH-1 anti | 11 | 1 | 11 | 0.181818182 | 0.454545455 | 0.318181818 |
| P35052 | 10024 | GPC1 | Glypican-1 [Cleav | 506 | 1 | 506 | 0.009881423 | 0.031620553 | 0.020750988 |
| P14207 | 10025 | FOLR2 | Folate receptor be | 213 | 1 | 213 | 0.014084507 | 0.042253521 | 0.028169014 |
| Q6UWR7 | 10026 | ENPP6 | Glycerophosphocl | 396 | 1 | 396 | 0.015151515 | 0.012626263 | 0.013888889 |
| Q9GZZ7 | 10027 | GFRA4 | GDNF family rece | 257 | 1 | 257 | 0.027237354 | 0.054474708 | 0.040856031 |
| O95867 | 10028 | LY6G6C | Lymphocyte antig | 80 | 1 | 80 | 0.025 | 0.05 | 0.0375 |
| Q13421 | 10029 | MSLN | Mesothelin (CAK1 | 1128 | 3 | 569 | 0.016843972 | 0.029255319 | 0.023049645 |
| Q9BZM4 | 10030 | ULBP3 | UL16-binding pro | 187 | 1 | 187 | 0.021390374 | 0.016042781 | 0.018716578 |
| Q9BZM6 | 10031 | ULBP1 | UL16-binding pro | 190 | 1 | 190 | 0.063157895 | 0.015789474 | 0.039473684 |
| P20827 | 10032 | EFNA1 | Ephrin-A1 (EPH-re | 163 | 1 | 163 | 0.006134969 | 0.036809816 | 0.021472393 |
| P08571 | 10033 | CD14 | Monocyte differer | 672 | 2 | 347 | 0.02827381 | 0.03125 | 0.029761905 |
| P55259 | 10034 | GP2 | Pancreatic secret | 483 | 1 | 483 | 0.026915114 | 0.043478261 | 0.035196687 |
| Q03405 | 10035 | PLAUR | Urokinase plasmir | 282 | 1 | 282 | 0.039007092 | 0.021276596 | 0.030141844 |
| P04216 | 10036 | THY1 | Thy-1 membrane | 110 | 1 | 110 | 0 | 0.036363636 | 0.018181818 |
| Q9BZR6 | 10037 | RTN4R | Reticulon-4 recep | 420 | 1 | 420 | 0.035714286 | 0.054761905 | 0.045238095 |
| Q5VY80 | 10038 | RAET1L | UL16-binding pro | 192 | 1 | 192 | 0.036458333 | 0.010416667 | 0.0234375 |
| Q86UN2 | 10039 | RTN4RL1 | Reticulon-4 recep | 395 | 1 | 395 | 0.035443038 | 0.055696203 | 0.04556962 |
| P04156 | 10040 | PRNP | Major prion protei | 207 | 1 | 207 | 0.053140097 | 0.024154589 | 0.038647343 |
| O75443 | 10041 | TECTA | Alpha-tectorin | 2068 | 1 | 2068 | 0.042553191 | 0.034816248 | 0.03868472 |
| Q6NW40 | 10042 | RGMB | Repulsive guidanc | 367 | 1 | 367 | 0.040871935 | 0.054495913 | 0.047683924 |
| Q9UKY0 | 10043 | PRND | Prion-like protein | 126 | 1 | 126 | 0.047619048 | 0.023809524 | 0.035714286 |
| Q86UN3 | 10044 | RTN4RL2 | Reticulon-4 recep | 343 | 1 | 343 | 0.034985423 | 0.037900875 | 0.036443149 |
| Q14982 | 10045 | OPCML | Opioid-binding pr | 294 | 1 | 294 | 0.040816327 | 0.020408163 | 0.030612245 |
| P10696 | 10046 | ALPG | Alkaline phosphat | 483 | 1 | 483 | 0.070393375 | 0.028985507 | 0.049689441 |
| P0DP58 | 10047 | LYNX1 | Ly-6/neurotoxin-l | 70 | 1 | 70 | 0 | 0 | 0 |
| O95498 | 10048 | VNN2 | Vascular non-infla | 470 | 1 | 470 | 0.085106383 | 0.021276596 | 0.053191489 |
| Q93070 | 10049 | ART4 | Ecto-ADP-ribosylt | 238 | 1 | 238 | 0 | 0.025210084 | 0.012605042 |
| Q6ZVN8 | 10050 | HJV | Hemojuvelin (Her | 364 | 1 | 364 | 0.035714286 | 0.032967033 | 0.034340659 |
| Q6UQ28 | 10051 | PLET1 | Placenta-express | 161 | 1 | 161 | 0.062111801 | 0.043478261 | 0.052795031 |
| Q6UWB4 | 10052 | PRSS55 | Serine protease 5 | 333 | 1 | 333 | 0.036036036 | 0.027027027 | 0.031531532 |
| Q8TDM5 | 10053 | SPACA4 | Sperm acrosome r | 77 | 1 | 77 | 0.077922078 | 0.012987013 | 0.045454545 |
| Q9BY14 | 10054 | TEX101 | Testis-expressed | 196 | 1 | 196 | 0.096938776 | 0.051020408 | 0.073979592 |
| Q5BIV9 | 10055 | SPRN | Shadow of prion p | 101 | 1 | 101 | 0.03960396 | 0.03960396 | 0.03960396 |
| Q6UWN0 | 10056 | LYPD4 | Ly6/PLAUR doma | 198 | 1 | 198 | 0 | 0.04040404 | 0.02020202 |
| Q7Z3B1 | 10057 | NEGR1 | Neuronal growth r | 286 | 1 | 286 | 0.083916084 | 0.020979021 | 0.052447552 |
| Q6UX82 | 10058 | LYPD8 | Ly6/PLAUR doma | 195 | 1 | 195 | 0 | 0.071794872 | 0.035897436 |
| Q7RTY9 | 10059 | PRSS41 | Serine protease 4 | 244 | 1 | 244 | 0.06557377 | 0.004098361 | 0.034836066 |
| P52961 | 10060 | ART1 | GPI-linked NAD(F | 272 | 1 | 272 | 0 | 0.022058824 | 0.011029412 |
| P05186 | 10061 | ALPL | Alkaline phosphat | 483 | 1 | 483 | 0.047619048 | 0.033126294 | 0.040372671 |
| O43280 | 10062 | TREH | Trehalase (EC 3.2 | 532 | 1 | 532 | 0.011278195 | 0.013157895 | 0.012218045 |
| Q96PL2 | 10063 | TECTB | Beta-tectorin | 287 | 1 | 287 | 0.020905923 | 0.013937282 | 0.017421603 |
| P09923 | 10064 | ALPI | Intestinal-type alk | 483 | 1 | 483 | 0.043478261 | 0.02484472 | 0.034161491 |
| O75326 | 10065 | SEMA7A | Semaphorin-7A (( | 603 | 1 | 603 | 0.014925373 | 0.028192371 | 0.021558872 |
| P55290 | 10066 | CDH13 | Cadherin-13 (Hea | 554 | 1 | 554 | 0.055956679 | 0.009025271 | 0.032490975 |
| Q8N158 | 10067 | GPC2 | Glypican-2 [Cleav | 530 | 1 | 530 | 0.013207547 | 0.030188679 | 0.021698113 |
| Q12891 | 10068 | HYAL2 | Hyaluronidase-2 ( | 427 | 1 | 427 | 0.018735363 | 0.018735363 | 0.018735363 |
| Q8NFP4 | 10069 | MDGA1 | MAM domain-con | 913 | 1 | 913 | 0.033953998 | 0.023001095 | 0.028477547 |
| Q7Z553 | 10070 | MDGA2 | MAM domain-con | 905 | 1 | 905 | 0.044198895 | 0.020994475 | 0.032596685 |
| O94772 | 10071 | LY6H | Lymphocyte antig | 89 | 1 | 89 | 0.04494382 | 0.011235955 | 0.028089888 |
| Q17RY6 | 10072 | LY6K | Lymphocyte antig | 120 | 1 | 120 | 0.05 | 0.016666667 | 0.033333333 |
| H3BQJ8 | 10073 | LY6L | Lymphocyte antig | 100 | 1 | 100 | 0.05 | 0.01 | 0.03 |
| P78333 | 10074 | GPC5 | Glypican-5 [Cleav | 527 | 1 | 527 | 0.018975332 | 0.051233397 | 0.035104364 |
| Q12860 | 10075 | CNTN1 | Contactin-1 (Glyc | 972 | 1 | 972 | 0.037037037 | 0.011316872 | 0.024176955 |
| Q9P232 | 10076 | CNTN3 | Contactin-3 (Brair | 982 | 1 | 982 | 0.048879837 | 0.021384929 | 0.035132383 |
| Q8IWV2 | 10077 | CNTN4 | Contactin-4 (Brair | 981 | 1 | 981 | 0.057084608 | 0.02038736 | 0.038735984 |
| O94779 | 10078 | CNTN5 | Contactin-5 (Neur | 1053 | 1 | 1053 | 0.047483381 | 0.01994302 | 0.0337132 |
| Q9UQ52 | 10079 | CNTN6 | Contactin-6 (Neur | 979 | 1 | 979 | 0.038815117 | 0.016343207 | 0.027579162 |
| Q8IVL8 | 10080 | CPO | Carboxypeptidase | 331 | 1 | 331 | 0.03021148 | 0.024169184 | 0.027190332 |
| P09326 | 10081 | CD48 | CD48 antigen (B- | 193 | 1 | 193 | 0.046632124 | 0.025906736 | 0.03626943 |
| O95971 | 10082 | CD160 | CD160 antigen (N | 268 | 2 | 134 | 0.059701493 | 0.029850746 | 0.044776119 |
| O75015 | 10083 | FCGR3B | Low affinity immu | 183 | 1 | 183 | 0.049180328 | 0.032786885 | 0.040983607 |
| Q9H4B8 | 10084 | DPEP3 | Dipeptidase 3 (EC | 427 | 1 | 427 | 0.06088993 | 0.058548009 | 0.05971897 |
| Q14002 | 10085 | CEACAM7 | Carcinoembryonic | 206 | 1 | 206 | 0.160194175 | 0.029126214 | 0.094660194 |
| P26992 | 10086 | CNTFR | Ciliary neurotroph | 319 | 1 | 319 | 0.097178683 | 0.053291536 | 0.07523511 |
| Q6YHK3 | 10087 | CD109 | CD109 antigen (1 | 1398 | 1 | 1398 | 0.08297568 | 0.01788269 | 0.050429185 |
| Q10588 | 10088 | BST1 | ADP-ribosyl cycla | 264 | 1 | 264 | 0 | 0.018939394 | 0.009469697 |
| Q9H4A9 | 10089 | DPEP2 | Dipeptidase 2 (EC | 430 | 1 | 430 | 0.048837209 | 0.058139535 | 0.053488372 |
| P38567 | 10090 | SPAM1 | Hyaluronidase PH | 454 | 1 | 454 | 0.033039648 | 0.015418502 | 0.024229075 |
| Q13449 | 10091 | LSAMP | Limbic system-as | 286 | 1 | 286 | 0.066433566 | 0.027972028 | 0.047202797 |
| P56159 | 10092 | GFRA1 | GDNF family rece | 404 | 1 | 404 | 0.03960396 | 0.047029703 | 0.043316832 |
| Q8N6Q3 | 10093 | CD177 | CD177 antigen (H | 386 | 1 | 386 | 0.044041451 | 0.028497409 | 0.03626943 |
| O00451 | 10094 | GFRA2 | GDNF family rece | 422 | 1 | 422 | 0.05450237 | 0.061611374 | 0.058056872 |
| Q9Y625 | 10095 | GPC6 | Glypican-6 [Cleav | 505 | 1 | 505 | 0.021782178 | 0.041584158 | 0.031683168 |
| Q6UWN5 | 10096 | LYPD5 | Ly6/PLAUR doma | 199 | 1 | 199 | 0 | 0.08040201 | 0.040201005 |
| O60609 | 10097 | GFRA3 | GDNF family rece | 342 | 1 | 342 | 0.035087719 | 0.040935673 | 0.038011696 |
| Q8IV16 | 10098 | GPIHBP1 | Glycosylphosphat | 130 | 1 | 130 | 0.084615385 | 0.069230769 | 0.076923077 |
| Q8NI32 | 10099 | LYPD6B | Ly6/PLAUR doma | 124 | 1 | 124 | 0 | 0.016129032 | 0.008064516 |
| P54826 | 10100 | GAS1 | Growth arrest-spe | 278 | 1 | 278 | 0.017985612 | 0.043165468 | 0.03057554 |
| O75487 | 10101 | GPC4 | Glypican-4 (K-gly | 510 | 1 | 510 | 0.011764706 | 0.041176471 | 0.026470588 |
| Q14210 | 10102 | LY6D | Lymphocyte antig | 77 | 1 | 77 | 0.051948052 | 0 | 0.025974026 |
| Q16553 | 10103 | LY6E | Lymphocyte antig | 80 | 1 | 80 | 0.075 | 0.025 | 0.05 |
| Q7RTW8 | 10104 | OTOA | Otoancorin | 1107 | 1 | 1107 | 0.028906956 | 0.043360434 | 0.036133695 |
| Q6UXB3 | 10105 | LYPD2 | Ly6/PLAUR doma | 80 | 1 | 80 | 0 | 0.0125 | 0.00625 |
| Q8N2G4 | 10106 | LYPD1 | Ly6/PLAUR doma | 96 | 1 | 96 | 0 | 0.020833333 | 0.010416667 |
| Q13508 | 10107 | ART3 | Ecto-ADP-ribosylt | 335 | 1 | 335 | 0 | 0.020895522 | 0.010447761 |
| Q6UWM5 | 10108 | GLIPR1L1 | GLIPR1-like prote | 198 | 1 | 198 | 0.025252525 | 0.025252525 | 0.025252525 |
| Q496H8 | 10109 | NRN1L | Neuritin-like prote | 103 | 1 | 103 | 0.019417476 | 0.009708738 | 0.014563107 |
| O95274 | 10110 | LYPD3 | Ly6/PLAUR doma | 295 | 1 | 295 | 0 | 0.105084746 | 0.052542373 |
| Q9Y2I2 | 10111 | NTNG1 | Netrin-G1 (Lamin | 481 | 1 | 481 | 0.033264033 | 0.031185031 | 0.032224532 |
| Q96CW9 | 10112 | NTNG2 | Netrin-G2 (Lamin | 489 | 1 | 489 | 0.028629857 | 0.044989775 | 0.036809816 |
| Q9NPD7 | 10113 | NRN1 | Neuritin | 88 | 1 | 88 | 0.034090909 | 0 | 0.017045455 |
| P23515 | 10114 | OMG | Oligodendrocyte-r | 392 | 1 | 392 | 0.12755102 | 0.104591837 | 0.116071429 |
| O95497 | 10115 | VNN1 | Pantetheinase (E( | 469 | 1 | 469 | 0.040511727 | 0.014925373 | 0.02771855 |

| | | | | | | | | |
|---|---|---|---|---|---|---|---|---|
| O95980 | 10116 | RECK | Reversion-inducin | 919 | 1 | 919 | 0.020674646 | 0.041349293 | 0.03101197 |
| Q9P121 | 10117 | NTM | Neurotrimin (hNT) | 287 | 1 | 287 | 0.055749129 | 0.027874564 | 0.041811847 |
| O43653 | 10118 | PSCA | Prostate stem cell | 74 | 1 | 74 | 0.040540541 | 0.027027027 | 0.033783784 |
| O14798 | 10119 | TNFRSF10C | Tumor necrosis fa | 210 | 1 | 210 | 0.157142857 | 0.176190476 | 0.166666667 |
| Q9Y6M0 | 10120 | PRSS21 | Testisin (EC 3.4.2 | 246 | 1 | 246 | 0.048780488 | 0.008130081 | 0.028455285 |
| O43895 | 10121 | XPNPEP2 | Xaa-Pro aminope | 627 | 1 | 627 | 0 | 0.020733652 | 0.010366826 |
| O43921 | 10122 | EFNA2 | Ephrin-A2 (EPH-r | 163 | 1 | 163 | 0.018404908 | 0.030674847 | 0.024539877 |
| P52797 | 10123 | EFNA3 | Ephrin-A3 (EFL-2) | 191 | 1 | 191 | 0.036649215 | 0.041884817 | 0.039267016 |
| P52798 | 10124 | EFNA4 | Ephrin-A4 (EPH-r | 144 | 1 | 144 | 0.027777778 | 0.027777778 | 0.027777778 |
| P52803 | 10125 | EFNA5 | Ephrin-A5 (AL-1) | 182 | 1 | 182 | 0.010989011 | 0.027472527 | 0.019230769 |

| |
|---|
| |
| |
| |
